# A Systematic Review with Meta-analysis of the Association between Changes in Muscle Strength and Clinical Outcome Changes in Patellofemoral Pain

**DOI:** 10.1007/s40279-026-02473-w

**Published:** 2026-06-16

**Authors:** Guilherme S. Nunes, Matheus Weide Solner, Jênifer de Oliveira, Bruna Wageck, Anna Julia Meireles Dangui

**Affiliations:** 1 Department of Physiotherapy and Rehabilitation, Federal University of Santa Maria, Av. Roraima, 1000, Santa Maria, RS CEP 97105-900 Brazil; 2 Hospital Universitário de Santa Maria (HUSM), Santa Maria, RS Brazil

## Abstract

**Objective:**

To investigate whether muscle strength changes observed in randomised controlled trials involving people with patellofemoral pain (PFP) are associated with concurrent changes in pain and physical function.

**Design:**

Systematic review with meta-analysis, applying a two-stage structural equation modelling approach with random-effects estimation.

**Eligibility Criteria:**

Randomised clinical trials assessing non-surgical and non-pharmacological interventions that reported both muscle strength and at least one clinical outcome (pain or function) in people with PFP.

**Data Sources:**

CINAHL, Cochrane Library, Embase, Medline and SPORTDiscus were searched from inception to April 2026.

**Results:**

From 16,750 records screened, 82 trials met the eligibility criteria (4023 participants). Regarding self-reported pain outcomes, low to moderate evidence certainty indicated significant associations between strength improvements and pain reduction for knee extensors (*r* =  − 0.75, *β* =  − 0.21), knee flexors (*r* =  − 0.46, *β* =  − 0.14), hip abductors (*r* =  − 0.91, *β* =  − 0.31), hip adductors (*r* =  − 0.26, *β* =  − 0.19), hip external rotators (*r* =  − 0.24, *β* =  − 0.06), hip internal rotators (*r* =  − 0.79, *β* =  − 0.31) and hip extensors (*r* =  − 0.58, *β* =  − 0.26). Regarding self-reported function, low to high evidence certainty indicated that strength improvements in knee extensors (*r* = 0.70, *β* = 0.15), knee flexors (*r* = 0.46, *β* = 0.07), hip abductors (*r* = 0.94, *β* = 0.15), hip adductors (*r* = 0.55, *β* = 0.11), and hip internal rotators (*r* = 0.98, *β* = 0.06) were significantly associated with improved function.

**Conclusions:**

This review provides evidence that improvements in lower limb muscle strength are associated with improvements in pain and physical function among people with PFP. These findings reinforce the clinical relevance of strength-focused rehabilitation, particularly targeting the knee and hip muscles.

**Registration Number:**

CRD42023420875.

**Supplementary Information:**

The online version contains supplementary material available at https://doi.org/10.1007/s40279-026-02473-w.

## Key Points

**Table:** 

Increases in lower limb muscle strength are consistently associated with reductions in pain and improvements in physical function in people with patellofemoral pain.
The associations suggest that muscle strength gains may represent a clinically relevant correlate of improvement in people with patellofemoral pain.
Strength gains in the knee extensors and hip abductors demonstrated the most consistent associations, supporting their potential clinical relevance within rehabilitation programmes for patellofemoral pain.

## Introduction

Patellofemoral pain (PFP) affects the general population, with higher incidence observed in specific subgroups such as adolescents, females and military personnel [1]. Although the aetiology and risk factors of PFP remain unclear, a systematic review identified reduced quadriceps muscle strength as the most consistent risk factor for the development of PFP in military populations [2]. Despite ongoing uncertainty regarding muscle weakness as a causative factor, it remains one of the most prominent clinical characteristics among people with PFP [3–5]. Robust evidence indicates that people with PFP exhibit reduced strength of the knee extensors [5] and flexors [4], as well as hip musculature [3], when compared with asymptomatic controls.

In this context, numerous studies have investigated the effects of strength-focussed interventions for the treatment of PFP [6, 7]. For example, studies by Herrington et al. [8] and Khayambashi et al. [9] compared strengthening programmes targeting the quadriceps and hip muscles, respectively, with no or minimal intervention. Both trials reported consistent improvements in symptoms and physical function among the groups that received the strengthening interventions [8, 9]. Accordingly, current clinical guidelines recommend exercise therapy, particularly protocols targeting the hip and knee muscles, as the most effective and evidence-based approach to improving pain and functional outcomes in people with PFP [6, 7, 10, 11]. However, despite the strength of this evidence base, the mechanisms by which such exercise interventions achieve their benefits remain insufficiently understood. A plausible hypothesis is that increases in muscle strength mediate the beneficial effects of rehabilitation in people with PFP. Enhanced muscle strength may promote patellofemoral joint stabilisation, correct muscular imbalances, optimise movement biomechanics and reduce joint stress, thereby mitigating pain and enhancing function [12, 13]. Nonetheless, some studies have questioned whether improvements in clinical outcomes are indeed dependent upon gains in muscle strength [14, 15]. For instance, Holden et al. [14] found that increases in hip muscle strength did not mediate reductions in pain or improvements in the number of pain-free squats among people with PFP.

These findings underscore the need to clarify whether changes in key impairments, such as muscle weakness, pain and disability, are interrelated. A clear understanding of whether, and to what extent, strength gains in specific muscle groups are linked with pain and function improvements in people with PFP will refine treatment goals and inform the development of more targeted rehabilitation strategies. If no direct association is established, high-load strengthening exercises may not be essential for clinical improvements and the mechanisms underlying the benefits of exercise therapy are likely attributable to other factors. Conversely, if a direct relationship is confirmed, rehabilitation programmes would require the deliberate inclusion of interventions aimed at increasing muscle strength. Therefore, the present systematic review aimed to examine whether changes in muscle strength reported in randomised controlled trials involving people with PFP are associated with concurrent changes in clinical outcomes, such as pain or physical function.

## Methods

### Design

This systematic review was conducted in accordance with the Cochrane Collaboration guidelines and reported following the Preferred Reporting Items for Systematic Reviews and Meta-Analyses (PRISMA) criteria (Supplementary Material A) [16]. The protocol for this review is registered in the PROSPERO database under registration number CRD42023420875.

### Deviation from Protocol

We used the Grading of Recommendations Assessment, Development and Evaluation (GRADE) approach to assess the certainty of evidence rather than the van Tulder et al. [17] recommendations because GRADE provides a more detailed, domain-based evaluation of the certainty of evidence.

### Eligibility Criteria

The inclusion criteria were as follows:

(P) Participants diagnosed with PFP, defined as people experiencing non-traumatic retropatellar or peripatellar pain exacerbated by activities that load the patellofemoral joint [18]

(I) Investigations of non-surgical and non-pharmacological interventions, to provide contexts with different patterns of change in muscle strength and clinical outcomes.

(C) Comparisons between the experimental intervention and any alternative intervention, including placebo or no intervention.

(O) Assessment of muscle strength in any muscle group, measured using dynamic or static methods and reported as quantitative data; and assessment of pain and/or function as outcomes, measured using any quantitative method.

(S) Randomised controlled trials, to minimise biases related to participant selection or intervention effects [19]

Only studies published in English were considered, with no restrictions on the publication date.

### Search Strategy

Electronic searches were conducted across five databases from their inception to April 2026: MEDLINE (Ovid), EMBASE (Elsevier), the Cochrane Library, CINAHL and SPORTDiscus (EBSCO). Search terms related to “patellofemoral pain”, “muscle strength”, “pain” and “function” were utilised to develop the search strategy (Supplementary Material B). In addition to database searches, we conducted supplementary searches through other methods, including systematic screening of reference lists of all included studies, forward citation tracking via Google Scholar and reviewing relevant systematic reviews.

### Trial Selection

Initially, duplicate records were removed using Endnote X9 (Clarivate Analytics, Philadelphia, PA, USA). Subsequently, two independent reviewers performed the study selection process, initially screening titles and abstracts, followed by full-text assessments. Discrepancies were resolved through consultation with a third reviewer. The Rayyan QCRI software (Qatar Computing Research Institute, Doha, Qatar) was used during the selection process.

### Data Extraction

Two independent reviewers extracted data from the included trials, with any discrepancies resolved through consensus. Extracted information encompassed study characteristics (setting and funding), participant characteristics (population, sample size, sex, age, symptom duration, height, weight and body mass index), muscle strength outcomes (muscle groups assessed and measurement tools), pain and/or function outcomes (assessment instruments) and intervention details (description, intervention arms, duration and intensity). When relevant statistical estimates were not reported in the original manuscripts, the corresponding authors were contacted to request the necessary data. If no response was received, a second attempt was made by contacting all authors after one week, followed by a final attempt one week later. Studies with unresolved, missing or unclear data were subsequently excluded from the synthesis.

### Risk of Bias Assessment

Two independent reviewers assessed the risk of bias (RoB) using the Cochrane Risk of Bias 2.0 tool for randomised trials, considering the effect of assignment to intervention (intention-to-treat effect) [20]. A third reviewer was consulted to resolve any disagreements. The tool evaluates biases related to the randomisation process, deviations from intended interventions, missing outcome data, outcome measurement and selection of the reported result [20, 21]. Each domain is rated as “low RoB”, “some concerns” or “high RoB”. An overall RoB judgement is assigned as follows: “low RoB” if all domains are rated as low RoB, “some concerns” if at least one domain is rated as some concerns and the rest as low RoB and “high RoB” if at least one domain is rated as high RoB or multiple domains are rated as some concerns [20].

### Data Synthesis and Analysis

Meta-analytic structural equation modelling (multivariate meta-analysis) was employed to investigate the associations between changes in muscle strength (independent variables) and changes in pain or function (dependent variables) [22, 23]. A two-stage structural equation modelling (TSSEM) approach with random-effects estimation was conducted using R statistical software 4.1.1 (package metaSEM).

#### Definitions for Analysis

(i) Each study group (regardless of intervention or control status) was treated as an individual cluster; (ii) only patient-reported outcome measures were considered for assessments of pain and function; (iii) owing to heterogeneity and the limited number of trials using algometry and performance-based function tests, meta-analyses of these data were not performed; (iv) when pain was measured using different approaches (e.g. usual, worst, etc.), the measure with the highest baseline score was used; (v) for studies assessing function using different instruments, the Anterior Knee Pain Scale (AKPS) was prioritised, as it was the most frequently reported; (vi) when muscle strength was assessed at multiple angular velocities, the velocity most commonly used across the dynamic assessments was selected for analysis, i.e. 60º/s.

#### Analytical Procedures

(i) For each cluster, we calculated effect sizes (Hedges’ *g*) and standard errors using pre- and post-intervention means and standard deviations (SDs); (ii) covariances between pain/function and muscle strength were estimated by multiplying correlation coefficients derived from prior results and standard errors [24, 25] (Supplementary Material C); (iii) as normality assumptions are not well established for meta-analytic modelling [26], histograms and Q–Q plots were generated to visually inspect the distribution of the data and identify potential outliers, which were excluded to improve the normality of the dataset; (iv) In stage 1 of the TSSEM, pooled correlation matrices were computed to evaluate the relationship between muscle strength and pain/function, stratified by muscle group; (v) stage 2 of the TSSEM was implemented to verify the significance of models—weighted least squares estimation was used to construct structural models based on the correlation and asymptotic covariance matrices [22]; (vi) sensitivity analyses examined whether pooling data across different contraction types (e.g., isometric, concentric and eccentric) influenced the observed associations; (vii) post hoc analyses explored whether pooling all included trials, irrespective of similarities in the muscle groups assessed, and selecting the muscle group with the smallest change in strength influenced the associations with clinical outcomes; (viii) considering the diversity of interventions included, subgroup analyses were conducted when the data permitted the construction of models according to the characteristics of the interventions: (a) type of interventions, categorised as physical exercise (i.e. the majority of protocol involved exercise), multimodal programmes (i.e. protocols combining exercise with other interventions) and non-exercise-based interventions (i.e. interventions unlikely to elicit changes in muscle strength); and (b) type of resistance training on the basis of intensity, classified as endurance (< 50% of one repetition maximum), strength (≥ 50% of one repetition maximum) and power (repetitions performed at an explosive velocity) [27] – in cases where intensity was not reported, classification was based on the description of the training.

Correlation magnitudes were classified as very weak (*r* = 0.0–0.19), weak (*r* = 0.20–0.39), moderate (*r* = 0.40–0.59), strong (*r* = 0.60–0.79) and very strong (*r* ≥ 0.80) [28]. Heterogeneity was evaluated using the Higgins’ *I*^2^, and values above 50% were considered as substantial heterogeneity [29].

### Evidence Certainty

The GRADE approach was employed to determine the certainty of the evidence for each outcome included in the meta-analyses [30]. Two independent reviewers conducted the assessment, and any disagreements were resolved through consensus. Certainty of evidence was initially rated as high and could be downgraded to moderate, low or very low according to the following criteria: (i) risk of bias: downgraded by one level if more than 25% of the included studies presented high RoB, or by two levels if all studies in the analysis were classified as high RoB; (ii) inconsistency: downgraded by one level when both clinical and strength outcomes showed heterogeneity, defined as an *I*^2^ value greater than 50%; (iii) indirectness: no downgrading was applied, as all studies included participants with PFP, ensuring that the findings were directly relevant to the research question; (iv) imprecision: downgraded by one level when analyses included fewer than 100 participants or when non-significant associations presented confidence intervals for correlation coefficients (*r*) that crossed zero; (v) publication bias: not downgraded, as none of the included studies directly examined associations between changes in clinical outcomes and muscle strength, making it unlikely that publication bias influenced these analyses.

## Results

### Study Selection and Handling

**Fig. 1:**
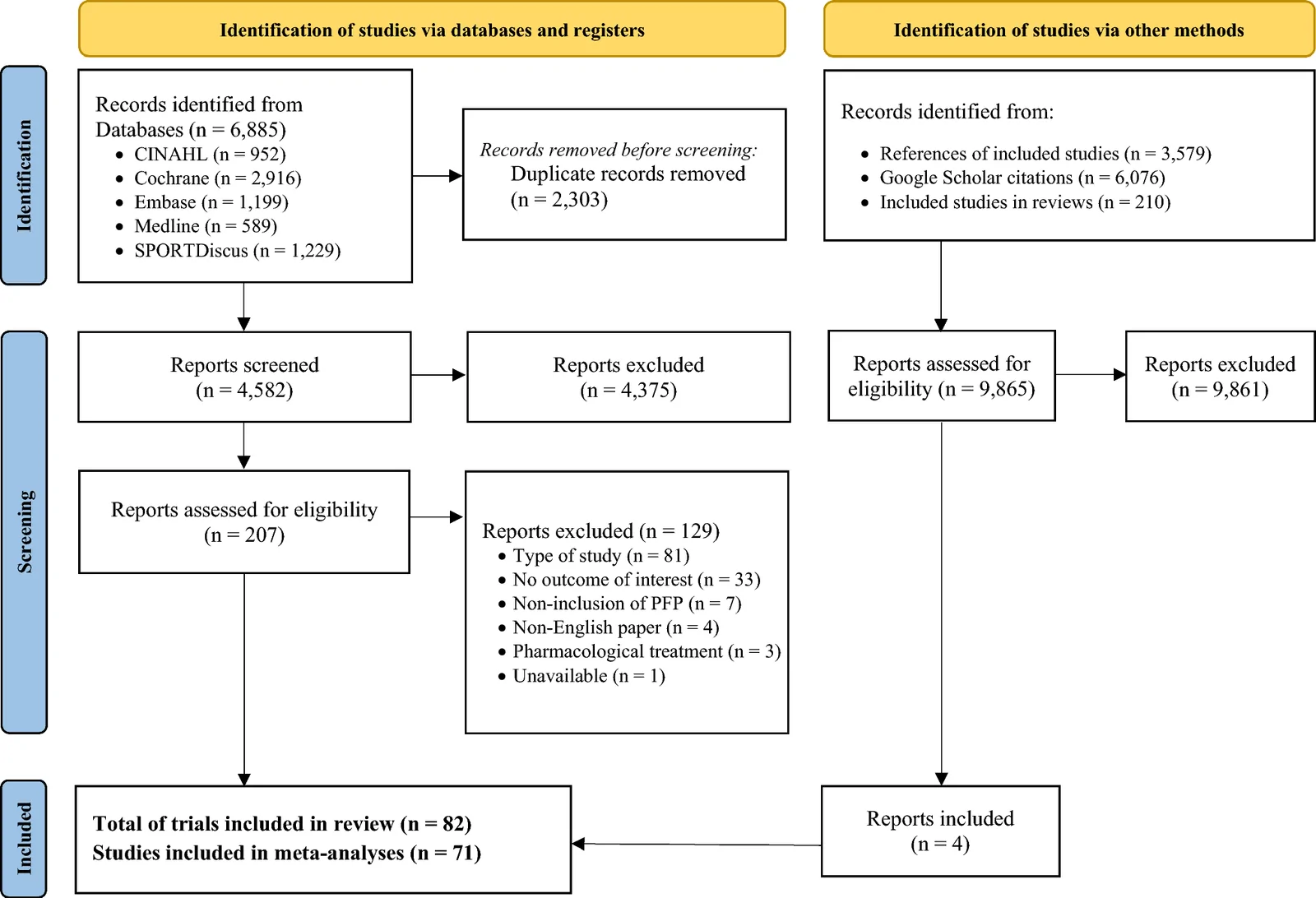
Flow diagram

The search yielded 16,750 records. Of these, 82 trials met the eligibility criteria and were included, with 71 contributing to the meta-analyses (Fig. 1). A list of excluded studies is provided in Supplementary Material D. One crossover trial was included [31], and its sample was considered as a single cluster in the analyses.

### Studies’ Characteristics

The included trials involved 4023 people with PFP (Supplementary Material E). Pain was assessed in 81 trials, and function was assessed in 60 trials using patient-reported outcome measures; 12 trials included performance-based function tests (Supplementary Material E). The trials evaluated strength in the knee extensors [8, 31–100], knee flexors [35, 36, 39, 40, 45, 48, 54–56, 58, 60, 62, 63, 70, 71, 76, 79–81, 84, 85, 90, 96, 100], hip abductors [9, 32, 33, 36, 38, 39, 42, 47, 49–51, 54, 56, 59, 60, 68, 69, 71–73, 76, 79, 83, 87–92, 99–108], hip adductors [39, 56, 76, 79, 90, 102, 103], hip extensors [33, 36, 47, 51, 54, 56, 60, 71, 73, 76, 83, 88–90, 99, 100], hip flexors [56, 76, 87, 90], hip external rotators [9, 32, 33, 38, 39, 42, 49, 51, 54, 56, 69, 71, 72, 76, 87–89, 91, 100–105, 108], hip internal rotators [33, 39, 54, 56, 76, 102, 103], hip posterolateral muscles [35, 81], lower limbs [109, 110], ankle dorsiflexors [60], ankle plantarflexors [60], ankle eversors [71], ankle inversors [71] and flexor hallucis longus [60] (Supplementary Material E).

Regarding interventions, 22 trials investigated strengthening exercises [8, 9, 33, 53, 55, 56, 59, 68, 76, 79, 80, 83, 89–91, 95–97, 100–102, 107], 64 trials investigated exercise therapy combined with other approaches [31–36, 38–42, 45–54, 57–61, 63–66, 68, 69, 71–75, 77–89, 91, 93, 94, 96, 97, 99, 100, 103–107, 109, 110], and 17 trials investigated standalone interventions, such as bandages [37, 62, 67], education [42, 46, 51, 58, 95], electrothermotherapy [43, 44, 70, 92], supplementation [9], manual therapy [98, 108–110] and stretching [76, 79] (Supplementary Material F).

### Risk of Bias

Of the 82 included studies, 16% (*n* = 13) were rated as having low RoB, 10% (*n* = 8) raised some concerns, and 74% (*n* = 61) were rated as high RoB (Supplementary Material G). The primary reasons for high RoB classifications included the absence of intention-to-treat analyses and lack of assessor blinding.

### Meta-analysis

No meta-analyses were conducted for hip flexors, hip posterolateral muscles, general lower limbs, flexor hallucis longus, ankle dorsiflexors, plantarflexors, eversors or inversors owing to the limited number of trials (Supplementary Material H).

Cluster analysis graphs and the excluded outliers are reported in Supplementary Material I. The evidence quality for all meta-analyses is reported in Supplementary Material J. All models demonstrated adequate fit, as indicated by chi-square goodness-of-fit tests (*p* > 0.05).

### Pain versus Muscle Strength

Meta-analyses were conducted for muscle strength of knee extension and flexion and hip abduction, adduction, external rotation, internal rotation and extension (Table 1). Sensitivity analyses indicated that pooling data across different contraction types did not influence the results. No sensitivity analysis was conducted for hip adduction, as the removal of any cluster rendered the model unviable. Negative *r*-values indicate that increases in muscle strength were associated with reductions in pain.

**Table 1:** Analyses for the associations between changes in pain versus muscle strength

Muscle group	*k* clusters (*n*)	Pain level [ES (CI 95%, *I*^2^)]	Muscle strength [ES (CI 95%, *I*^2^)]	Correlational coefficient [*r* (CI 95%)]	Regression coefficient [*β* (CI 95%)]
Knee extension	112 (*n* = 2594)	–1.52 (–1.71, –1.33) *I*^2^ = 89%	0.48 (0.39, 0.56) *I*^2^ = 49%	–0.75 (–0.77, –0.73)	–0.21 (–0.23, –0.20)
Knee flexion	41 (*n* = 964)	–1.45 (–1.80, –1.12) *I*^2^ = 91%	0.49 (0.35, 0.62) *I*^2^ = 51%	–0.46 (–0.51, –0.41)	–0.14 (–0.19, –0.09)
Hip abduction	70 (*n* = 1760)	–1.77 (–2.07, –1.47) *I*^2^ = 93%	0.47 (0.37, 0.57) *I*^2^ = 50%	–0.91 (–0.92, –0.90)	–0.31 (–0.32, –0.30)
Hip adduction	17 (*n* = 515)	–1.61 (–2.39, –0.82) *I*^2^ = 97%	0.20 (–0.08, 0.47) *I*^2^ = 76%	–0.26 (–0.34, –0.18)	–0.19 (–0.28, –0.12)
Hip ER	43 (*n* = 1220)	–1.81 (–2.12, –1.50) *I*^2^ = 90%	0.47 (0.35, 0.59) *I*^2^ = 47%	–0.24 (–0.29, –0.19)	–0.06 (–0.11, –0.01)
Hip IR	16 (*n* = 607)	–2.02 (–2.79, –1.25) *I*^2^ = 97%	0.24 (0.05, 0.44) *I*^2^ = 55%	–0.79 (–0.82, –0.76)	–0.31 (–0.34, –0.28)
Hip extension	31 (*n* = 945)	–1.83 (–2.31, –1.35) *I*^2^ = 91%	0.42 (0.26, 0.59) *I*^2^ = 63%	–0.58 (–0.62, –0.54)	–0.26 (–0.30, –0.22)
General	141 (*n* = 3290)	–1.52 (–1.71, –1.34) *I*^2^ = 91%	0.37 (0.29, 0.45) *I*^2^ = 64%	–0.64 (–0.66, –0.62)	–0.26 (–0.28, − 0.24)

*k* number of clusters, *n* number of participants, *ES* effect size, *CI* confidence interval, *ER* external rotation, *IR* internal rotation

#### ***Knee Extension*** (112 clusters, 60 trials [8, 31–42, 46–60, 62–72, 74–81, 83, 85, 86, 88–95, 99, 100])

Increases in strength were strongly associated with reductions in pain. Each SD increase in strength corresponded to a 0.21 SD decrease in pain (moderate evidence; Table 1). For subgroup analyses, a very strong association was observed for interventions including only strength training, strong associations for physical exercise and endurance training and a moderate association for multimodal programmes (Supplementary Material K).

#### ***Knee Flexion*** (41 clusters, 21 trials [35, 36, 39, 40, 48, 54–56, 58, 60, 62, 63, 70, 71, 76, 79–81, 85, 90, 100])

Increases in strength were moderately associated with reductions in pain. Each SD increase in strength corresponded to a 0.14 SD decrease in pain (low evidence; Table 1). For subgroup analyses, a very strong association was observed for interventions including only strength training, a strong association for multimodal programmes and a non-significant association for physical exercise interventions (Supplementary Material K).

#### ***Hip Abduction*** (70 clusters, 36 trials [9, 32, 33, 36, 38, 39, 42, 47, 49–51, 54, 56, 59, 60, 68, 69, 71, 72, 76, 79, 83, 88–92, 99–104, 106–108])

Increases in strength were very strongly associated with reductions in pain. Each SD increase in strength corresponded to a 0.31 SD decrease in pain (moderate evidence; Table 1). For subgroup analyses, very strong associations were observed for interventions including only physical exercise, endurance training or strength training and a weak association for multimodal programmes (Supplementary Material K).

#### ***Hip Adduction*** (17 clusters, 7 trials [39, 56, 76, 79, 90, 102, 103]

Increases in strength were weakly associated with reductions in pain. Each SD increase in strength corresponded to a 0.19 SD decrease in pain (low evidence; Table 1). For subgroup analyses, a non-significant association was observed for interventions including only physical exercise (Supplementary Material K).

#### ***Hip External Rotation*** (43 clusters, 23 trials [9, 32, 33, 38, 39, 42, 49, 51, 54, 56, 69, 71, 72, 76, 88, 89, 91, 100–104, 108])

Increases in strength were weakly associated with reductions in pain. Each SD increase in strength corresponded to a 0.06 SD decrease in pain (moderate evidence; Table 1). For subgroup analyses, a very weak association was observed for interventions including only strength training, and non-significant associations for physical exercise or multimodal programmes (Supplementary Material K).

#### ***Hip Internal Rotation*** (16 clusters, 7 trials [33, 39, 54, 56, 76, 102, 103]

Increases in strength were strongly associated with reductions in pain. Each SD increase in strength corresponded to a 0.31 SD decrease in pain (moderate evidence; Table 1). For subgroup analyses, a strong association was observed for interventions including only physical exercise (Supplementary Material K).

#### ***Hip Extension*** (31 clusters, 15 trials [33, 36, 47, 51, 54, 56, 60, 71, 76, 83, 88–90, 99, 100])

Increases in strength were moderately associated with reductions in pain. Each SD increase in strength corresponded to a 0.26 SD decrease in pain (low evidence; Table 1). For subgroup analyses, a strong association was observed for interventions including only physical exercise, and a non-significant association for multimodal programmes (Supplementary Material K).

#### ***Post-hoc Analysis of all Trials*** (141 clusters, 69 trials [8, 9, 31–42, 46–60, 62–72, 74–81, 83, 85, 86, 88–95, 99–104, 106–109])

**Fig. 2:**
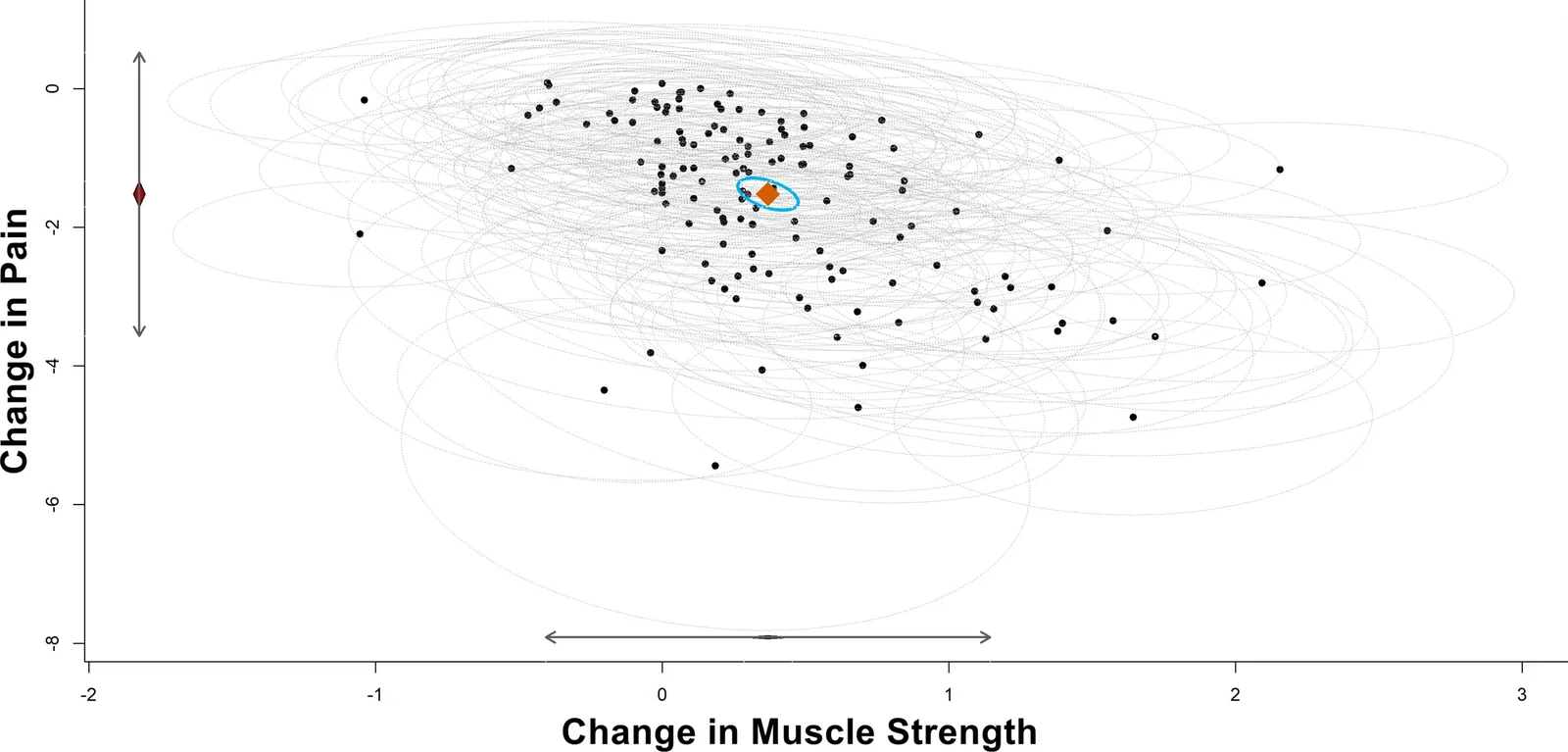
Cluster analysis of effect sizes for changes in pain versus general muscle strength. Black dots with surrounding ellipses represent the effect sizes (ES) and corresponding 95% confidence intervals (CI) for individual clusters (note: negative values in *y*-axis indicate a reduction in pain; positive values in *x*-axis indicate an increase in muscle strength). The orange diamond denotes the pooled ES for the combined outcomes, with the surrounding blue ellipses representing its 95% CI. Red diamonds adjacent to each axis, accompanied by arrows, indicate the pooled ES and 95% CI for each individual outcome

General increases in strength were moderately associated with decreases in pain. Each SD increase in strength corresponded to 0.26 SD decrease in pain (low evidence; Table 1, Fig. 2). For subgroup analyses, a very strong association was observed for interventions including only strength training, and moderate associations for physical exercise interventions, multimodal programmes or endurance training (Supplementary Material K).

### Function Versus Muscle Strength

Meta-analyses were conducted for muscle strength of knee extension and flexion, and hip abduction, adduction, external rotation, internal rotation and extension (Table 2). Sensitivity analyses indicated that, for most analyses, pooling data across different contraction types did not influence the results. The exception was hip internal rotation, where including dynamic contractions made the model unfeasible. No sensitivity analysis was performed for knee flexion and hip adduction, as the removal of any cluster rendered the models unviable. Positive *r*-values imply that greater strength gains were associated with better functionality.

**Table 2:** Analyses for the relationship between changes in function versus muscle strength

Muscle group	k Clusters (*n*)	Function [ES (CI 95%, *I*^2^)]	Muscle strength [ES (CI 95%, *I*^2^)]	Correlational coefficient [*r* (CI 95%)]	Regression coefficient [*β* (CI 95%)]
Knee extension	94 (*n* = 2301)	1.17 (1.00, 1.33) *I*^2^ = 84%	0.49 (0.40, 0.58) *I*^2^ = 51%	0.70 (0.68, 0.72)	0.15 (0.13, 0.17)
Knee flexion	33 (*n* = 800)	1.04 (0.80, 1.27) *I*^2^ = 77%	0.52 (0.37, 0.67) *I*^2^ = 45%	0.46 (0.40, 0.51)	0.07 (0.02, 0.13)
Hip abduction	62 (*n* = 1583)	1.20 (1.01, 1.49) *I*^2^ = 83%	0.33 (0.05, 0.61) *I*^2^ = 41%	0.94 (0.93, 0.95)	0.15 (0.15, 0.16)
Hip adduction	12 (*n* = 353)	1.10 (0.71, 1.39) *I*^2^ = 78%	0.48 (0.38, 0.58) *I*^2^ = 62%	0.55 (0.47, 0.62)	0.11 (0.04, 0.18)
Hip ER	42 (*n* = 1218)	1.10 (0.93, 1.28) *I*^2^ = 73%	0.46 (0.33, 0.58) *I*^2^ = 50%	0.34 (0.29, 0.39)	0.04 (*− *0.01, 0.09)*
Hip IR	12 (*n* = 512)	1.10 (0.82, 1.37) *I*^2^ = 66%	0.19 (*− *0.01, 0.39) *I*^2^ = 42%	0.98 (0.97, 0.99)	0.06 (0.05, 0.07)
Hip extension	30 (*n* = 895)	1.10 (0.91, 1.29) *I*^2^ = 66%	0.38 (0.25, 0.51) *I*^2^ = 31%	0.05 (*− *0.01, 0.12)	0.00 (*− *0.06, 0.07)*
General	111 (*n* = 2626)	1.09 (0.90, 1.27) *I*^2^ = 89%	0.36 (0.27, 0.44) *I*^2^ = 55%	0.83 (0.82, 0.84)	0.25 (0.24, 0.27)

*Non-significant association

*k* number of clusters, *n* number of participants, *ES* effect size, *CI* confidence interval, *ER* external rotation, *IR* internal rotation

#### ***Knee Extension*** (94 clusters, 47 trials [8, 32–36, 38, 39, 41, 42, 45–54, 56–60, 62–65, 68, 70–73, 75–77, 83, 85, 88–91, 93–95, 100])

Increases in strength were strongly associated with increases in function. Each SD increase in strength corresponded to a 0.15 SD increase in function (moderate evidence; Table 2). For subgroup analyses, a very strong association was observed for interventions including only strength training, a strong association for physical exercise interventions, a moderate association for endurance training, and a non-significant association for multimodal programmes (Supplementary Material K).

#### ***Knee Flexion*** (33 clusters, 17 trials [35, 36, 39, 45, 48, 54, 56, 58, 60, 62, 63, 70, 71, 76, 85, 90, 100]

Increases in strength were moderately associated with increases in function. Each SD increase in strength corresponded to a 0.07 SD increase in function (moderate evidence; Table 2). For subgroup analyses, a non-significant association was observed for interventions including only physical exercise interventions (Supplementary Material K).

#### ***Hip Abduction*** (62 clusters, 30 trials [9, 32, 33, 36, 38, 39, 42, 47, 49–51, 54, 56, 59, 60, 68, 71–73, 76, 83, 88–91, 100–102, 104, 106]

Increases in strength were very strongly associated with increases in function. Each SD increase in strength was linked to a 0.15 SD increase in function (moderate evidence; Table 2). For subgroup analyses, very strong associations were observed for interventions including only physical exercise interventions or endurance training, and a non-significant association for multimodal programmes (Supplementary Material K).

#### ***Hip Adduction*** (12 clusters, 5 trials [39, 56, 76, 90, 102])

Increases in strength were moderately associated with increases in function. Each SD increase in strength was linked to a 0.11 SD increase in function (moderate evidence; Table 2). For subgroup analyses, a non-significant association was observed for interventions including only physical exercise (Supplementary Material K).

#### ***Hip External Rotation*** (42 clusters, 20 trials [9, 32, 33, 38, 39, 42, 49, 51, 54, 56, 71, 72, 76, 88, 89, 91, 100–102, 104]

There was no association between changes in strength and changes in function (high evidence; Table 2). For subgroup analyses, a non-significant association was observed for interventions including only physical exercise, strength training or multimodal programmes (Supplementary Material K).

#### ***Hip Internal Rotation*** (12 clusters, 6 trials [33, 39, 54, 56, 76, 102])

Increases in isometric strength were very strongly associated with increases in function. Each SD increase in strength corresponded to a 0.06 SD increase in function (high evidence; Table 2). For subgroup analyses, very strong associations were observed for interventions including only physical exercise interventions or strength training (Supplementary Material K).

#### ***Hip Extension*** (30 clusters, 15 trials [33, 36, 47, 51, 54, 56, 60, 71, 73, 76, 83, 88–90, 100])

There was no association between changes in strength and changes in function (moderate evidence; Table 2). For subgroup analyses, a non-significant association was observed for interventions including only physical exercise (Supplementary Material K).

#### Post Hoc*** Analysis of All Trials*** (111 clusters, 53 trials [8, 9, 32–36, 38, 39, 41, 42, 45–54, 56–60, 62–65, 68, 70–73, 75–77, 83, 85, 88–91, 93–95, 100–102, 104, 106, 109])

**Fig. 3:**
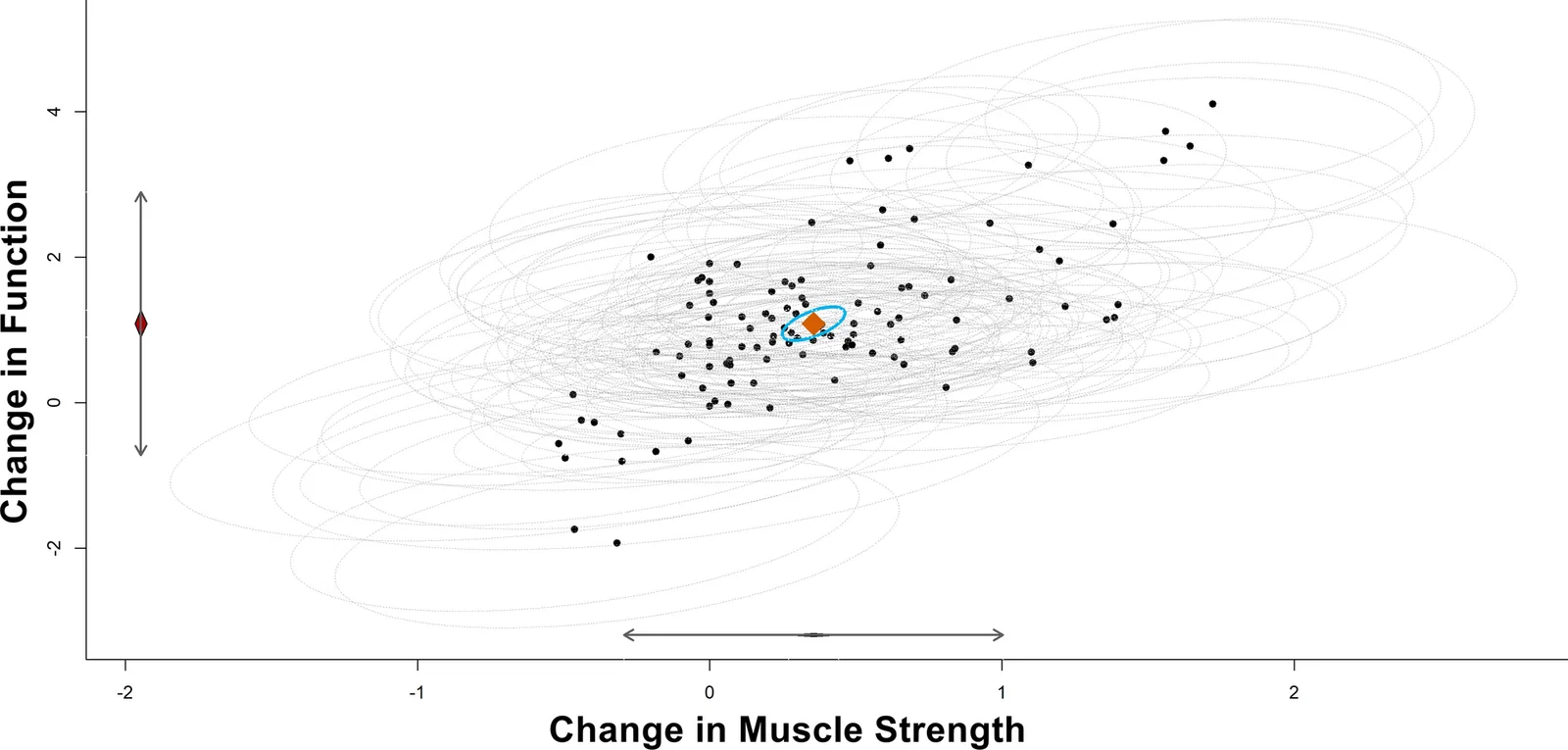
Cluster analysis of effect sizes for changes in function versus general muscle strength. Black dots with surrounding ellipses represent the effect sizes (ES) and corresponding 95% confidence intervals (CI) for individual clusters (note: positive values indicate an increase in function or muscle strength). The orange diamond denotes the pooled ES for the combined outcomes, with the surrounding blue ellipses representing its 95% CI. Red diamonds adjacent to each axis, accompanied by arrows, indicate the pooled ES and 95% CI for each individual outcome

General increases in strength were very strongly associated with increases in function. Each SD increase in strength corresponded to 0.25 SD increase in function (low evidence; Table 2, Fig. 3). For subgroup analyses, very strong associations were observed for interventions including only physical exercise or endurance training, a moderate association for strength training, and a non-significant association for multimodal programmes (Supplementary Material K).

## Discussion

This review provides low- to high-certainty evidence that changes in lower limb muscle strength are associated with improvements in pain and function in people with PFP. Specifically, strength improvements in the knee extensors and flexors, as well as the hip abductors, adductors, extensors, external and internal rotators, were significantly associated with reduced pain. Similar associations with improved function were observed for the knee extensors and flexors, and the hip abductors, adductors and internal rotators. The magnitude of these associations ranged from very strong to weak. Post hoc analyses informed further insight by reporting that even muscle groups exhibiting relatively small strength changes across trials still demonstrated consistent associations with improvements in clinical outcomes.

These findings suggest that changes in muscle strength are meaningfully associated with clinical improvement in PFP. While the analyses do not permit conclusions regarding directionality or causality, several physiological mechanisms related to strength development may provide biological plausibility for the observed associations between strength gains and improvements in pain and function. Achieving measurable increases in strength typically requires specific and progressive overload [111], implying a deliberate stimulus beyond spontaneous symptom relief. Although reductions in pain may facilitate neuromuscular activation via disinhibition, it is unlikely that pain relief alone accounts for substantial strength gains. It is also possible that exercise interventions yield benefits across multiple domains (e.g. strength, pain and function) via shared neuromechanical, psychological or general training-related pathways, independent of strength improvements per se. Factors such as symptom modulation, load adaptation and broader recovery processes may contribute to clinical improvement in PFP. Therefore, clinical improvement in PFP may occur even in the absence of substantial strength gains, suggesting that strength changes may represent one correlate of recovery rather than the sole driver of symptom improvement. It is important to emphasise that, owing to the observational nature of the data and the statistical approach employed, we cannot infer causality or confirm that muscle strength gains mediate improvements in clinical outcomes. Nonetheless, the findings indicate consistent evidence that changes in muscle strength are meaningfully linked to improvements in both pain and function in people with PFP.

Previous biomechanical theories have proposed that knee muscle weakness may contribute to alterations in patellar tracking and malalignment in people with PFP, leading to increased joint stress and symptom aggravation [12, 13]. Similarly, weakness in the hip muscles may contribute to excessive femoral internal rotation and hip adduction, increasing lateral patellar forces [12, 13]. Our findings align with these biomechanical models, suggesting that improvements in muscle strength, particularly of the knee extensors, hip abductors and hip internal rotators, may be associated with favourable changes in movement patterns and joint loading, which could relate to improvements in pain and function. This interpretation is consistent with recent evidence demonstrating associations between changes in hip and knee frontal plane kinematics and symptom relief in people with PFP [16]. Nonetheless, further research is warranted to determine whether movement alterations mediate the relationship between hip muscle strength gains and improvements in clinical outcomes.

Physiological mechanism underpinning muscle strength development may also provide plausible explanatory models for the observed associations. Given that approximately 80% of included trials implemented interventions lasting fewer than 8 weeks, hypertrophic adaptations are unlikely to be the primary driver of strength changes [112]. Neural adaptations, such as improved motor unit recruitment and firing synchrony, are more plausible mechanisms in this timeframe [113]. Consequently, enhanced motor control may represent one potential explanation for the observed associations between strength development and clinical improvements. Greater recruitment of muscle fibres may be associated with improved activation of structures responsible for movement control, while potentially influencing nociceptive or central pathways involved in pain processing [114]. Another mechanism relevant to strength development is the mechanotransduction pathway [115]. Considering that people with PFP may exhibit signs of low-grade inflammation [116, 117], it is plausible that interventions involving muscle contractions aimed at increasing strength may also be associated with analgesic responses potentially mediated through mechanotransduction pathways [115].

Clinically, these findings reinforce current guidelines that advocate exercise therapy targeting knee and hip musculature as a first-line intervention for PFP [6, 7]. Additionally, the subgroup analyses demonstrated particularly strong associations of clinical improvements with muscle strength, especially within strength-based exercise programmes. These results support the potential clinical relevance of these muscle groups within rehabilitation programmes. In contrast, the lack of association between hip extension and external rotation strength with function outcomes indicates that its role may be less prominent than previously thought, suggesting that its emphasis in practice could warrant further consideration. Although this review did not directly assess treatment efficacy, consistent associations across endurance and strength training protocols, even with relatively short intervention durations, suggest that even modest strength gains may be associated with clinically meaningful improvements. This suggests that strict hypertrophy-oriented programmes may not be essential for symptom relief. Instead, clinicians can reasonably adopt a broader and more inclusive exercise approach, focusing on progressive strengthening tailored to individual needs and tolerance. Nevertheless, exercise prescription should remain structured and individualised to ensure appropriate strength adaptations and optimise patient outcomes.

Several aspects of this review warrant cautious interpretation. This analysis did not aim to evaluate the effectiveness of specific interventions or to establish causal relationships, but rather to explore how changes in clinical outcomes relate to changes in muscle strength. To this end, a broad range of interventions was included to reflect diverse clinical contexts and to identify potential patterns between strength adaptations and improvements in pain and function. As such, the findings should not be interpreted in relation to individual intervention effects, since factors such as dosage and duration of interventions were not controlled. Despite the use of robust statistical modelling, certain limitations must be acknowledged. To estimate covariance values, we relied on cross-sectional data from previous studies. Although this is a recognised and valid approach [24], it may introduce estimation bias and could influence the magnitude of observed associations. Additionally, pooling data from different contraction types may not have been entirely appropriate. Sensitivity analyses generally confirmed that contraction type did not substantially alter the results; however, subgroup differences emerged, particularly for hip internal rotation, where isometric versus dynamic testing appeared to affect the strength of the associations. This suggests that, for this specific muscle group, the method of strength assessment, and potentially the mode of training, may influence its relationship with clinical outcomes. Finally, although most results are based on moderate certainty of evidence, most included trials were rated as having a high RoB, and significant heterogeneity was observed across analyses. These factors limit the certainty of the findings and underscore the need for future high-quality trials to validate and extend these results.

## Conclusions

This review provides moderate evidence that improvements in lower limb muscle strength are associated with reductions in self-reported pain and enhancements in self-reported physical function in people with PFP. These findings suggest that increases in lower limb muscle strength may represent an important correlate of clinical improvement in people with PFP.

## Supplementary Information

Below is the link to the electronic supplementary material.

Supplementary file1 (PDF 4529 KB)

## Declarations

### Data Availability

All data supporting the findings of this study are available within the paper and its Supplementary Information.
