# Supplementary material for: A Systematic Review with Meta-analysis of the Association between Changes in Muscle Strength and Clinical Outcome Changes in Patellofemoral Pain
Source: Sports Med. 2026 Jun 16;56(11):2717–31. doi: 10.1007/s40279-026-02473-w (PMC13672437; doi:10.1007/s40279-026-02473-w)
Supplement: Supplementary file 1 [file 40279_2026_2473_MOESM1_ESM.pdf]

SUPPLEMENTARY MATERIAL

Supplementary Material A. PRISMA checklist 1

Supplementary Material B. Strategy of search 4

Supplementary Material C. Correlation coefficients used for covariance calculations 9

Supplementary Material D. Excluded studies after full-text screening with reasons 11

Supplementary Material E. Individual characteristics of the included interventions. ... 26

Supplementary Material F. Characterization of the included interventions 34

Supplementary Material G. Risk of bias for the included studies using the RoB 2.0 63

Supplementary Material H. Individual data of the included studies 65

Supplementary Material I. Cluster analysis graphs 98

Supplementary Material J. GRADE approach for evidence quality assessment 106

Supplementary Material K. Subgroup analyses 107

Supplementary Material A. PRISMA checklist.

Section and Topic	Item #	Checklist item	Location where item is reported
**TITLE**			
Title	1	Identify the report as a systematic review.	1
**ABSTRACT**			
Abstract	2	See the PRISMA 2020 for Abstracts checklist.	2
**INTRODUCTION**			
Rationale	3	Describe the rationale for the review in the context of existing knowledge.	3
Objectives	4	Provide an explicit statement of the objective(s) or question(s) the review addresses.	4
**METHODS**			
Eligibility criteria	5	Specify the inclusion and exclusion criteria for the review and how studies were grouped for the syntheses.	4
Information sources	6	Specify all databases, registers, websites, organisations, reference lists and other sources searched or consulted to identify studies. Specify the date when each source was last searched or consulted.	5
Search strategy	7	Present the full search strategies for all databases, registers and websites, including any filters and limits used.	5
Selection process	8	Specify the methods used to decide whether a study met the inclusion criteria of the review, including how many reviewers screened each record and each report retrieved, whether they worked independently, and if applicable, details of automation tools used in the process.	5
Data collection process	9	Specify the methods used to collect data from reports, including how many reviewers collected data from each report, whether they worked independently, any processes for obtaining or confirming data from study investigators, and if applicable, details of automation tools used in the process.	5
Data items	10a	List and define all outcomes for which data were sought. Specify whether all results that were compatible with each outcome domain in each study were sought (e.g. for all measures, time points, analyses), and if not, the methods used to decide which results to collect.	5
	10b	List and define all other variables for which data were sought (e.g. participant and intervention characteristics, funding sources). Describe any assumptions made about any missing or unclear information.	5
Study risk of bias assessment	11	Specify the methods used to assess risk of bias in the included studies, including details of the tool(s) used, how many reviewers assessed each study and whether they worked independently, and if applicable, details of automation tools used in the process.	5-6
Effect measures	12	Specify for each outcome the effect measure(s) (e.g. risk ratio, mean difference) used in the synthesis or presentation of results.	6-7
Synthesis methods	13a	Describe the processes used to decide which studies were eligible for each synthesis (e.g. tabulating the study intervention characteristics and comparing against the planned groups for each synthesis (item #5)).	6-7
	13b	Describe any methods required to prepare the data for presentation or synthesis, such as handling of missing summary statistics, or data conversions.	6-7
	13c	Describe any methods used to tabulate or visually display results of individual studies and syntheses.	6-7
	13d	Describe any methods used to synthesize results and provide a rationale for the choice(s). If meta-analysis was performed, describe the model(s), method(s) to identify the presence and extent of statistical heterogeneity, and software package(s) used.	6-7

Section and Topic	Item #	Checklist item	Location where item is reported
	13e	Describe any methods used to explore possible causes of heterogeneity among study results (e.g. subgroup analysis, meta-regression).	7
	13f	Describe any sensitivity analyses conducted to assess robustness of the synthesized results.	7
Reporting bias assessment	14	Describe any methods used to assess risk of bias due to missing results in a synthesis (arising from reporting biases).	NA
Certainty assessment	15	Describe any methods used to assess certainty (or confidence) in the body of evidence for an outcome.	7
**RESULTS**			
Study selection	16a	Describe the results of the search and selection process, from the number of records identified in the search to the number of studies included in the review, ideally using a flow diagram.	8
	16b	Cite studies that might appear to meet the inclusion criteria, but which were excluded, and explain why they were excluded.	8
Study characteristics	17	Cite each included study and present its characteristics.	8-9
Risk of bias in studies	18	Present assessments of risk of bias for each included study.	9
Results of individual studies	19	For all outcomes, present, for each study: (a) summary statistics for each group (where appropriate) and (b) an effect estimate and its precision (e.g. confidence/credible interval), ideally using structured tables or plots.	8-9
Results of syntheses	20a	For each synthesis, briefly summarise the characteristics and risk of bias among contributing studies.	9-14
	20b	Present results of all statistical syntheses conducted. If meta-analysis was done, present for each the summary estimate and its precision (e.g. confidence/credible interval) and measures of statistical heterogeneity. If comparing groups, describe the direction of the effect.	9-14
	20c	Present results of all investigations of possible causes of heterogeneity among study results.	9-14
	20d	Present results of all sensitivity analyses conducted to assess the robustness of the synthesized results.	9-14
Reporting biases	21	Present assessments of risk of bias due to missing results (arising from reporting biases) for each synthesis assessed.	NA
Certainty of evidence	22	Present assessments of certainty (or confidence) in the body of evidence for each outcome assessed.	9-14
**DISCUSSION**			
Discussion	23a	Provide a general interpretation of the results in the context of other evidence.	15
	23b	Discuss any limitations of the evidence included in the review.	16-17
	23c	Discuss any limitations of the review processes used.	16-17
	23d	Discuss implications of the results for practice, policy, and future research.	16
**OTHER INFORMATION**			

Section and Topic	Item #	Checklist item	Location where item is reported
Registration and protocol	24a	Provide registration information for the review, including register name and registration number, or state that the review was not registered.	4
	24b	Indicate where the review protocol can be accessed, or state that a protocol was not prepared.	4
	24c	Describe and explain any amendments to information provided at registration or in the protocol.	4
Support	25	Describe sources of financial or non-financial support for the review, and the role of the funders or sponsors in the review.	Title page
Competing interests	26	Declare any competing interests of review authors.	Title page
Availability of data, code and other materials	27	Report which of the following are publicly available and where they can be found: template data collection forms; data extracted from included studies; data used for all analyses; analytic code; any other materials used in the review.	Title page

Supplementary Material B. Strategy of search.

Medline (via Ovid)

1.	Patellofemoral Joint/
2.	Patella/
3.	Patellofemoral Pain Syndrome/
4.	Chondromalacia Patellae/
5.	(anterior knee pain).tw
6.	((patell* or femoropatell* or femoro-patell* or retropatell*) adj2 (pain or syndrome or dysfunction)).tw
7.	((chondromalac* or chondropath* or chondrosis or arthralgia) adj2 (knee* or patell* or femoropatell* or femoro-patell* or retropatell*)).tw
8.	((lateral compression or lateral facet or lateral pressure or odd facet) adj2 (syndrome or pain)).tw
9.	or/1-8
10.	Muscle Strength Dynamometer/
11.	Muscle Strength/
12.	(strength OR torque OR force OR moment* OR isokinetic OR isometric OR eccentric OR concentric OR power OR isotonic OR endurance OR kinetic* OR hand-held or dynamomet*).tw
13.	or/10-12
14.	Pain/
15.	Pain Measurement/
16.	Musculoskeletal Pain/
17.	Rehabilitation/
18.	Recovery of Function/
19.	Patient Outcome Assessment/
20.	(pain* or discomfor* or function*).ab,ti
21.	(squat* or jump* or hop* or drop* or run* or walk* or stair or step* or cut* or sit* or stand* or task or performance).ab,ti
22.	(scale or questionnaire).tw
23.	or/14-22
24.	Clinical trials as topic/
25.	Randomized Controlled Trial/
26.	Controlled Clinical Trials as Topic/
27.	Randomized controlled trial.pt
28.	Controlled clinical trial.pt
29.	randomi#ed.ab
30.	placebo.ab
31.	randomly.ab
32.	trial.ti
33.	((clinical or controlled or comparative or placebo or prospective* or randomi#ed) adj3 (trial or study)).tw
34.	(random* adj7 (allocat* or allot* or assign* or basis* or divid* or order*)).tw
35.	((singl* or doubl* or trebl* or tripl*) adj7 (blind* or mask*)).tw
36.	(cross?over* or (cross adj1 over*)).tw
37.	((allocat* or allot* or assign* or divid*) adj3 (condition* or experiment* or intervention* or treatment* or therap* or control* or group*)).tw
38.	or/24-37
39.	9 and 13 and 23 and 38

Embase (via Elsevier)

1.	‘Patellofemoral Joint’:de
2.	‘Patella’:de
3.	‘Patellofemoral Pain Syndrome’:de
4.	‘Patella chondromalacia’:de
5.	‘anterior knee pain’:ab,ti,kw
6.	((patell* OR femoropatell* OR femoro-patell* OR retropatell*) NEAR/2 (pain OR syndrome OR dysfunction)):ab,ti,kw
7.	((chondromalac* OR chondropath* OR chondrosis OR arthralgia) NEAR/2 (knee* OR patell* OR femoropatell* OR femoro-patell* OR retropatell*)):ab,ti,kw
8.	((‘lateral compression’ OR ‘lateral facet’ OR ‘lateral pressure’ OR ‘odd facet’) NEAR/2 (syndrome OR pain)):ab,ti,kw
9.	or/1-8
10.	'Dynamometer':de
11.	'Muscle Strength':de
12.	(strength OR torque OR force OR moment* OR isokinetic OR isometric OR eccentric OR concentric OR power OR isotonic OR endurance OR kinetic* OR hand-held or dynamomet*):ti,ab,kw
13.	#10 OR #11 OR #12
14.	'pain':de
15.	'pain measurement':de
16.	'musculoskeletal pain':de
17.	'rehabilitation':de
18.	'convalescence':de
19.	'outcome assessment':de
20.	pain*:ab,ti OR discomfor*:ab,ti OR function*:ab,ti
21.	squat*:ab,ti OR jump*:ab,ti OR hop*:ab,ti OR drop*:ab,ti OR run*:ab,ti OR walk*:ab,ti OR stair:ab,ti OR step*:ab,ti OR cut*:ab,ti OR sit*:ab,ti OR stand*:ab,ti OR task:ab,ti OR performance:ab,ti
22.	scale:ab,ti,kw OR questionnaire:ab,ti,kw
23.	Or/14-22
24.	‘clinical trial (topic)’:de
25.	‘randomized controlled trial (topic)’:de
26.	‘controlled clinical trial (topic)’:de
27.	(randomi?ed OR randomly OR placebo OR sham):ab
28.	Trial:ti
29.	((clinical OR controlled OR comparative OR placebo OR sham OR prospective* OR randomi?ed) NEAR/3 (trial OR study)):ti,ab
30.	(random* NEAR/7 (allocat* OR allot* OR assign* OR basis* OR divid* OR order*)):ti,ab
31.	((singl* or doubl* or trebl* or tripl*) NEAR/7 (blind* or mask*)):ti,ab
32.	(cross?over* or (cross NEAR/1 over*)):ti,ab
33.	((allocat* or allot* or assign* or divid*) NEAR/3 (condition* or experiment* or intervention* or treatment* or therap* or control* or group*)):ti,ab
34.	Or/24-33
35.	9 and 13 and 23 and 34

CINAHL (via EBSCO)

1.	MH Patella
2.	MH Patellofemoral Pain Syndrome
3.	MH Chondromalacia Patella
4.	(anterior knee pain)
5.	((patell* OR femoropatell* OR femoro-patell* OR retropatell*) N2 (pain OR syndrome OR dysfunction))
6.	((chondromalac* OR chondropath* OR chondrosis or arthralgia) N2 (knee* OR patell* OR femoropatell* OR femoro-patell* OR retropatell*))
7.	((lateral compression OR lateral facet OR lateral pressure OR odd facet) N2 (syndrome OR pain))
8.	or/1-7
9.	MH Muscle Strength
10.	MH Exercise Test, Muscular
11.	(strength OR torque OR force OR moment* OR isokinetic OR isometric OR eccentric OR concentric OR power OR isotonic OR endurance OR kinetic* OR hand-held or dynamomet*)
12.	or/9-10
13.	MH Pain
14.	MH Pain Measurement
15.	MH Knee Pain
16.	MH Rehabilitation
17.	MH Functional Status
18.	MH Functional Assessment
19.	MH Patient-Reported Outcomes
20.	AB (pain* or discomfor* or function*)
21.	AB (squat* or jump* or hop* or drop* or run* or walk* or stair or step* or cut* or sit* or stand* or task or performance)
22.	(scale or questionnaire)
23.	Or/13-22
24.	MH Clinical Trials
25.	MH Randomized Controlled Trials
26.	PT Randomized controlled trial
27.	AB randomi?ed
28.	AB placebo
29.	AB sham
30.	AB randomly
31.	TI trial
32.	((clinical or controlled or comparative or placebo or prospective* or randomi?ed) N3 (trial or study))
33.	(random* N7 (allocat* or allot* or assign* or basis* or divid* or order*))
34.	((singl* or doubl* or trebl* or tripl*) N7 (blind* or mask*))
35.	(cross?over* or (cross N1 over*))
36.	((allocat* or allot* or assign* or divid*) N3 (condition* or experiment* or intervention* or treatment* or therap* or control* or group*))
37.	Or/24-36
38.	8 AND 12 AND 23 AND 37

SPORTDiscus (via EBSCO)

1.	SU PATELLOFEMORAL joint
2.	SU PATELLA
3.	SU PLICA syndrome
4.	SU PATELLOFEMORAL joint injuries
5.	SU CHONDROMALACIA patellae
6.	(anterior knee pain)
7.	((patell* OR femoropatell* OR femoro-patell* OR retropatell*) N2 (pain OR syndrome OR dysfunction))
8.	((chondromalac* OR chondropath* OR chondrosis OR arthralgia) N2 (knee* OR patell* OR femoropatell* OR femoro-patell* OR retropatell*))
9.	((lateral compression OR lateral facet OR lateral pressure OR odd facet) N2 (syndrome OR pain))
10.	OR/1-9
11.	SU MUSCLE strength measurement
12.	SU MUSCLE strength testing
13.	SU MUSCLE strength
14.	(strength OR torque OR force OR moment* OR isokinetic OR isometric OR eccentric OR concentric OR power OR isotonic OR endurance OR kinetic* OR hand-held or dynamomet*)
15.	or/11-14
16.	SU PAIN
17.	SU PAIN measurement
18.	SU KNEE pain
19.	SU REHABILITATION
20.	SU ACTIVITIES of daily living
21.	AB (pain* or discomfor* or function*)
22.	AB (squat* or jump* or hop* or drop* or run* or walk* or stair or step* or cut* or sit* or stand* or task or performance)
23.	TX (scale or questionnaire)
24.	Or/16-23
25.	AB Clinical
26.	AB randomi?ed
27.	AB placebo
28.	AB sham
29.	AB randomly
30.	TI trial
31.	TX ((clinical or controlled or comparative or placebo or prospective* or randomi?ed) N3 (trial or study))
32.	TX (random* N7 (allocat* or allot* or assign* or basis* or divid* or order*))
33.	TX ((singl* or doubl* or trebl* or tripl*) N7 (blind* or mask*))
34.	TX (cross?over* or (cross N1 over*))
35.	TX ((allocat* or allot* or assign* or divid*) N3 (condition* or experiment* or intervention* or treatment* or therap* or control* or group*))
36.	Or/25-35
37.	10 AND 15 AND 24 AND 36

Cochrane Library

1.	MeSH descriptor: [Patellofemoral Joint] this term only
2.	MeSH descriptor: [Patella] this term only
3.	MeSH descriptor: [Patellofemoral Pain Syndrome] this term only
4.	MeSH descriptor: [Chondromalacia Patellae] this term only
5.	(anterior knee pain)
6.	((patell* or femoropatell* or femoro-patell* or retropatell*) NEAR/2 (pain or syndrome or dysfunction))
7.	((chondromalac* or chondropath* or chondrosis) NEAR/2 (knee* or patell* or femoropatell* or femoro-patell* or retropatell*))
8.	((lateral compression or lateral facet or lateral pressure or odd facet) NEAR/2 (syndrome or pain))
9.	#1 OR #2 OR #3 OR #4 OR #5 OR #6 OR #7 OR #8
10.	MeSH descriptor: [Muscle Strength Dynamometer] this term only
11.	MeSH descriptor: [Muscle Strength] this term only
12.	(strength OR torque OR force OR moment* OR isokinetic OR isometric OR eccentric OR concentric OR power OR isotonic OR endurance OR kinetic* OR hand-held or dynamomet*)
13.	Or/10-12
14.	MeSH descriptor: [Pain] this term only
15.	MeSH descriptor: [Pain Measurement] this term only
16.	MeSH descriptor: [Musculoskeletal Pain] this term only
17.	MeSH descriptor: [Rehabilitation] this term only
18.	MeSH descriptor: [Recovery of Function] this term only
19.	MeSH descriptor: [Patient Outcome Assessment] this term only
20.	(pain* or discomfor* or function*):ti,ab,kw
21.	(squat* or jump* or hop* or drop* or run* or walk* or stair or step* or cut* or sit* or stand* or task or performance):ab,ti,kw
22.	(scale or questionnaire)
23.	Or/14-22
24.	9 AND 13 AND 23
25.	Limit 24 to Trials

Supplementary Material C. Correlation coefficients used for covariance calculations.

- ***Muscle Strength x Pain***

Knee Extension – r = -0.16

Source: Meta-analysis using data from nine studies.
References: Amin et al., 2019; Bily et al., 2008; Esculier et al., 2016; Hott et al., 2022; Kim and Park, 2022; Manojlovic et al., 2022; Piva et al., 2009; Powers et al., 2004; Sutter et al., 1998.

Knee Flexion – r = -0.41

Source: Meta-analysis using data from three studies.
References: Amin et al., 2019; Ducatti et al., 2021; Manojlovic et al., 2022.

Hip Abduction – r = 0.01

Source: Meta-analysis using data from six studies.
References: Almeida et al., 2015; Amin et al., 2019; Esculier et al., 2016; Long-Rossi and Salsich, 2010; Manojlovic et al., 2022; Piva et al., 2009.

Hip Adduction – r = 0.02

Source: Data from Manojlovic et al., 2022.
Reference: Manojlovic et al., 2022.

Hip Extension – r = -0.16

Source: Meta-analysis using data from four studies.
References: Almeida et al., 2015; Esculier et al., 2016; Long-Rossi and Salsich, 2010; Manojlovic et al., 2022.

Hip Internal Rotation – r = -0.59

Source: Data from Manojlovic et al., 2022.
Reference: Manojlovic et al., 2022.

Hip External Rotation – r = -0.08

Source: Meta-analysis using data from six studies.
References: Almeida et al., 2015; Amin et al., 2019; Esculier et al., 2016; Long-Rossi and Salsich, 2010; Manojlovic et al., 2022; Piva et al., 2009.

- ***Muscle Strength x Function***

Knee Extension – r = 0.20

Source: Meta-analysis using data from ten studies.
References: Amin et al., 2019; Bily et al., 2008; Briani et al., 2021; Coskun et al., 2024; Esculier et al., 2016; Glaviano and Saliba, 2017; Hott et al., 2022; Kim and Park, 2022; Piva et al., 2009; Powers et al., 2004.

Knee Flexion – r = 0.12

Source: Meta-analysis using data from three studies.
References: Amin et al., 2019; Briani et al., 2021; Coskun et al., 2024.

Hip Abduction – r = 0.18

Source: Meta-analysis using data from six studies.

References: Almeida et al., 2015; Amin et al., 2019; Esculier et al., 2016; Glaviano and Saliba, 2017; Long-Rossi and Salsich, 2010; Piva et al., 2009.

Hip Adduction – r = 0.41

Source: Data from Glaviano and Saliba, 2017.

References: Glaviano and Saliba, 2017.

Hip Extension – r = 0.35

Source: Meta-analysis using data from four studies.

References: Almeida et al., 2015; Esculier et al., 2016; Glaviano and Saliba, 2017; Long-Rossi and Salsich, 2010.

Hip Internal Rotation – r = 0.40

Arbitrarily decided considering the lack of information.

Hip External Rotation – r = 0.30

Source: Meta-analysis using data from six studies.

References: Almeida et al., 2015; Amin et al., 2019; Esculier et al., 2016; Glaviano and Saliba, 2017; Long-Rossi and Salsich, 2010; Piva et al., 2009.

Supplementary Material D. Excluded studies after full-text screening with reasons (n=129).

ABDALLAH, D; BEHIRY, M. "The Effect Of Sensory Motor Training in Treatment of Patellofemoral Pain Syndrome". https://trialsearch.who.int/Trial2.aspx?TrialID=PACTR202301535060049. John Wiley & Sons, Ltd., 2023. https://www.cochranelibrary.com/central/doi/10.1002/central/CN-02521635/full.
Reason for exclusion: *RCT Protocol*

AGHAKESHIZADEH, F; LETAFATKAR, A. "Comparison of the Effect of Hip and Knee Strengthening with Internal Instruction Exercises on Pain and Dynamic Knee Valgus in Patients with Patellofemoral Pain Syndrome". Journal of Research in Rehabilitation Sciences 15, nº 2 (2020). https://doi.org/10.22122/jrrs.v15i2.3408.
Reason for exclusion: *No strength assessment*

AGHAPOUR, E; FAHIMEH, K; EHSAN, S. "Effects of Kinesio Taping® on Knee Function and Pain in Athletes with Patellofemoral Pain Syndrome". Journal of Bodywork and Movement Therapies 21, nº 4 (2017): 835–39. https://doi.org/10.1016/j.jbmt.2017.01.012.
Reason for exclusion: *Non-Randomized Clinical Trial*

AKBARI, A. "A Comparison between Open with Closed Kinematic Chain Exercises of Knee on Decreasing Pain and Improving Function in Subjects with Anterior Knee Pain". https://trialsearch.who.int/Trial2.aspx?TrialID=IRCT201101201675N5. John Wiley & Sons, Ltd., 2019. https://www.cochranelibrary.com/central/doi/10.1002/central/CN-01868540/full.
Reason for exclusion: *RCT Protocol*

ALI, A. "Effect of Short Foot Exercise in Treatment of Patients With Patellofemoral Pain Syndrome". https://clinicaltrials.gov/show/NCT05383781. John Wiley & Sons, Ltd., 2022. https://www.cochranelibrary.com/central/doi/10.1002/central/CN-02399976/full.
Reason for exclusion: *RCT Protocol*

ALMEIDA, G. "Effect of Two Strengthening Protocols for Lower Limbs in Patients With Patellofemoral Pain". https://clinicaltrials.gov/show/NCT03163290. John Wiley & Sons, Ltd., d2018. https://www.cochranelibrary.com/central/doi/10.1002/central/CN-01494393/full.
Reason for exclusion: *RCT Protocol*

AMJAD, I. "Comparison of Open and Closed Chain Exercises in Athletes With Patellofemoral Pain Syndrome". https://www.cochranelibrary.com/central/doi/10.1002/central/CN-02519519/full.
Reason for exclusion: *RCT Protocol*

ARGUT, S.K, et al. "The Effectiveness Of Quadriceps Strengthening Exercises Combined With Neuromuscular Electrical Stimulation on Patellofemoral Pain Syndrome". Orthopaedic Journal of Sports Medicine 5, no 2_suppl2 (2017): 2325967117S0010. https://doi.org/10.1177/2325967117S00107.
Reason for exclusion: *Congress abstract*

ASIF, M. “Comparison of Hip Strengthening Exercises and Knee Exercises on Pain, Muscle Strength and Function in Runners with Knee Pain”. https://trialsearch.who.int/Trial2.aspx?TrialID=IRCT20210330050789N1. John Wiley & Sons, Ltd., 2021. https://www.cochranelibrary.com/central/doi/10.1002/central/CN-02329668/full.
Reason for exclusion: *RCT Protocol*

BAKHTIARY, A H; FATEMI, E. “Open versus Closed Kinetic Chain Exercises for Patellar Chondromalacia”. British Journal of Sports Medicine 42, no 2 (2007): 99–102. https://doi.org/10.1136/bjsm.2007.038109.
Reason for exclusion: *Non-inclusion of patellofemoral pain*

BALCI, P. et al. “The Effects of Two Different Closed Kinetic Chain Exercises on Muscle Strength and Proprioception in Patients with Patellofemoral Pain Syndrome”. Acta Orthopaedica et Traumatologica Turcica 43, no 5 (2009): 419–25. https://doi.org/10.3944/AOTT.2009.419.
Reason for exclusion: *No strength assessment*

BALDON, R. et al. “Evaluating Eccentric Hip Torque and Trunk Endurance as Mediators of Changes in Lower Limb and Trunk Kinematics in Response to Functional Stabilization Training in Women With Patellofemoral Pain”. The American Journal of Sports Medicine 43, no 6 (2015): 1485–93. https://doi.org/10.1177/0363546515574690.
Reason for exclusion: *Non-Randomized Clinical Trial*

BARTON, C. et al. “A Proximal Progressive Resistance Training Program Targeting Strength and Power Is Feasible in People with Patellofemoral Pain”. Physical Therapy in Sport 38 (2019): 59–65. https://doi.org/10.1016/j.ptsp.2019.04.010.
Reason for exclusion: *Non-Randomized Clinical Trial*

BAZETT-JONES, D. “Acute Responses of Strength and Running Mechanics to Increasing and Decreasing Pain in Patients With Patellofemoral Pain”. Journal of Athletic Training 52, no 5 (2017): 411–21. https://doi.org/10.4085/1062-6050-53.3.04.
Reason for exclusion: *Non-Randomized Clinical Trial*

BLOOMER, B.A; DURALL, C.J. “Does the Addition of Hip Strengthening to a Knee-Focused Exercise Program Improve Outcomes in Patients With Patellofemoral Pain Syndrome?” Journal of Sport Rehabilitation 24, no 4 (2015): 428–33. https://doi.org/10.1123/jsr.2014-0184.
Reason for exclusion: *Non-Randomized Clinical Trial*

BOITRAGO, M. “Effects of Strength Exercises for Anterior Knee Pain”. https://trialsearch.who.int/Trial2.aspx?TrialID=RBR-53rgg2. John Wiley & Sons, Ltd., 2019. https://www.cochranelibrary.com/central/doi/10.1002/central/CN-01947285/full.
Reason for exclusion: *RCT Protocol*

BOLGLA, L.A. et al. “Pain, function, and strength outcomes for males and females with patellofemoral pain who participate in either a hip/core- or knee-based rehabilitation program”. *International Journal of Sports Physical Therapy* 11, nº 6 (2016): 926–35.
Reason for exclusion: *Secondary analysis*

CHANG, N.J. “Comparison of Knee Strengthening Combined With Hip or Ankle Joint Exercises on Clinical Symptoms, Sports Performance and Soft Tissue Structure in Women With Patellofemoral Pain Syndrome”. https://clinicaltrials.gov/study/NCT07210060
Reason for exclusion: *RCT Protocol*

CHRISTOU, E.A. “Patellar Taping Increases Vastus Medialis Oblique Activity in the Presence of Patellofemoral Pain”. Journal of Electromyography and Kinesiology 14, no 4 (2004): 495–504. https://doi.org/10.1016/j.jelekin.2003.10.007.
Reason for exclusion: *No strength assessment*

COWAN, S.M. et al. “Altered Hip and Trunk Muscle Function in Individuals with Patellofemoral Pain”. British Journal of Sports Medicine 43, nº 8 (2009): 584–88. https://doi.org/10.1136/bjsm.2008.053553.
Reason for exclusion: *Non-Randomized Clinical Trial*

ÇUBUKÇU, D. et al. “Efficacy of open versus closed kinetic chain exercises for patients with patellofemoral pain syndrome”. Journal of Rheumatology and Medical Rehabilitation 15 (2004): 16–25.
Reason for exclusion: *Non-English paper*

DE ALBUQUERQUE, T.A.B. et al. “Correlation of Pain Sensitization with Muscle Strength and Angular Kinematics in Women with Patellofemoral Pain”. Clinical Biomechanics 81 (2021): 105217. https://doi.org/10.1016/j.clinbiomech.2020.105217.
Reason for exclusion: *Non-Randomized Clinical Trial*

DEMIRTAŞ, C. “Effectiveness Of Core Stabilization Exercises In Patellofemoral Pain Syndrome”. https://clinicaltrials.gov/show/NCT05351814. John Wiley & Sons, Ltd., 2022. https://www.cochranelibrary.com/central/doi/10.1002/central/CN-02404839/full.
Reason for exclusion: *RCT Protocol*

DENG, F. et al. “Tibial Internal and External Rotation Taping for Improving Pain in Patients with Patellofemoral Pain Syndrome”. Journal of Science and Medicine in Sport 25, no 8 (2022): 644–48. https://doi.org/10.1016/j.jsams.2022.04.003.
Reason for exclusion: *No strength assessment*

DIAS, I. “Deep Transverse Massage as a Potentiating Resource in the Treatment of Patellofemoral Pain Syndrome”. https://www.cochranelibrary.com/central/doi/10.1002/central/CN-02412885/full.
Reason for exclusion: *RCT Protocol*

DIMITRIOS, S. et al. “Comparing the Effects of Eccentric Training with Eccentric Training and Static Stretching Exercises in the Treatment of Patellar Tendinopathy. A Controlled Clinical Trial”. Clinical Rehabilitation 26, no 5 (2012): 423–30. https://doi.org/10.1177/0269215511411114.
Reason for exclusion: *Non-inclusion of patellofemoral pain*

DOLAK, K.L. “Hip-Strengthening Exercises Before Functional Exercises Reduced Pain in Women with Patellofemoral Pain Syndrome”. Journal of Bone and Joint Surgery 94 (2012): 940–940. https://doi.org/10.2106/JBJS.9410.ebo274.
Reason for exclusion: *Comment on article*

EARL-BOEHM, J.E. et al. “Treatment Success of Hip and Core or Knee Strengthening for Patellofemoral Pain: Development of Clinical Prediction Rules”. Journal of Athletic Training 53, no 6 (2018): 545–52. https://doi.org/10.4085/1062-6050-510-16.
Reason for exclusion: *Secondary analysis*

EBURNE, J.; BANNISTER, G. “The McConnell Regimen versus Isometric Quadriceps Exercises in the Management of Anterior Knee Pain. A Randomised Prospective Controlled Trial”. The Knee 3, no 3 (1996): 151–53. https://doi.org/10.1016/0968-0160(96)00214-1.
Reason for exclusion: *No strength assessment*

ESCULIER, J.F. et al. “The Effects of a Multimodal Rehabilitation Program on Symptoms and Ground-Reaction Forces in Runners With Patellofemoral Pain Syndrome”. Journal of Sport Rehabilitation 25, no 1 (2016): 23–30. https://doi.org/10.1123/jsr.2014-0245.
Reason for exclusion: *Non-Randomized Clinical Trial*

ESFANDIARPOUR, F. “Comparison of the Effects of Knee and Hip Muscle Exercises and Isolated Knee Muscle Exercises in Treatment of Anterior Knee Pain”. https://trialsearch.who.int/Trial2.aspx?TrialID=IRCT2014070518362N1. John Wiley & Sons, Ltd., 2019. https://www.cochranelibrary.com/central/doi/10.1002/central/CN-01859086/full.
Reason for exclusion: *RCT Protocol*

ESFANDIARPOUR, F.; KESHAVARZ, M. “Effects of Muscle Strengthening on Motion Characteristics in Patellofemoral Pain”. https://trialsearch.who.int/Trial2.aspx?TrialID=IRCT20140705018362N4. John Wiley & Sons, Ltd., 2019. https://www.cochranelibrary.com/central/doi/10.1002/central/CN-01947447/full.
Reason for exclusion: *RCT Protocol*

ESPÍ-LÓPEZ, G. et al. Effectiveness of Inclusion of Dry Needling in a Multimodal Therapy Program for Patellofemoral Pain: A Randomized Parallel-Group Trial. J Orthop Sports Phys Ther. 2017 Jun;47(6):392-401. doi: 10.2519/jospt.2017.7389.
Reason for exclusion: *No clinical measures*

FELICIO, L. “Benefits of functional training in women with knee pain”. https://www.cochranelibrary.com/central/doi/10.1002/central/CN-01894895/full.
Reason for exclusion: *RCT Protocol*

FERBER, R. et al. “Changes in Knee Biomechanics After a Hip-Abductor Strengthening Protocol for Runners With Patellofemoral Pain Syndrome”. Journal of Athletic Training 46, no 2 (2011): 142–49. https://doi.org/10.4085/1062-6050-46.2.142.
Reason for exclusion: *Non-Randomized Clinical Trial*

FOROUGHI, F.; MOTEALLEH, A. “The Effect of Core Training Using Unstable Sitting in Patients with Patellofemoral Pain Syndrome”. https://trialsearch.who.int/Trial2.aspx?TrialID=IRCT2016120415932N12. John Wiley & Sons, Ltd., 2019. https://www.cochranelibrary.com/central/doi/10.1002/central/CN-01853468/full.
Reason for exclusion: *RCT Protocol*

FOROUGHI, F. et al. “Added Value of Isolated Core Postural Control Training on Knee Pain and Function in Women With Patellofemoral Pain Syndrome: A Randomized Controlled Trial”. Archives of Physical Medicine and Rehabilitation 100, no 2 (2019): 220–29. https://doi.org/10.1016/j.apmr.2018.08.180.
Reason for exclusion: *No strength assessment*

FUKUDA, T.Y. et al. “Hip Posterolateral Musculature Strengthening in Sedentary Women With Patellofemoral Pain Syndrome: A Randomized Controlled Clinical Trial With 1-Year Follow-Up”. Journal of Orthopaedic & Sports Physical Therapy 42, no 10 (2012): 823–30. https://doi.org/10.2519/jospt.2012.4184.
Reason for exclusion: *No strength assessment*

FUKUDA, T.Y. et al. “Short-Term Effects of Hip Abductors and Lateral Rotators Strengthening in Females With Patellofemoral Pain Syndrome: A Randomized Controlled Clinical Trial”. Journal of Orthopaedic & Sports Physical Therapy 40, no 11 (2010): 736–42. https://doi.org/10.2519/jospt.2010.3246.
Reason for exclusion: *No strength assessment*

GOYAL, M. “The Effect of Hip Bone Mobilization in Patients with Anterior Knee Pain”. http://www.who.int/trialsearch/Trial2.aspx?TrialID=CTRI/2018/10/015929. John Wiley & Sons, Ltd., 2022. https://www.cochranelibrary.com/central/doi/10.1002/central/CN-02433758/full.
Reason for exclusion: *RCT Protocol*

GREAVES, H. et al. “How Effective Is an Evidence-Based Exercise Intervention in Individuals with Patellofemoral Pain?” Physical Therapy in Sport 51 (2021): 92–101. https://doi.org/10.1016/j.ptsp.2021.05.013.
Reason for exclusion: *Non-Randomized Clinical Trial*

GRINDSTAFF, T.L. et al. “Lumbopelvic Joint Manipulation and Quadriceps Activation of People With Patellofemoral Pain Syndrome”. Journal of Athletic Training 47, no 1 (2012): 24–31. https://doi.org/10.4085/1062-6050-47.1.24.
Reason for exclusion: *No clinical measures*

GÜNEY, H. et al. “Effects of Different Isokinetic Training Programs on Hamstring/Quadriceps Ratio and Proprioception in Patients with Patellofemoral Pain”. Em Orthopaedic Journal of Sports Medicine, 2:1–1. Thousand Oaks, California: Sage Publications Inc., 2014. https://doi.org/10.1177/2325967114S00147.
Reason for exclusion: *Congress abstract*

GÜNEY, H. et al. “The Relationship between Quadriceps Strength and Joint Position Sense, Functional Outcome and Painful Activities in Patellofemoral Pain Syndrome”. Knee Surgery, Sports Traumatology, Arthroscopy 24, no 9 (2016): 2966–72. https://doi.org/10.1007/s00167-015-3599-3.
Reason for exclusion: *Non-Randomized Clinical Trial*

GURUDUT, P.; DINIZ, W. “Effects of the Traning Octagon Programme in Anterior Knee Pain”. https://trialsearch.who.int/Trial2.aspx?TrialID=CTRI/2022/09/045634. John Wiley & Sons, Ltd., 2022. https://www.cochranelibrary.com/central/doi/10.1002/central/CN-02473277/full.

Reason for exclusion: *RCT Protocol*

HADADNEZHAD, M.; LETAFATKAR, A. "The Effect of Eccentric Exercises with Gait Retraining on Reducing Patellofemoral Pain". https://trialsearch.who.int/Trial2.aspx?TrialID=IRCT20220812055662N1. John Wiley & Sons, Ltd., 2022. https://www.cochranelibrary.com/central/doi/10.1002/central/CN-02499141/full.
Reason for exclusion: *RCT Protocol*

HAFEZ, A.R. et al. "Eccentric versus Concentric Contraction of Quadriceps Muscles in Treatment of Chondromalacia Patellae". World Journal of Medical Sciences 7(3):197-203, 2012.
Reason for exclusion: *No strength assessment*

HALL, S. "The Effect of Stretching and Transverse Friction Massage to Gastrocnemius for Patellofemoral Pain Syndrome". http://www.who.int/trialsearch/Trial2.aspx?TrialID=ISRCTN56283223. John Wiley & Sons, Ltd., 2022. https://www.cochranelibrary.com/central/doi/10.1002/central/CN-02444549/full.
Reason for exclusion: *RCT Protocol*

HAMSTRA-WRIGHT, K. et al. Patient-Reported Outcomes Remain Improved 6-Months Post Patellofemoral Pain Rehabilitation. Journal of Sport Rehabilitation. 26. 10.1123/jsr.2015-0176.
Reason for exclusion: *Secondary analysis*

HANDFIELD, T.; KRAMER, J. "Effect of McConnell taping on perceived pain and knee extensor torques during isokinetic exercise performed by patients with patellofemoral pain syndrome". Physiotherapy Canada 52, no 1 (2000): 39–44.
Reason for exclusion: *Unavailable*

HART, J.M. et al. "Prospective, Randomized, Double Blind Evaluation of the Efficacy of a Single Dose Hyaluronic Acid for the Treatment of Patellofemoral Chondromalacia". Orthopaedic Journal of Sports Medicine 6, no 7_suppl4 (2018): 2325967118S0011. https://doi.org/10.1177/2325967118S00118.
Reason for exclusion: *Pharmacological treatment*

HASTANESI, T. "Evaluation of the Results of Closed Kinetic Chain Exercises in Patellofemoral Pain Syndrome". https://clinicaltrials.gov/show/NCT05427357. John Wiley & Sons, Ltd., 2022. https://www.cochranelibrary.com/central/doi/10.1002/central/CN-02413161/full.
Reason for exclusion: *RCT Protocol*

HAZNECI, B. et al. "Efficacy of Isokinetic Exercise on Joint Position Sense and Muscle Strength in Patellofemoral Pain Syndrome". American Journal of Physical Medicine & Rehabilitation 84, no 7 (2005): 521–27. https://doi.org/10.1097/01.phm.0000167682.58210.a7.
Reason for exclusion: *Non-Randomized Clinical Trial*

HERRINGTON, L.; AL-SHEHRI, A.S. “Comparison of single and multiple joint quadriceps exercise in anterior knee pain rehabilitation”. Em Physical Therapy in Sport, 7:171–72, 2006. https://doi.org/10.1016/j.ptsp.2006.09.003.
Reason for exclusion: *Congress abstract*

HERRINGTON, L. “The Effect of Patellar Taping on Quadriceps Peak Torque and Perceived Pain: A Preliminary Study”. Physical Therapy in Sport 2, no 1 (2001): 23–28. https://doi.org/10.1054/ptsp.2000.0034.
Reason for exclusion: *Non-Randomized Clinical Trial*

HOLDEN, S. et al. “How Do Hip Exercises Improve Pain in Individuals With Patellofemoral Pain? Secondary Mediation Analysis of Strength and Psychological Factors as Mechanisms”. Journal of Orthopaedic & Sports Physical Therapy 51, no 12 (2021): 602–10. https://doi.org/10.2519/jospt.2021.10674.
Reason for exclusion: *Secondary analysis*

HONARPISHEH, R. “Effect of Quadriceps Muscle Strengthening Exercises on the Fiber Angle Ratio of Vastus Medialis Obliquus to Vastus Lateralis in Patient Wit Patellofemoral Pain Syndrome”. https://trialsearch.who.int/Trial2.aspx?TrialID=IRCT2014093019346N1. John Wiley & Sons, Ltd., 2019. https://www.cochranelibrary.com/central/doi/10.1002/central/CN-01883111/full.
Reason for exclusion: *RCT Protocol*

HONG, Q. “The Effect of Exercise Intervention Programs on Patient with Patellofemoral Joint Pain Syndrome”. https://trialsearch.who.int/Trial2.aspx?TrialID=ChiCTR2200056224. John Wiley & Sons, Ltd., 2023. https://www.cochranelibrary.com/central/doi/10.1002/central/CN-02544390/full.
Reason for exclusion: *RCT Protocol*

HOTT, A. et al. Patellofemoral Pain: One Year Results of a Randomized Trial Comparing Hip Exercise, Knee Exercise, or Free Activity”. Scandinavian Journal of Medicine & Science in Sports 30, no 4 (2020): 741–53. https://doi.org/10.1111/sms.13613.
Reason for exclusion: *Follow-up data*

HU, H. et al. “Effects of Neuromuscular Training on Pain Intensity and Self-Reported Functionality for Patellofemoral Pain Syndrome in Runners: Study Protocol for a Randomized Controlled Clinical Trial”. Trials, 2019. https://trialsjournal.biomedcentral.com/articles/10.1186/s13063-019-3503-4.
Reason for exclusion: *RCT Protocol*

JAMALI, N. “Effect of Hip Muscles Strengthening Exercises on Anterior Knee Pain”. https://trialsearch.who.int/Trial2.aspx?TrialID=IRCT20190816044538N1. John Wiley & Sons, Ltd., 2020. https://www.cochranelibrary.com/central/doi/10.1002/central/CN-02069694/full.
Reason for exclusion: *RCT Protocol*

JAN, M.H. “Effects of Hip Abductor and External Rotator Strength Training in Patients With Patellofemoral Pain Syndrome”. https://clinicaltrials.gov/show/NCT00736736. John

Wiley & Sons, Ltd., 2018. https://www.cochranelibrary.com/central/doi/10.1002/central/CN-01520255/full.
Reason for exclusion: *RCT Protocol*

JAVED, H. “Effects of Core Stability Exercises in Patients With Patellofemoral Pain Syndrome”. https://www.cochranelibrary.com/central/doi/10.1002/central/CN-02382055/full.
Reason for exclusion: *RCT Protocol*

KALANTARI, K.K.; MIKAILI, S. “The Effect of Strengthening Exercises on Anterior Knee Pain Syndrome”. https://trialsearch.who.int/Trial2.aspx?TrialID=IRCT20200825048523N2. John Wiley & Sons, Ltd., 2023. https://www.cochranelibrary.com/central/doi/10.1002/central/CN-02521063/full.
Reason for exclusion: *RCT Protocol*

KANNUS, P. et al. “An Outcome Study of Chronic Patellofemoral Pain Syndrome. Seven-Year Follow-up of Patients in a Randomized, Controlled Trial”. The Journal of Bone and Joint Surgery. American Volume 81, no 3 (1999): 355–63. https://doi.org/10.2106/00004623-199903000-00007.
Reason for exclusion: *Pharmacological treatment*

KARAKUS, D. et al. “Patellofemoral Pain Syndrome: Results of a Home Exercise Program Versus Isokinetic Exercise Program on Functional Capacity”. Türkiye Fiziksel Tip ve Rehabilitasyon Dergisi 60, no 1 (2014): 63–67. https://doi.org/10.5152/tftrd.2014.93723.
Reason for exclusion: *Congress abstract*

KEAYS, S.L. et al. “Individualized Physiotherapy in the Treatment of Patellofemoral Pain: Individualized Physiotherapy for Patellofemoral Pain”. Physiotherapy Research International 20, no 1 (2015): 22–36. https://doi.org/10.1002/pri.1593.
Reason for exclusion: *Non-Randomized Clinical Trial*

KEAYS, S.L. et al. “Three-Year Outcome After a 1-Month Physiotherapy Program of Local and Individualized Global Treatment for Patellofemoral Pain Followed by Self-Management”. Clinical Journal of Sport Medicine 26, no 3 (2016): 190–98. https://doi.org/10.1097/JSM.0000000000000226.
Reason for exclusion: *Non-Randomized Clinical Trial*

KESHMARZI, S.K. et al. “The effects of six weeks of Core stabilization exercise on pain, Functional disability and Isometric strength of the trunk and lower extremities Muscle in women with patellofemoral pain syndrome. (English)”. Advances in Nursing & Midwifery 27, no 1 (2018): 24–32.
Reason for exclusion: *Non-English paper*

KHAYAMBASHI, K. et al. “Posterolateral Hip Muscle Strengthening Versus Quadriceps Strengthening for Patellofemoral Pain: A Comparative Control Trial”. Archives of Physical Medicine and Rehabilitation 95, no 5 (2014): 900–907. https://doi.org/10.1016/j.apmr.2013.12.022.
Reason for exclusion: *No strength assessment*

KISACIK, P. et al. “Effects of short foot exercises on fore-and midfoot characteristics and strength of flexor hallucis longus in patients with patellofemoral pain”, 29:249. 2. Turkey, 2018. https://doi.org/10.1002/central/CN-01925300.
Reason for exclusion: *Congress abstract*

KUMAR. A. et al. “Comparative Analysis of Swiss Ball and Foam Roller usage with SLR Technique for Hip Muscle Strengthening of Female Athletes with Patellofemoral Pain Syndrome.” Biomedicine 38 (2018): 219–24.
Reason for exclusion: *No strength assessment*

KUMAR, T. et al. “Kinetic Chain Exercise For Patello Femoral Pain Syndrome - A Randomised Control Study”. Indian Journal of Physiotherapy and Occupational Therapy - An International Journal 7 (2013): 245. https://doi.org/10.5958/j.0973-5674.7.3.101.
Reason for exclusion: *No strength assessment*

KURU, T. et al. “Comparison of Efficiency of Kinesio® Taping and Electrical Stimulation in Patients with Patellofemoral Pain Syndrome”. Acta Orthopaedica et Traumatologica Turcica 46, no 5 (2012): 385–92. https://doi.org/10.3944/AOTT.2012.2682.
Reason for exclusion: *No continuous data*

LACK, S. et al. “The Immediate Effects of Foot Orthoses on Hip and Knee Kinematics and Muscle Activity during a Functional Step-up Task in Individuals with Patellofemoral Pain”. Clinical Biomechanics 29, no 9 (2014): 1056–62. https://doi.org/10.1016/j.clinbiomech.2014.08.005.
Reason for exclusion: *No strength assessment*

LEE, J. et al. “Prospective comparative study between knee alignment-oriented static and dynamic balance exercise in patellofemoral pain syndrome patients with dynamic knee valgus”. Medicine (Baltimore). 2022; 101(37):e30631. doi:10.1097/MD.0000000000030631
Reason for exclusion: *Non-Randomized Clinical Trial*

LETAFATKAR, A. “Effect of Education on Improving Patellofemoral Pain”. https://trialsearch.who.int/Trial2.aspx?TrialID=IRCT20210701051754N2. John Wiley & Sons, Ltd., 2021. https://www.cochranelibrary.com/central/doi/10.1002/central/CN-02329841/full.
Reason for exclusion: *RCT Protocol*

LOUDON, J.K. et al. “The Effectiveness of Exercise in Treating Patellofemoral-Pain Syndrome”. Journal of Sport Rehabilitation 13, no 4 (2004): 323–42. https://doi.org/10.1123/jsr.13.4.323.
Reason for exclusion: *No strength assessment*

LUCARELLI, P. “Kinematics of Lower Limb, Pain and Function of the Women With Patellofemoral Pain Syndrome”. https://clinicaltrials.gov/study/NCT01804608
Reason for exclusion: *RCT Protocol*

LUCARELI, P. “The Effect of Muscle Strengthening Associated With Neuromuscular Stimulus in Patients With Patellofemoral Pain During Negotiating Stairs”.

https://clinicaltrials.gov/show/NCT02591680. John Wiley & Sons, Ltd., 2018. https://www.cochranelibrary.com/central/doi/10.1002/central/CN-01493385/full.
Reason for exclusion: *RCT Protocol*

MAGALHÃES, E. et al. "A Comparison of Hip Strength Between Sedentary Females With and Without Patellofemoral Pain Syndrome". Journal of Orthopaedic & Sports Physical Therapy 40, no 10 (2010): 641–47. https://doi.org/10.2519/jospt.2010.3120.
Reason for exclusion: *Non-Randomized Clinical Trial*

MAZAHERINEZHAD, A. "Comparison of Two Methods of Exercise Therapy in Patellofemoral Pain Syndrome". https://trialsearch.who.int/Trial2.aspx?TrialID=IRCT2014090719073N1. John Wiley & Sons, Ltd., 2019. https://www.cochranelibrary.com/central/doi/10.1002/central/CN-01867684/full.
Reason for exclusion: *RCT Protocol*

MCMULLEN, W. et al. "Static and Isokinetic Treatments of Chondromalacia Patella: A Comparative Investigation". Journal of Orthopaedic & Sports Physical Therapy 12, no 6 (1990): 256–66. https://doi.org/10.2519/jospt.1990.12.6.256.
Reason for exclusion: *Non-inclusion of patellofemoral pain*

MIANUTTI, G.O. et al. "Analysis of Postural Control and Core Muscle Endurance in Female Runners with Anterior Knee Pain". Em Physical Therapy in Sport, 47:e1–2, 2021. https://doi.org/10.1016/j.ptsp.2020.11.004.
Reason for exclusion: *Congress abstract*

MOTEALLEH, A.; ASHAN, E.A. "Comparison the Effect of Trunk Training with Hip and Knee Strength Training on Pain, Function and Proprioception in Patients with Patellofemoral Pain". https://trialsearch.who.int/Trial2.aspx?TrialID=IRCT20131225015932N16. John Wiley & Sons, Ltd., 2020. https://www.cochranelibrary.com/central/doi/10.1002/central/CN-02170166/full.
Reason for exclusion: *RCT Protocol*

MOUSAVI, S.M. et al. "The Effects of Kinesiotape and Strength Training on Knee Pain and Quadriceps Strength in People with Patellofemoral Pain Syndrome (PFPS)". Journal of Isfahan Medical School 29, no 159 (2011): 1–12.
Reason for exclusion: *Non-English paper*

MUSTAFA, M. et al. "Comparison between the Effect of Closed Kinetic Chain and Open Kinetic Chain Exercises in the Strengthening of Vastus Medialis Obliqus in Subjects with Patello-Femoral Pain Syndrome - a Randomized Control Trial". Pakistan Journal of Medical and Health Sciences 16, no 6 (2022): 185–86. https://doi.org/10.53350/pjmhs22166185.
Reason for exclusion: *No strength assessment*

NEJATI, N.; MOTEALLEH, A. "The Effects of Trunk Stabilization Exercise on Anterior Knee Pain". https://trialsearch.who.int/Trial2.aspx?TrialID=IRCT2014021315932N2. John Wiley & Sons, Ltd., 2019. https://www.cochranelibrary.com/central/doi/10.1002/central/CN-01831057/full.

Reason for exclusion: *RCT Protocol*

OLIVEIRA, N. "Dose-Response Effect of Strength Training on the Rehabilitation of Patients with Patellofemoral Pain Syndrome". https://trialsearch.who.int/Trial2.aspx?TrialID=RBR-8648fg. John Wiley & Sons, Ltd., 2020. https://www.cochranelibrary.com/central/doi/10.1002/central/CN-02173898/full.
Reason for exclusion: *RCT Protocol*

OSORIO, J.A. et al. "The Effects of Two Therapeutic Patellofemoral Taping Techniques on Strength, Endurance, and Pain Responses". Physical Therapy in Sport 14, no 4 (2013): 199–206. https://doi.org/10.1016/j.ptsp.2012.09.006.
Reason for exclusion: *Non-Randomized Clinical Trial*

OWINGS, T.M.; GRABINER, M.D. "Differences in braced and nonbraced maximum concentric and eccentric knee extension strength in subjects with patellofemoral joint pain". International Society of Biomechanics. Congress (16th: 1997: Tokyo, Japan). SPHS-42579
Reason for exclusion: *Congress abstract*

ÖZDEMIR, Y. "Comparing the Effectiveness of Kinesio Taping and Knee Orthosis in Patients with Patellofemoral Pain Syndrome". https://clinicaltrials.gov/ct2/show/NCT05995210
Reason for exclusion: *RCT Protocol*

PARTOVI, G. et al. "Effect of Taping and Quadriceps Strengthening and Hamstring Stretching on Patello-Femoral Pain Syndrome: A Randomized Clinical Trial". https://doi.org/10.1002/central/CN-02286938.
Reason for exclusion: *RCT Protocol*

PATIL, K.; PATIL, P. "Effect of Patellar Realignment Training in Patellofemoral Pain Syndrome". Indian Journal of Public Health Research & Development 14, no 1 (2023): 136–41. https://doi.org/10.37506/ijphrd.v14i1.18811.
Reason for exclusion: *No continuous data*

PATLE, S.; BHAVE, S. "A Study on the Efficacy of Manual Therapy as an Intervention to Supervised Exercise Therapy in Patients with Anterior Knee Pain: a Randomised Controlled Trial". Indian Journal of Physiotherapy and Occupational Therapy 9 (2015) 92-96. DOI:10.5958/0973-5674.2015.00060.X
Reason for exclusion: *No clinical measures*

PATRA, R.; SHARMA, M. "Brain electrical stimulation in combination with exercise for abdomen, back, hip, knee muscles in management of Knee pain syndrome | Cochrane Library". https://www.cochranelibrary.com/central/doi/10.1002/central/CN-02349577/full.
Reason for exclusion: *RCT Protocol*

PEARCE, T.L. "Eccentric Drop Squats as a Means of Conservative Treatment for Patellofemoral Pain Syndrome". University of British Columbia, 2003. https://open.library.ubc.ca/soa/cIRcle/collections/ubctheses/831/items/1.0077294.
Reason for exclusion: *Dissertation*

PEELER, J.; ANDERSON, J.E. "Effectiveness of Static Quadriceps Stretching in Individuals With Patellofemoral Joint Pain". Clinical Journal of Sport Medicine 17, no 4 (2007): 234–41. https://doi.org/10.1097/JSM.0b013e3180f60afc.
Reason for exclusion: *No strength assessment*

PFLUEGLER, G. et al. "The Immediate Effects of Passive Hip Joint Mobilization on Hip Abductor/External Rotator Muscle Strength in Patients with Anterior Knee Pain and Impaired Hip Function. A Randomized, Placebo-Controlled Crossover Trial". Journal of Manual & Manipulative Therapy 29, no 1 (2021): 14–22. https://doi.org/10.1080/10669817.2020.1765625.
Reason for exclusion: *No clinical measures*

POPOVITZ, L. "Comparing the Outcome of Electrical Stimulation and Physical Therapy in Patellofemoral Pain Syndrome". https://clinicaltrials.gov/show/NCT03184545. John Wiley & Sons, Ltd., 2018. https://www.cochranelibrary.com/central/doi/10.1002/central/CN-01494912/full.
Reason for exclusion: *RCT Protocol*

RABIEI, P.; LETAFATKAR, A. "Effect of Education and Stabilization Training in Patients with Patellofemoral Pain". https://trialsearch.who.int/Trial2.aspx?TrialID=JPRN-UMIN000044439. John Wiley & Sons, Ltd., 2021. https://www.cochranelibrary.com/central/doi/10.1002/central/CN-02281122/full.
Reason for exclusion: *RCT Protocol*

RATHLEFF, M.S. et al. "Effect of Exercise Therapy on Neuromuscular Activity and Knee Strength in Female Adolescents with Patellofemoral Pain—An Ancillary Analysis of a Cluster Randomized Trial". Clinical Biomechanics 34 (2016): 22–29. https://doi.org/10.1016/j.clinbiomech.2016.03.002.
Reason for exclusion: *No clinical outcomes*

RAZEGHI, M. et al. Could Hip and Knee Muscle Strengthening Alter the Pain Intensity in Patellofemoral Pain Syndrome? Iranian Red Crescent Medical Journal. 12(2):104-110. 2010.
Reason for exclusion: *No continuous data*

RICE, M.A. et al. "Comparison of Two Exercises on VMO and VL EMG Activity and Force Production". Isokinetics and Exercise Science 5, no 2 (1995): 61–67. https://doi.org/10.3233/IES-1995-5201.
Reason for exclusion: *Non-inclusion of patellofemoral pain*

ROUSH, M.B. et al. "Anterior Knee Pain: A Clinical Comparison of Rehabilitation Methods": Clinical Journal of Sport Medicine 10, no 1 (2000): 22–28. https://doi.org/10.1097/00042752-200001000-00005.
Reason for exclusion: *Non-specified population*

ŞAHAN, T.Y. et al. "Investigation of New Application Technique Named Star Taping in Patellofemoral Pain: A Randomized, Single-Blind, and Placebo-Controlled Study". Somatosensory & Motor Research, 2023, 1–8. https://doi.org/10.1080/08990220.2023.2191703.
Reason for exclusion: *No strength assessment*

SELHORST, M. et al. “Evaluation of a Treatment Algorithm for Patients with Patellofemoral Pain Syndrome: A Pilot Study”. International Journal of Sports Physical Therapy. 2015, Vol. 10 Issue 2, p178-188.
Reason for exclusion: *No strength assessment*

SHADLOO, N. et al. “A Comparison between Whole-Body Vibration and Conventional Training on Pain and Performance in Athletes with Patellofemoral Pain”. Journal of Bodywork and Movement Therapies 27 (2021): 661–66. https://doi.org/10.1016/j.jbmt.2021.03.003.
Reason for exclusion: *No strength assessment*

SHARIF, F. et al. “Effects of Strengthening of Hip Abductors and Lateral Rotators for Improving Pain and Functional Limitation in Patients with Patellofemoral Dysfunction”. Rawal Medical Journal: Vol. 45. No. 1, 2020.
Reason for exclusion: *No strength assessment*

SILVA, N.C. et al. “Effects of Neuromuscular Training and Strengthening of Trunk and Lower Limbs Muscles in Women with Patellofemoral Pain: A Protocol of Randomized Controlled Clinical Trial, Blinded”. Trials, 2019. https://trialsjournal.biomedcentral.com/articles/10.1186/s13063-019-3650-7.
Reason for exclusion: *RCT Protocol*

SINGER, B. J. et al. “Treatment of Refractory Anterior Knee Pain Using Botulinum Toxin Type A (Dysport) Injection to the Distal Vastus Lateralis Muscle: A Randomised Placebo Controlled Crossover Trial”. British Journal of Sports Medicine 45, no 8 (2011): 640–45. https://doi.org/10.1136/bjsm.2009.069781.
Reason for exclusion: *Pharmacological treatment*

SOLEIMANI, F. et al. “Effectiveness of Knee Exercises versus Combined Knee and Hip Exercises in Treatment of Patellofemoral Pain: A Randomized Clinical Trial”. Majallah-i ʻIlmī-i Dānishgāh-i ʻUlūm-i Pizishkī-i Simnān 19, no 3 (2017): 554–64.
Reason for exclusion: *Non-English paper*

SOUZA, R.B.; POWERS, C.M. “Differences in Hip Kinematics, Muscle Strength, and Muscle Activation Between Subjects With and Without Patellofemoral Pain”. Journal of Orthopaedic & Sports Physical Therapy 39, no 1 (2009): 12–19. https://doi.org/10.2519/jospt.2009.2885.
Reason for exclusion: *Non-Randomized Clinical Trial*

STIENE, H.A. et al. “A Comparison of Closed Kinetic Chain and Isokinetic Joint Isolation Exercise in Patients With Patellofemoral Dysfunction”. Journal of Orthopaedic & Sports Physical Therapy 24, no 3 (1996): 136–41. https://doi.org/10.2519/jospt.1996.24.3.136.
Reason for exclusion: *Non-specified population*

STOKES, W. “Clinical Efficacy of A Home-Based Functional Exercise Program in Patients With Patellar Femoral Syndrome Over a 10-Week Period”. Clinical trial registration, 2019. https://clinicaltrials.gov/study/NCT03407365.
Reason for exclusion: *RCT Protocol*

TAZESH, B.; HALABCHI, F. “The Effect of Core Stability Training on Anterior Knee Pain”. https://trialsearch.who.int/Trial2.aspx?TrialID=IRCT201111168117N1. John Wiley & Sons, Ltd., 2019. https://www.cochranelibrary.com/central/doi/10.1002/central/CN-01868217/full.
Reason for exclusion: *RCT Protocol*

TAZESH, B. et al. “Additional Effects of Core Stability Exercises on Pain and Function of Patients with Patellofemoral Pain: A Randomized Controlled Trial”. Journal of Orthopaedics, Trauma and Rehabilitation, 2021, 221049172198907. https://doi.org/10.1177/2210491721989075.
Reason for exclusion: *No strength assessment*

TELLES, G. et al. “The Effect of Adding Myofascial Techniques to an Exercise Programme for Patients with Anterior Knee Pain – A Randomized Clinical Trial”. Manual Therapy 25 (2016): e151–52. https://doi.org/10.1016/j.math.2016.05.293.
Reason for exclusion: *No strength assessment*

VAN TIGGELEN, D. et al. “The Effects of 6-Weeks Patellofemoral Bracing on Quadriceps Muscle Function”. Isokinetics and Exercise Science 19, no 3 (2011): 169–73. https://doi.org/10.3233/IES-2011-0411.
Reason for exclusion: *Non-inclusion of patellofemoral pain*

WANG, X.; HU, H. “Effects and Mechanism of Neuromuscular Training on Patellofemoral Pain Syndrome”. https://trialsearch.who.int/Trial2.aspx?TrialID=ChiCTR1800014995. John Wiley & Sons, Ltd., 2019. https://www.cochranelibrary.com/central/doi/10.1002/central/CN-01899441/full.
Reason for exclusion: *RCT Protocol*

WITVROUW, E. et al. “Open versus Closed Kinetic Chain Exercises in Patellofemoral Pain: A 5-Year Prospective Randomized Study”. The American Journal of Sports Medicine 32, no 5 (2004): 1122–30. https://doi.org/10.1177/0363546503262187.
Reason for exclusion: *Follow-up data*

WITVROUW, E. et al. “The Effect of Exercise Regimens on Reflex Response Time of the Vasti Muscles in Patients with Anterior Knee Pain: A Prospective Randomized Intervention Study: Exercise Effect on Reflex Time Vasti Muscles”. Scandinavian Journal of Medicine & Science in Sports 13, no 4 (2003): 251–58. https://doi.org/10.1034/j.1600-0838.2003.00311.x.
Reason for exclusion: *No strength assessment*

WONG, YM. “Effect of resistance training on active and passive patellar stabilizers”. Effect of Resistance Training on Active & Passive Patellar Stabilizers. Hong Kong Polytechnic University (Hong Kong), 2008. (UMI Order AAI3341127).
Reason for exclusion: *Thesis*

WU, Z. et al. “Effects of Whole-Body Vibration plus Hip-Knee Muscle Strengthening Training on Adult Patellofemoral Pain Syndrome: A Randomized Controlled Trial”. Disability and Rehabilitation 44, no 20 (2022): 6017–25. https://doi.org/10.1080/09638288.2021.1954703.
Reason for exclusion: *No strength assessment*

XIAOHUI, H; WU, Z. “Effects and mechanism of hip-knee muscle strengthening training combined with whole-body vibration training on adult patellofemoral pain syndrome”. https://www.cochranelibrary.com/central/doi/10.1002/central/CN-02434642/full.
Reason for exclusion: *RCT Protocol*

YUE, W. et al. “A Study on the Effects of Gluteal Muscle Activation on the Electromyography of Lower Limb Muscles in Young Male Patients With Patellofemoral Pain Syndrome”. Orthop Surg. 2025 Mar;17(3):744-752. doi: 10.1111/os.14320.
Reason for exclusion: *No strength assessment*

ZAMBOTI, C.L. et al. “Analysis of Pain, Functional Capacity, Muscular Strength and Balance in Young Women with Patellofemoral Pain Syndrome”. Fisioterapia Em Movimento 30, no 3 (2017): 433–41. https://doi.org/10.1590/1980-5918.030.003.ao01.
Reason for exclusion: *Non-Randomized Clinical Trial*

ZHUANG, L. “Clinical Effects of NMES Combined with Blood Flow Restriction during Static Intervention in Patients with Patellofemoral Pain Syndrome”. https://www.chictr.org.cn/showproj.html?proj=279657
Reason for exclusion: *RCT Protocol*

ZIA, A. “Effect of Exercises in Anterior Knee Pain”. https://trialsearch.who.int/Trial2.aspx?TrialID=IRCT20181023041432N1. John Wiley & Sons, Ltd., 2021. https://www.cochranelibrary.com/central/doi/10.1002/central/CN-02281498/full.
Reason for exclusion: *RCT Protocol*

Supplementary Material E. Individual characteristics of the included interventions.

Study	Population	Interventions	n	Age (years)	Height (cm)	Weight (kg)	BMI (kg/m^2)	Symptoms duration	Outcomes: Pain and Function	Outcomes: Strength	Settings	Funding
Abdelmohsen, et al. 2025	Women with PFP	G1: Weight-bearing exercise	15	29.9 (5.3)	164.9 (7.8)	63.1 (11.8)	24.4 (3.0)	> 4 weeks	VAS, PPT	Isokinetic dynamometer *Muscle:* HABD, HAD, HE, HF, KE and KF	NI	No
		G2: Control group	15	30.1 (5.1)	164.1 (7.4)	62.7 (10.2)	23.9 (3.0)					
Abdo, et al. 2025	People with PFP (mixed sex)	G1: Exercise and manual therapy	25	21.9 (2.4)	166.2 (9.5)	68.1 (9.0)	24.8 (3.6)	> 4 weeks	VAS, AKPS	Handheld dynamometer *Muscles:* HABD, HER and KE	Clinical	Yes
		G2: Exercise only	28	22.6 (3.7)	167.2 (8.3)	64.8 (11.5)	23.2 (3.6)					
Aghakeshizadeh, et al. 2021	Recreational athletes (mixed sex)	G1: Exercise with external focus	23	29.9 (8.0)	163.7 (3.9)	63.7 (3.9)	23.9 (1.3)	74 (6-80) mo	VAS, AKPS	Handheld dynamometer *Muscles:* HABD and HER	NI	NI
		G2: Exercise with internal focus	23	28.6 (7.7)	164.6 (3.6)	64.1 (5.6)	23.7 (1.9)	65 (5-70) mo				
		G3: Control group	24	28.9 (6.5)	163.6 (3.8)	63.5 (4.3)	23.7 (1.7)	43 (4-47) mo				
Ahmadi, et al. 2020	Males with PFP	G1: Neurofeedback training	16	25.1 (2.1)	174.6 (4.1)	75.6 (5.6)	24.8 (1.5)	> 6 weeks	VAS	Handheld dynamometer *Muscles:* HABD and KE	Research laboratory	NI
		G2: Control group	16									
Almeida, et al. 2021	Women with PFP	G1: Anteromedial hip group	26	24.9 (5.0)	160.0 (70.0)	63.9 (9.6)	24.8 (3.6)	47.3 (39.6) mo	NPRS, AKPS	Handheld dynamometer *Muscles:* HABD, HAD, HER and HIR	Clinical	Yes
		G2: Posterolateral hip group	26	24.9 (4.6)	163.0 (70.0)	61.2 (9.6)	23.0 (3.5)	50.8 (38.9) mo				
Alonazi, et al. 2021	Male athletes	G1: EMG-BF-guided isometric training with patellar taping	30	25.9 (1.5)	167.6 (2.5)	66.5 (3.9)	23.8 (0.9)	NI	VAS, AKPS	Isokinetic dynamometer *Muscle:* KE	Clinical	Yes
		G2: Sham EMG-BF training with isometric exercise program	30	26.8 (2.2)	168.5 (3.2)	67.8 (4.2)	24.1 (0.8)					
Arslan, et al. 2023	Women with PFP	G1: Home exercise	20	33.4 (6.7)	162.6 (5.9)	78.9 (20.4)	NI	≥ 3 mo	VAS, AKPS	Handheld dynamometer *Muscles:* KE, KF and HPL	Home	No
		G2: Online supervised exercise	20	35.5 (5.8)	160.5 (6.2)	75.1 (12.1)						
		G3: Control group	20	30.7 (7.6)	161.7 (5.6)	69.9 (13.5)						
Asif, et al. 2022	Sprinters (mixed sex)	G1: Knee exercises and weight bearing hip strengthening	14	24.1 (4.5)	NI			> 1 mo	NPRS, AKPS	Handheld dynamometer *Muscles:* KE, KF, HABD and HE	NI	NI
		G2: Knee exercises and strengthening exercises of hip with resistance	14	22.8 (4.3)								
Aytar, et al. 2011	Women with PFP	G1: Kinesiotape	12	22.4 (1.6)	NI		20.6 (2.3)	16.2 (9.7) mo	VAS	Isokinetic dynamometer *Muscle:* KE	NI	NI
		G2: Placebo Kinesiotape	10	26.2 (3.5)			21.9 (2.2)	13.7 (8.0) mo				
Azab, et al. 2022	Teenagers with PFP (mixed sex)	G1: Physiotherapy plus Pilates	17	15.7 (1.1)	156.0 (7.0)	54.4 (5.6)	22.1 (1.2)	23.1 (5.8) mo	VAS, AKPS	Handheld dynamometer *Muscles:* KE, HABD and HER	Clinical	No
		G2: Physiotherapy program	17	15.2 (1.1)	152.0 (10.0)	50.5 (7.9)	21.8 (1.3)	26.2 (6.7) mo				

Baldon, et al. 2014	Female recreational athletes	G1: Functional stabilization	15	22.7 (3.2)	166.0 (10.0)	57.1 (8.2)	20.6 (2.0)	60 (3-156) mo	VAS, LEFS, SLTH	Isokinetic dynamometer *Muscles:* HABD, HAD, HER, HIR, KE and KF	Research laboratory	Yes
		G2: Standard training	16	21.3 (2.6)	160.0 (10.0)	58.3 (7.3)	22.3 (2.5)	27 (3-180) mo				
Basbug, et al. 2022	Sedentary female with PFP	G1: Progressive neuromuscular exercise plus taping	15	34.1 (8.9)	166.0 (7.9)	66.2 (15.8)	23.9 (5.1)	> 6 mo	VAS	Isokinetic dynamometer *Muscles:* KE and KF	University	No
		G2: Progressive neuromuscular exercise only	15	39.0 (6.4)	165.7 (5.5)	64.8 (13.3)	23.9 (5.3)					
Bily, et al. 2008	People with PFP (mixed sex)	G1: Physiotherapy plus EMS	16	27.0 (7.7)	174.5 (9.3)	68.8 (13.7)	NI	12 (6–24) mo	VAS, AKPS	Strain gauge *Muscles:* KE	NI (PT) Home (EMS)	Yes
		G2: Physiotherapy only	13	23.7 (5.5)	168.7 (4.9)	59.4 (5.7)		16 (6–24) mo				
Boitrago, et al. 2021	Women with PFP	G1: Exercise	30	27.3 (7.7)	163.0 (6.0)	62.0 (11.1)	NI	> 2 mo	NPRS, AKPS, KOS-ADLS	Handheld dynamometer *Muscles:* KE, HABD and HER	Clinical	No
		G2: Guidance	30	25.2 (5.6)	164.0 (7.0)	64.2 (9.9)						
Callaghan, et al. 2001	People with PFP (mixed sex)	G1: Experimental stimulation	7	29.6 (5.9)	NI		26.4 (4.4)	≥ 6 mo but < 3 years	VAS, AKPS	Isokinetic dynamometer *Muscle:* KE	Home	Yes
		G2: Standard stimulation	7									
Callaghan, et al. 2004	People with PFP (mixed sex)	G1: Experimental stimulation	37	36.5 (13.6)	NI		25.7 (5.7)	122 (61) mo	VAS, AKPS	Isokinetic dynamometer *Muscle:* KE	Home	Yes
		G2: Standard stimulation	37	33.2 (9.4)			25.9 (5.9)	103 (62) mo				
Celik, et al. 2019	People with PFP (mixed sex)	G1: Strengthening exercises plus NMES	14	39.1 (9.1)	166.2 (6.4)	75.1 (13.4)	NI	NI	AKPS, LKSS	Isokinetic dynamometer *Muscle:* KE and KF	NI	No
		G2: Strengthening exercises only	13	41.5 (12.7)	162.3 (8.4)	66.3 (11.0)						
Cho, et al. 2025	Female runners with PFP	G1: Hip and knee instrument-assisted soft tissue mobilization	14	23.4 (2.5)	162.0 (5.0)	55.9 (5.2)	21.4 (1.4)	> 2 mo	VAS, Step down	Handheld dynamometer *Muscles:* HABD and HER	Research laboratory	No
		G2: Knee instrument-assisted soft tissue mobilization	14	25.7 (4.2)	165.7 (4.66)	58.5 (5.7)	20.4 (1.4)					
Clark, et al. 2000	People with AKP (mixed sex)	G1: Exercise plus tape	20	26.0 (7.4)	NI		24.8 (5.7)	> 3 mo	VAS, WOMAC	Modified Tornvall chair *Muscle:* KE	NI	NI
		G2: Exercise	20	29.5 (6.2)			24.9 (4.2)					
		G3: Tape	19	29.3 (6.8)			25.0 (3.9)					
		G4: Education	22	27.1 (7.2)			25.2 (4.2)					
Constantinou, et al. 2022	People with PFP (mixed sex)	G1: Strengthening with BFR	30	25.5 (14)	170.0 (10.0)	72.5 (11.1)	24.6 (3.0)	14 (16) mo	VAS, AKPS, Step down	Handheld dynamometer *Muscles:* KE, HE and HABD	Research laboratory	NI
		G2: Standard strengthening	30	30.5 (16)	170.0 (13.0)	72.4 (16.9)	24.7 (4.3)	11 (17) mo				
Corum, et al. 2018	Women with PFP	G1: Whole body vibration training plus home exercise	18	32.7 (7.3)	161.0 (5.7)	63.1 (11)	24.2 (4.2)	> 3 mo	VAS, AKPS	Isokinetic dynamometer *Muscles:* KE and KF	Clinical (vibration training) and home	No
		G2: Home exercise only	16	33.7 (7.7)	163.0 (6.3)	63.0 (9.8)	23.5 (3.1)					

Dolak, et al. 2011	Women with PFP	G1: Hip strengthening	17	25.0 (5.0)	166.0 (8.0)	NI	24.0 (4.0)	36 (34) mo	VAS, LEFS	Handheld dynamometer *Muscles:* HABD, HER and KE	Research laboratory	Yes
		G2: Quadriceps strengthening	16	26.0 (6.0)	166.0 (8.0)		27.0 (6.0)	27 (34) mo				
Drew, et al. 2017	People with PFP (mixed sex)	G1: Matched treatment	13	29.1 (6.3)	NI		25.9 (4.8)	30 (16.5-75.3) mo	NPRS, AKPS	Isokinetic dynamometer *Muscles:* KE and HABD	Clinical	Yes
		G2: Usual care	11	29.3 (5.5)			27.7 (7.9)	33 (10.5-54) mo				
Emamvirdi, et al. 2019	Amateur female volleyball players	G1: Valgus control exercises	32	22.1 (5.9)	162.5 (7.6)	59.6 (3.7)	22.1 (1.6)	> 8 weeks	VAS, Hop tests	Isokinetic dynamometer *Muscles:* HABD, HAD, HER and HIR	Research laboratory	NI
		G2: Written instructions	32	23.1 (6.5)	164.4 (8.1)	58.6 (5.0)	21.2 (1.0)					
Esculier, et al. 2017	Runners (mixed sex)	G1: Education plus gait retraining	23	28.4 (6.8)	169.3 (7.9)	66.3 (9.9)	NI	28.0 (42.4) mo	VAS, KOS-ADLS	Handheld dynamometer *Muscles:* KE, HER, HABD and HE	Clinical	Yes
		G2: Education plus exercises	23	33.2 (6.5)	168.8 (9.6)	68.3 (16.9)		42.2 (47.4) mo				
		G3: Education only	23	30.7 (5.3)	171.4 (9.4)	68.1 (15.2)		16.4 (16.3) mo				
Ferber, et al. 2015	People with PFP (mixed sex)	G1: Knee-focused rehabilitation	88	29.0 (7.1)	170.4 (9.4)	67.6 (13.5)	NI	> 4 weeks	VAS, AKPS	Handheld dynamometer *Muscles:* HABD, HE, HIR, HER and KE	Research laboratory	Yes
		G2: Hip and core strengthening	111									
Giles, et al. 2017	People with PFP (mixed sex)	G1: Quadriceps strengthening with BFR	40	28.5 (5.2)	171.3 (9.2)	75.3 (15.7)	NI	31.6 (40.9) mo	VAS, AKPS	Isokinetic dynamometer *Muscle:* KE	NI	NI
		G2: Standard strengthening	39	26.7 (5.5)	172.7 (9.4)	77.3 (18.9)		37.8 (55.5) mo				
Girardi, et al. 2022	Women with PFP	G1: Quadriceps strengthening with Kaatsu Training	8	23.2 (2.0)	162.0 (6.0)	58.0 (6.4)	22.0 (2.1)	NI	NPRS, AKPS	Handheld dynamometer *Muscle:* KE	NI	No
		G2: Standard strengthening	7	28.5 (3.9)	159.0 (3.0)	60.6 (12.2)	23.7 (4.7)					
Glaviano, et al. 2019	People with PFP (mixed sex)	G1: Rehabilitation with PENS	11	23.8 (5.6)	169.1 (7.3)	68.2 (11.4)	NI	26.3 (26.3) mo	VAS, AKPS, ADLS, LEFS	Handheld dynamometer *Muscles:* KE, KF, HABD, HE. HIR and HER	Research laboratory	Yes
		G2: Rehabilitation with sham	10	23.0 (3.7)	166.7 (7.8)	69.8 (19.0)		23.0 (27.8) mo				
Hammami, et al. 2024	Overweight or obese women with PFP	G1: Isokinetic muscle strengthening	12	53.5 (5.1)	158.0 (6.0)	85.4 (11.0)	34.4 (4.6)	NI	KOOS-pain, SCT, 10m Walk Test, Chair Stand Test	Isokinetic dynamometer *Muscles:* KE and KF	Clinical	No
		G2: Passive compensation of movement	12	50.8 (9.5)	159.0 (8.0)	87.3 (8.5)	34.7 (4.0)					
Hansen, et al. 2023	People with PFP (mixed sex)	G1: Quadriceps-focused exercise	100	27.2 (6.3)	172.4 (8.5)	68.2 (12.4)	22.8 (3.0)	47.3 (49.4) mo	NPRS, AKPS, KOOS	Handheld dynamometer *Muscles:* KE, KF, HABD, HAD, HER, HIR, HF and HE	Home	No
		G2: Hip-focused exercise	100	27.2 (6.7)	173.2 (10.7)	67.6 (13.0)	22.4 (3.0)	52.8 (54.1) mo				
Hasan, et al. 2022	Male athletes	G1: EMG-BF strengthening plus taping	30	26.8 (1.4)	170.0 (10.0)	69.4 (2.1)	24.8 (0.7)	> 8 weeks	VAS, AKPS, SLTH	Isokinetic dynamometer *Muscle:* KE	Clinical	Yes

		G2: Control group	30	27.2 (1.4)	170.0 (20.0)	68.9 (2.0)	24.8 (0.7)					
Hasan, et al. 2025	Women with PFP	G1: Quadriceps exercise and patellar taping	30	26.0 (1.0)	157.0 (1.5)	67.7 (3.1)	27.5 (1.3)	NI	NPRS, AKPS, SLTH	Isokinetic dynamometer *Muscle:* KE	NI	Yes
		G2: Quadriceps exercise and placebo taping	30	26.2 (0.9)	157.2 (1.6)	67.4 (3.0)	27.3 (1.0)					
Herrington, et al. 2007	Men with PFP	G1: Non-weight bearing single-joint quadriceps exercise	15	26.9 (5.6)	NI				VAS, AKPS	Isokinetic dynamometer *Muscle:* KE	Hospital	NI
		G2: Weight-bearing multiple-joint quadriceps exercise	15									
		G3: Control group	15									
Hong, et al. 2023	People with PFP (mixed sex)	G1: Tailored exercise program with health education	19	31.8 (5.5)	170.0 (10.0)	67.3 (9.6)	NI	> 4 weeks	VAS, AKPS	Isokinetic dynamometer *Muscles:* KE and KF	Home	Yes
		G2: Health education	19	32.3 (7.0)	170.0 (10.0)	67.5 (10.2)						
Hott, et al. 2019	People with PFP (mixed sex)	G1: Hip-focused exercise	39	27.8 (8.6)	NI		NI	> 3 mo	VAS, AKPS, Step down	Force sensor *Muscles:* KE, HABD and HER	Clinical	NI
		G2: Knee-focused exercise	37	28.5 (6.2)								
		G3: Free physical activity	36	26.3 (7.0)								
Ismail, et al. 2013	People with PFP (mixed sex)	G1: Closed kinetic chain exercises with hip strengthening	16	20.8 (2.7)	163.6 (8.5)	64.5 (9.6)	NI	> 6 weeks	VAS, AKPS	Isokinetic dynamometer *Muscles*: HABD and HER	Clinical	NI
		G2: Closed kinetic chain exercises	16	21.2 (3.2)	165.7 (5.3)	66.6 (9.8)						
Kamel, et al. 2024	People with PFP (mixed sex)	G1: Short foot exercise plus hip and knee exercises	14	21.3 (1.3)	171.0 (12.0)	71.9 (12.4)	24.4 (2.3)	> 6 weeks	VAS, AKPS	Handheld dynamometer *Muscles:* KE and HABD	Clinical	Yes
		G2: Hip and knee exercises	14	22.5 (3.0)	170.0 (11.7)	70.7 (13.7)	24.2 (2.7)					
Keet, et al. 2007 (crossover design)	People with and without PFP (mixed sex)	G1: Experimental group	15	29.1 (5.1)	NI	65.2 (9.6)	NI	124.0 (196.0) weeks	VAS, Step-up and step-down	Isokinetic dynamometer *Muscle*: KE	NI	Yes
		G2: Healthy cohort	20	29.4 (4.6)		64.4 (11.1)		NA				
Khayambashi, et al. 2012	Women with PFP	G1: Hip abductor and external rotator strengthening	14	28.9 (5.8)	158.2 (5.8)	60.8 (10.4)	NI	> 6 mo	VAS, WOMAC	Handheld dynamometer *Muscles:* HABD and HER	NI	Yes
		G2: Control group	14	30.5 (4.8)	160.9 (4.6)	62.6 (10.6)						
Khojasteh et al. 2024	People with PFP (mixed sex)	G1: Physiotherapy + EMG-BF	15	27.4 (7.7)	155.2 (4.4)	62.9 (5.6)	25.9 (2.1)	> 2 mo	VAS	Load cell *Muscles:* HABD and HER	NI	No
		G2: Physiotherapy	15	26.2 (7.1)	155.2 (3.4)	65.8 (4.3)	26.4 (1.4)					
Kısacık, et al. 2021	People with PFP (mixed sex)	G1: Short Foot exercise	15	39.6 (8.9)	167.7 (12.2)	71.3 (16.3)	25.4 (5.2)	> 6 mo	VAS, AKPS	Handheld dynamometer *Muscles:* KE, KF, HABD, HE, AD, AP and FHL	Clinical	Yes
		G2: Control group	15	43.6 (7.8)	165.1 (7.6)	68.4 (10.7)	25.1 (3.8)					

Kong, et al. 2025	People with PFP (mixed sex)	G1: Low-load resistance training with BFR	21	20.0 (19-21)	175.0 (7.7)	67.9 (11.2)	NI	10.1 (3-28.5)	VAS, AKPS	Isokinetic dynamometer *Muscle*: KE	Clinical	Yes
		G2: High-load resistance training with sham BFR	21	20.2 (19.5 -21)	177.1 (8.2)	69.2 (9.5)		12.0 (5-24)				
Kowall, et al. 1996	People with PFP (mixed sex)	G1: Physiotherapy plus taping	12	29.0 (14-40)	NI			2.5 years (1 month – 15 years)	VAS	Isokinetic dynamometer *Muscle:* KE	Home	No
		G2: Physical therapy only	13									
Kumar, et al. 2024	People with PFP (mixed sex)	G1: Positional release	32	29.5 (5.8)	NI			1-3 mo	VAS, AKPS	Handheld dynamometer *Muscle:* HABD	Clinical	No
		G2: Myofascial release	32	29.5 (5.4)								
Kurt et al. 2016	People with PFP (mixed sex)	G1: Kinesiotape	55	31.6 (6.9)	168.3 (10.7)	69.6 (14.7)	23.7 (2.4)	19.3 (5.5) mo	VAS, AKPS	Isokinetic dynamometer *Muscle:* KE, KF	Clinical	NI
		G2: Placebo taping	51	30.9 (7.2)	167.9 (9.8)	68.7 (15.3)	23.2 (3.0)	21.1 (4.5) mo				
Lee, et al. 2020	People with PFP (mixed sex)	G1: Dynamic hamstring stretching plus rehabilitation	21	25.1 (9.2)	173.3 (2.9)	70.7 (7.0)	28.5 (3.4)	NI	VAS, AKPS	Isokinetic dynamometer *Muscles:* KE and KF	Clinical (stretch) Home (protocol)	Yes
		G2: Static hamstring stretching plus conservative rehabilitation	25	27.2 (7.0)	170.1 (5.0)	65.8 (10.4)	26.4 (3.1)					
Lee, et al. 2021	People with PFP (mixed sex)	G1: Dynamic quadriceps stretching	24	28.1 (4.2)	171.8 (4.3)	71.3 (5.4)	26.7 (2.6)	4.1 (1.6) mo	VAS, AKPS	Isokinetic dynamometer *Muscle:* KE	Clinical and home	Yes
		G2: Static quadriceps stretching	20	26.5 (6.8)	173.4 (4.0)	68.9 (8.2)	25.1 (3.2)	3.8 (2.1) mo				
Lee, et al. 2025	People with PFP (mixed sex)	G1: Digital therapeutic app	13	33.6 (11.0)	NI		21.3 (1.9)	48.4 (22.0) weeks	NPRS, AKPS	Isokinetic dynamometer *Muscle:* KE	Home (G1) Clinical (G2)	Yes
		G2: Conventional treatment	13	34.8 (10.9)			21.3 (2.0)	49.7 (26.3) weeks				
Lee, et al. 2026	People with PFP (mixed sex)	G1: Exercise with BFR	20	28.0 (2.8)	168.0 (6.2)	66.6 (12.4)	23.3 (3.4)	> 4 weeks	PPT	Traction dynamometer *Muscles:* KE and KF	NI	NI
		G2: Exercise only	19	27.6 (1.9)	168.0 (6.9)	66.7 (10.7)	23.3 (2.8)					
León-Morillas, et al. 2024	People with PFP (mixed sex)	G1: Standard physiotherapy	25	48.6 (10.3)	169.0 (9.7)	64.7 (9.1)	NI	> 3 mo	VAS, AKPS, LEFS	Handheld dynamometer *Muscle:* KE	NI	No
		G2: Flossing band plus physiotherapy	25	46.6 (11.9)	167.0 (7.2)	66.0 (8.6)						
Liu, et al. 2023	People with PFP (mixed sex)	G1: Instrument-assisted soft tissue mobilization	13	21.7 (2.0)	176.2 (3.9)	NI	71.6 (11.4)	20.8 (16.3) mo	VAS, LKSS	Muscle strength testing system *Muscles:* lower extremity extensors	Research laboratory	No
		G2: Tui-na manipulation	12	21.3 (2.5)	171.0 (9.3)		65.5 (15.6)	12.9 (8.3) mo				
Liu, et al. 2023	People with PFP (mixed sex)	G1: Instrument-assisted soft tissue mobilization	13	22.0 (2.1)	176.1 (7.2)	NI	74.5 (13.9)	12.9 (8.3) mo	VAS, LKSS	Muscle strength testing system *Muscles:* lower extremity extensors	NI	No
		G2: Instrument-assisted soft tissue mobilization with BFR	13	20.9 (2.2)	170.5 (6.6)		60.7 (10.3)	22.1 (19.2) mo				
Mahmoud, et	People with	G1: Balance group	30	25.0 (6.0)	174.2 (7.1)	72.3 (6.1)	NI	> 3 mo	VAS	Isokinetic dynamometer	NI	NI

al. 2015	PFP (mixed sex)	G2: Control group	30	24.0 (6.0)	173.1 (7.1)	73.7 (5.7)				*Muscle:* HABD		
Mason, et al. 2011	People with PFP (mixed sex)	G1: Infrapatellar taping	15	45.0 (17.0)	171.0 (NI)	80.0 (NI)	27.0 (NI)	71.0 (NI) mo	VAS	Isokinetic dynamometer *Muscle:* KE	Home	No
		G2: Quadriceps strengthening	15									
		G3: Quadriceps stretching	15									
		G4: Control group	15									
Melo, et al. 2018	Women with PFP	G1: KT with tension	18	23.7 (3.8)	NI		22.7 (2.8)	NI	NPRS, AKPS	Isokinetic dynamometer *Muscle:* KE	NI	Yes
		G2: KT without tension	18									
		G3: Control group	18									
Melo, et al. 2024	Women with PFP	G1: Exercise plus EMG	17	22.4 (3.8)	NI		22.0 (2.7)	> 6 mo	NPRS, AKPS	Isokinetic dynamometer *Muscles:* KE and HABD	NI	Yes
		G2: Execise	17	25.2 (4.0)			23.6 (3.0)					
Nakagawa, et al. 2008	People with PFP (mixed sex)	G1: Strengthening of quadriceps, hip abductor and lateral rotator muscles	7	23.6 (5.9)	NI			> 4 weeks	VAS	Isokinetic dynamometer *Muscles:* KE, HABD and HER	Home	Yes
		G2: Control group	7									
Ozlu, et al. 2024	People with PFP (mixed sex)	G1: High intensity laser	15	33.1 (6.3)	167.0 (10.0)	69.3 (14.0)	24.7 (5.1)	> 3 mo	VAS, AKPS, LEFS, TUG	Handheld dynamometer *Muscles:* KE and KF	Clinical and home	Yes
		G2: Ultrasound and TENS	15	34.4 (7.0)	165.0 (70.0)	71.9 (10.7)	26.3 (4.2)					
		G3: Ultrasound and interferential	15	33.9 (6.8)	166.0 (10.0)	73.8 (12.1)	26.7 (4.1)					
Pompeo, et al. 2022	Women with PFP	G1: Distal exercise	25	28.3 (6.2)	164.0 (7.0)	62.9 (10.8)	23.3 (3.9)	> 3 mo	VAS, AKPS	Handheld dynamometer *Muscles:* KE, KF, HABD, HE, HER, FE and FI	Research laboratory	Yes
		G2: Standard rehabilitation	25	28.7 (6.4)	164.0 (6.0)	60.7 (7.4)	22.7 (2.5)					
Qureshi, et al. 2025	Women with PFP	G1: Dry needling and conventional physiotherapy	15	34.7 (5.6)	167.9 (5.6)	64.7 (4.8)	23.1 (2.2)	9.5 (2.5) mo	NPRS, AKPS, LEFS	Manual muscle testing *Muscle:* KE	Clinical	No
		G2: Conventional physiotherapy	15	33.0 (5.4)	167.9 (6.0)	63.1 (5.4)	22.5 (1.8)	7.9 (2.7) mo				
Rabelo, et al. 2017	Women with PFP	G1: Movement control and strengthening	17	25.9 (5.5)	162.0 (10.0)	57.0 (8.9)	21.8 (2.8)	46.2 (33.0) mo	NPRS, AKPS	Handheld dynamometer *Muscles*: KE, HABD and HER	Research laboratory	Yes
		G2: Strengthening only	17	25.3 (8.1)	159.0 (10.0)	57.6 (5.7)	22.8 (1.8)	49.3 (40.5) mo				
Riel, et al. 2017	Adolescents with PFP (mixed sex)	G1: Feedback group	20	16.9 (1.5)	167.5 (5.9)	62.6 (11.9)	22.3 (3.6)	> 6 weeks	VAS, AKPS	Handheld dynamometer *Muscles:* KE, HABD and HE	Home	No
		G2: Control group	20	16.5 (1.5)	167.8 (8.1)	61.8 (7.0)	22.0 (2.3)					
Rodrigues, et al. 2021	Women with PFP	G1: Strengthening plus anodic transcutaneal stimulation	14	22.9 (2.8)	156.2 (28.2)	65.0 (12.0)	NI		VAS	10RM test *Muscle:* KE	Research laboratory	Yes
		G2: Strengthening plus sham stimulation	14									

Roh, et al. 2021	People with PFP (mixed sex)	G1: Electro-cryotherapy plus exercise	15	20.6 (1.2)	170.7 (4.6)	74.8 (11.9)	25.4 (6.3)	46.1 (19.4)	VAS, LEFS	Isokinetic dynamometer *Muscle:* KE	Clinical	Yes
		G2: Exercise	15	21.8 (1.6)	173.0 (3.0)	68.7 (4.6)	22.9 (2.4)	45.0 (16.8)				
Saad, et al. 2018	Female athletes with PFP	G1: Quadriceps exercises	10	23.2 (2.5)	161.0 (7.0)	56.3 (5.9)	21.8 (1.7)	> 3 mo	VAS, AKPS	Handheld dynamometer *Muscles:* KE, KF, HABD, HAD, HER, HIR, HE and HF	Research laboratory	Yes
		G2: Hip exercises	10	22.5 (1.1)	159.0 (3.0)	55.3 (4.0)	22.0 (2.0)					
		G3: Stretching exercises	10	21.3 (1.2)	158.0 (2.0)	54.7 (2.2)	21.9 (1.3)					
		G4: Control group exercises	10	23.2 (1.0)	161.0 (6.0)	55.4 (2.0)	21.3 (1.3)					
Şahin, et al. 2016	Women with PFP	G1: Hip plus knee exercises	25	33.3 (6.5)	NI		25.5 (4.4)	8 (4–24) mo	VAS, AKPS	Isokinetic dynamometer *Muscles:* KE, HABD, HF and HER	Clinical and home	NI
		G2: Knee exercises only	25	35.0 (5.9)			26.4 (3.5)	6 (4–24) mo				
Scafoglieri, et al. 2021	People with PFP (mixed sex)	G1: Local exercise	18	21 (17–34)	172.8 (7.7)	71.8 (13.4)	NI	27 (19–80) mo	VAS, AKPS	Isokinetic dynamometer *Muscle:* KE	Clinical	No
		G2: Spinal manual therapy	25	28 (21–31)	171.0 (7.7)	66.9 (8.6)		36 (24–90) mo				
Shahnaz, et al. 2012	People with PFP (mixed sex)	G1: Biofeedback group	15	54.4 (7.7)	155.3 (4.4)	64.9 (5.6)	26.9 (2.1)	< 6 mo	VAS	Strain gauge *Muscle:* KE	NI	NI
		G2: Control group	15	55.3 (7.1)	155.2 (3.4)	65.9 (4.3)	27.3 (1.4)					
Sheikhi, et al. 2024	People with PFP (mixed sex)	G1: Education plus trunk and hip exercises	30	30.6 (6.0)	170.2 (7.8)	73.4 (10.7)	25.3 (3.5)	60.3 (30.7) mo	VAS, KOS-ADLS	Handheld dynamometer Muscles: KE, HABD, HE and HER	University	No
		G2: Trunk and hip exercises	30	32.1 (5.5)	172.4 (9.2)	71.6 (11.0)	24.1 (3.2)	57.2 (29.1) mo				
Silva, et al. 2023	Women with PFP	G1: Neuromuscular training	34	23.6 (2.8)	163.4 (7.0)	67.3 (17.7)	NI	> 3 mo	VAS, ADLS, AKPS	Handheld dynamometer *Muscles*: KE, HABD, HE and HER	Research laboratory	Yes
		G2: Strengthening	30	22.7 (3.1)	163.0 (5.8)	61.6 (10.8)						
Steinberg, et al. 2020	Female dancers with PFP	G1: Somatosensory	28	13.4 (1.0)	155.9±6.7	45.3±6.9	18.5±2.0	NI	VAS	Handheld dynamometer *Muscles:* KE, KF, HABD and HAD	NI	NI
		G2: Isometric exercises	41		158.6±7.8	50.6±9.5	19.9±2.8					
		G3: Control group	29		156.1±7.9	47.7±10.3	19.4±2.8					
Talbot, et al. 2020	Active duty military with PFP (mixed sex)	G1: NMES with home exercise	33	26.5 (6.1)	NI				VAS, 30-SCST, SCT, 6-MWT, Forward step-down	Handheld dynamometer *Muscles:* KE and KF	Home	Yes
		G2: TENS with home exercise	33	26.9 (5.8)								
		G3: NMES+TENS with home exercise	30	26.7 (6.8)								
		G4: Home exercise only	34	26.8 (6.6)								
Talbot, et al. 2023	Active duty military with PFP (mixed sex)	G1: NMES plus high blood flow restriction	42	28.5 (6.0)	NI				VAS, 30-SCST, SCT, 6-MWT, step-down	Handheld dynamometer Muscles: KE, KF and HPL	Clinical and home	Yes
		G2: NMES plus low blood flow restriction	42	25.8 (5.5)								
Thomée	Women	G1: Isometric contractions	40	20.2 (3.2)	169.0 (6.4)	64.1 (8.8)	NI	43.0 (31.2)	VAS, Overall	Isokinetic dynamometer	NI	Yes

1997	with PFP	G2: Eccentric contractions						mo	Knee Function	*Muscle:* KE		
Vasconcelos, et al. 2024	People with PFP (mixed sex)	G1: Strength and power training	18	25.1 (6.3)	169.2 (7.1)	68.0 (11.8)	23.8 (3.9)	48.2 (42.3) mo	NPRS, AKPS	Isokinetic dynamometer Muscles: KE, HE and HABD	Research laboratory	Yes
		G2: Strength training	19	26.6 (8.8)	171.8 (11.2)	76.5 (15.2)	25.9 (4.2)	67.3 (48.1) mo				
Witvrouw, et al. 2000	People with PFP (mixed sex)	G1: Open kinetic chain	30	20.3 (14-33)	NI			15.1 (6 weeks -28 months)	VAS, AKPS	Isokinetic dynamometer *Muscles:* KE and KF	Hospital	No
		G2: Closed kinetic chain	30									
Won, et al. 2025	People with PFP (mixed sex)	G1: Lumbar spinal manipulation	15	28.9 (5.6)	171.5 (6.1)	73.1 (12.9)	24.7 (3.4)	NI	PPT	Traction dynamometer *Muscle:* KE	University	No
		G2: Placebo spinal manipulation	15	26.7 (3.5)	169.4 (9.3)	67.9 (16.3)	23.4 (4.1)					
Yalfani, et al. 2025	Women with PFP	G1: Forefoot strike pattern and exercise	20	38.6 (4.1)	168.8 (5.0)	59.6 (4.3)	20.9 (2.1)	> 6 weeks	VAS	Handheld dynamometer *Muscles:* HABD, HE and KE	University	No
		G2: Control group	20	39.7 (3.5)	170.5 (4.3)	58.7 (3.6)	20.3 (1.6)					
Yang, et al. 2026	People with PFP (mixed sex)	G1: EMS and exercise	22	21.3 (3.6)	173.8 (8.1)	73.2 (13.3)	NI	> 3 mo	VAS, AKPS	Isokinetic dynamometer *Muscles:* HABD, HE, HER, KE and KF	NI	Yes
		G2: Exercise only	20	20.5 (2.1)	174.7 (8.4)	69.5 (11.7)						
Yelvar, et al. 2015	Women with PFP	G1: Knee and postural exercises	22	45.4 (4.4)	160.3 (6.1)	68.8 (9.7)	NI	12.5 (7.8) mo	VAS, AKPS	Isokinetic dynamometer *Muscles:* KE and KF	Clinical (postural) Home (knee program)	NI
		G2: Knee exercises only	20	45.5 (5.5)	158.7 (4.8)	73.1 (12.9)		15.3 (9.3) mo				
Yip, et al. 2006	People with PFP (mixed sex)	G1: EMG-BF plus exercise	13	32.5 (8.8)	NI			> 6 mo	PSS	Isokinetic dynamometer *Muscle:* KE	Home	Yes
		G2: Exercise only	13									

Abbreviations: 6-MWT: Six-Minute Walk Test; 30-SCST: 30-Second Chair Stand Test; 30-second FSDT: 30-Second Forward Step-Down Test; AD: Ankle Dorsiflexors; ADLS: Activities of Daily Living; AKPS: Anterior Knee Pain Scale; AP: Ankle Plantarflexors; BFR: Blood Flow Restriction; EMG-BF: Electromyographic Biofeedback; EMS: Electric Muscle Stimulation; FE: Foot Eversors; FHL: Flexor Hallucis Longus; FI: Foot Inversors; G: group; HABD: Hip Abductors; HAD: Hip Adductors; HE: Hip Extensors; HER: Hip External Rotators; HF: Hip Flexors; HIR: Hip Internal Rotators; HPL: Hip Posterolateral muscles; KE: Knee Extensors; KF: Knee Flexors; KOS-ADL: Knee Outcome Survey – Activities of Daily Living Scale; KT: Kinesiotape; LEFS: Lower Extremity Functional Scale; LKSS: Lysholm Knee Scoring Scale; MO: months; NA: Not Applicable; NI: Not Informed; NMES: Neuromuscular Electrical Stimulation; NPRS/NPS: Numeric Pain Rating Scale; PENS: Percutaneous Electrical Nerve Stimulation; PPT: Pressure Pain Thresold; PSS: Patellofemoral Pain Severity Scale; SCT: Stair Climb Test; SLTH: Single-Leg Triple Hop; TENS: Transcutaneous Electrical Nerve Stimulation; VAS: Visual Analog Scale; WOMAC: Western Ontario and McMaster Universities Osteoarthritis Index.

Supplementary Material F. Characterization and description of the included interventions.

Study	Intervention 1	Intervention 2	Intervention 3
Abdelmohsen, et al. 2025	***“Weight-bearing Exercise for Better Balance”***<br><br>**Intervention:** Warm-up, static balance tasks with incremental restriction, graded reaching exercise, and walking practice to enhance dynamic balance and lower limb coordination.<br><br>**Duration:** 8 weeks, 3 times per week, 45-60 min per session.<br><br>**Intensity:** Four progressive stances for balance tasks. At each level of progression, participants were required to maintain static postural control for 1 minute. The number of repetitions was gradually increased from 12 to 30 over the 8 weeks.	***“Control Group”***<br><br>The control group did not receive any intervention.	X
Abdo, et al. 2025	***“Hip and Knee Exercises plus manual therapy”***<br><br>**Intervention:** Strecthing and strengthening of the hip and knee muscles. Manual therapy consisted of patellar mobilization, iliotibial band release, and deep friction massage of the lateral retinaculum.<br><br>**Duration:** 4 weeks, 12 sessions, 3 times per week, 45-60 min per session.<br><br>**Intensity:** Not informed.	***“Hip and Knee Exercises”***<br><br>**Intervention:** Strecthing and strengthening of the hip and knee muscles. Open and closed kinetic chain exercises were performed.<br><br>**Duration:** 4 weeks, 12 sessions, 3 times per week, 45-60 min per session.<br><br>**Intensity:** Not informed.	X
Aghakeshizadeh, et al. 2021	***“Hip and Knee Strengthening with Internal Feedback”***<br><br>**Intervention:** Strengthening of the hip and knee muscles. Subjects were taught to "prevent the hip to adduction", "to avoid the hip to inwardly or internally rotate" or "to keep the trunk in the straight direction".<br><br>**Duration:** 6 weeks, 18 sessions, 3 times per week**,** 40 minutes per session.<br><br>**Intensity:** Increased using ankle weights and theraband once a week. Number of sets and reps changed every two weeks.	***“Hip and Knee Strengthening with External Feedback”***<br><br>**Intervention:** Strengthening of the hip and knee muscles. The researcher provided the patients with feedback, during the exercise, using his hand and theraband to correct the wrong movement pattern.<br><br>**Duration:** 6 weeks, 18 sessions, 3 times per week**,** 40 minutes per session.<br><br>**Intensity:** Increased using ankle weights and theraband once a week. Number of sets and reps changed every two weeks.	***“Hip and Knee Strengthening”***<br><br>**Intervention:** Exercises to increase the strength of the hip and knee muscles and to improve the motor control of the lower limbs. This group did not receive any kind of feedback.<br><br>**Duration:** 6 weeks, 18 sessions, 3 times per week**,** 40 minutes per session.<br><br>**Intensity:** Increased using ankle weights and theraband once a week. Number of sets and reps changed every two weeks.

Ahmadi, et al. 2020	***"Neurofeedback training*"**<br><br>**Intervention:** The protocol included an increase in sesnosry-motor waves and descrease in beta and theta waves. Patients received the necessary audio and visual feedback vision to regulate their brain waves with animation movement; a pleasant sound was played for positive scores and the animation halt meant a negative score which was followed by an unpleasant sound .<br><br>**Duration:** 12 weeks, 3 sessions per week, 30 minutes per session.<br><br>**Intensity:** Not informed.	***"Control Group"***<br><br>The control group did not receive any intervention.	X
Almeida, et al. 2021	***"Anteromedial Hip Strengthening*"**<br><br>**Intervention:** Strengthening exercises: hip adduction in a side-lying position, flex ring squeeze in a side-lying position, and hip internal rotation with elastic resistance.<br><br>**Duration:** 6 weeks, 12 sessions, 2 times per week, 60 minutes per session.<br><br>**Intensity:** Participants ability to complete three sets of 8-12 reps and a Borg Rating of Perceived Exertion of 60-80%.	***"Posterolateral Hip Strengthening*"**<br><br>**Intervention:** Strengthening exercises: hip abduction in a side-lying position, clam with elastic resistance, and hip external rotation with elastic resistance.<br><br>**Duration:** 6 weeks, 12 sessions, 2 times per week, 60 minutes per session.<br><br>**Intensity:** Participants ability to complete three sets of 8-12 reps and a Borg Rating of Perceived Exertion of 60-80%.	X
Alonazi, et al. 2021	***"EMG-BF-guided Isometric Exercise Training with Patellar Taping"***<br><br>**Intervention:** McConnell taping and three quadriceps-strengthening exercises (3x10 rep): isometric quadriceps, terminal knee extension, and isometric hip adduction. Before each session, the threshold level was identified by the participant maximally contracting the quadriceps three times while an EMG-BF device monitored activity.<br><br>**Duration:** 4 weeks, 5 times per week.<br><br>**Intensity:** Maximally activate their muscle during the exercise, holding the contraction for 5s.	***"Sham EMG-BF Training with an Isometric Exercise Program"***<br><br>**Intervention:** Same set of exercises (3x10 rep), but with a sham EMG-BF. Placebo tape was applied in a vertical direction from the center of the patella. The electrodes were positioned away from the target muscles.<br><br>**Duration:** 4 weeks, 5 times per week.<br><br>**Intensity:** Maximally activate their muscle during the exercise, holding the contraction for 5s.	X

Arslan, et al. 2023	***“Home Exercise”***<br><br>**Intervention:** Strengthening exercises for core, knee and hips muscles, and stretching exercises directed at the lower extremity muscles. The exercises for this group were explained verbally and demonstrated, then the subjects were instructed to perform the exercises at home regularly.<br><br>**Duration:** 6 weeks, 3 times per week.<br><br>**Intensity:** Week 1-2 week, without weight; week 3-4, 3% body weight; week 5-6, 5% body weight.	***“Online Supervised Exercise”***<br><br>**Intervention:** The participants received the same protocol from the home exercise group, but were given as online supervised exercises. Online meetings were planned on the Zoom platform, and each exercise was explained verbally then performed together with the group participants.<br><br>**Duration:** 6 weeks, 3 times per week.<br><br>**Intensity:** Week 1-2 week, without weight; week 3-4, 3% body weight; week 5-6, 5% body weight.	***“Control Group”***<br><br>The control group did not receive any intervention.
Asif, et al. 2022	***“Conventional knee exercises and weight bearing hip strengthening exercises”***<br><br>**Intervention:** Conventional knee exercises were half squats, seated knee extension on each side, knee flexion on each side in prone lying, leg calf raises. Stretching for quadriceps, hamstring and ilio-tibial band. Weight bearing hip strengthening exercises were single leg glute bridge, hip extension with knee flexion in quadruped position on shoulders, hip extension with knee extension in quadruped position on shoulder, side lying hip abduction.<br><br>**Duration:** 6 weeks, 3 times per week.<br><br>**Intensity:** Not informed.	“***Conventional knee exercises and strengthening exercises of hip with resistance”***<br><br>**Intervention:** Conventional knee exercises were same as the other group. Hip strengthening exercises with resistance included all the weight bearing exercises as the other group but with the addition of resistance through Thera band/ loop band.<br><br>**Duration:** 6 weeks, 3 times per week.<br><br>**Intensity:** Not informed.	X
Aytar, et al. 2011	***“Kinesiotape”***<br><br>**Intervention:** Subjects were taped by a physical therapist certified to apply kinesiotape with a Y-Shaped kinesio tape over the quadriceps territory. Two Y strips were applied to the quadriceps.<br><br>**Duration:** 45 minutes<br><br>**Intensity***:* Not applicable.	***“Sham Kinesiotape”***<br><br>**Intervention:** Subjects were taped with a sticking plaster without stretch. The sticking plaster was similar to the kinesiotape, was easy to access and has no strech property. Application were similar to the other group.<br><br>**Duration:** 45 minutes<br><br>**Intensity:** Not applicable.	X
Azab, et al. 2022	***“Standard Physiotherapy Program”***<br><br>**Intervention:** Physiotherapy program that aimed to relieve pain, improve muscle strength, increase flexibility, and	***“Standard Physiotherapy Program plus Pilates”***<br><br>**Intervention:** Same program described on the other group plus Pilates exercises. They used Elastic bands and Pilates	X

	promote functional status. Electrotherapy (ultrasound and infrared), stretching of the muscles surrounding the hip and knee, and strengthening of the muscles around the hip and knee are all part of the program. Number of sets and reps changed every four weeks<br><br>**Duration:** 3 months, 3 sessions per week, 60 minutes per session.<br><br>**Intensity:** Not informed.	bands/ball. Number of sets and reps changed every four weeks<br><br>**Duration:** 3 months, 3 sessions per week, 85 minutes per session.<br><br>**Intensity:** Not informed.	
Baldon, et al. 2014	***"Functional Stabilization Training"***<br><br>**Intervention:** Neuromuscular, stretching, and strength exercises for trunk, hip and knee muscles, using weight-bearing activities. The patients were educated to perform the functional exercises with the lower extremities in neutral frontal plane alignment and to avoid quadriceps dominance.<br><br>**Duration:** 8 weeks, 24 sessions, 3 times per week, 90 to 120 minutes per session.<br><br>**Intensity:** The load and progression was different according to exercise type. All the description of the exercise and their progression is available in appendix.	***"Standard Training"***<br><br>**Intervention:** Strengthening exercises for knee extensor muscles. The standard group consisted of stretching, as well as traditional weight-bearing and non–weight-bearing exercises emphasizing quadriceps strengthening<br><br>**Duration:** 8 weeks, 24 sessions, 3 times per week, 90 to 120 minutes per session.<br><br>**Intensity:** The load and progression was different according to exercise type. All the description of the exercise and their progression is available in appendix.	X
Basbug, et al. 2022	***"Progressive neuromuscular exercise plus taping"***<br><br>**Intervention:** Same exercise program plus mechanical correction tape. 'I' taping was applied to the knee and foot with a moderate-to-extreme stretch.<br><br>**Duration:** 12 weeks.<br><br>**Intensity:** Subjects started with an easy length of the theraband and increased according to Omni Scale.	***"Progressive neuromuscular exercise"***<br><br>**Intervention:** Progressive neuromuscular exercise program that included lower limb strenghtening and stabilisation of the knee and hip joint.<br><br>**Duration:** 12 weeks.<br><br>**Intensity:** Subjects started with an easy length of the theraband and increased according to Omni Scale.	X

Bily, et al. 2008	***"Physiotherapy plus EMS"***<br><br>**Intervention:** The patients were instructed in a home-based self-stimulation program. Four self-adhesive electrodes were placed on both ends of the quadriceps muscles.<br><br>**Duration:** Stimulation for 12 weeks, 5 times a week, 2 times daily during 20 minutes. Exercise duration is the same as the other group.<br><br>**Intensity:** The intensity of the stimulation was kept as high as possible; however, pain tolerance and patient discomfort were modifying factors.	***"Physiotherapy only"***<br><br>**Intervention:** The training program consisted of isometric, concentric, and excentric leg raises and pulls, stepping and squatting, balance exercises and streching.<br><br>**Duration:** Daily training during the first 2 weeks. From the third week on, training with higher loads 3 times a week.<br><br>**Intensity:** A pain level of up to 2 on the visual analogue scale during the exercises was accepted for further increasing the loads. If levels were 3 or 4, the loads were kept constant; pain level above 5, the loads were reduced.	X
Boitrago, et al. 2021	***"Exercise"***<br><br>**Intervention:** The participants performed resisted exercises for quadriceps, gluteus and trunk muscles, in addition to proprioceptive exercises.<br><br>**Duration:** 6 weeks, 12 sessions, 3 times per week, 60 minutes per session.<br><br>**Intensity:** Intensity of training was increased every two weeks by expanding the training time and converting it into a more difficult exercise such as deep squats with different angles and using different degrees of resistance for elastic band exercises.	***"Guidance"***<br><br>**Intervention:** The patients in the guidance group were evaluated and received guidance regarding the pathology. After 6 weeks they were reassessed.<br><br>**Duration:** Not applicable.<br><br>**Intensity:** Not applicable.	X
Callaghan, et al. 2001	***"Experimental stimulation"***<br><br>**Intervention:** Consisted of three simultaneously delivered components: a background low-frequency, a high-frequency and a 'doublet' of pulses delivered within the higher frequency burst.<br><br>**Duration:** 6 weeks, 42 sessions, 7 days per week, 1 hour per day.<br><br>**Intensity:** Maximum of 90 mA with a duty cycle of 10:50, and the pulse duration was set at 200 µs as frequently adopted in human studies in muscle.	***"Standard stimulation"***<br><br>**Intervention:** This stimulator was a three-channel model, which generated bipolar, biphasic, asymmetrical rectangular pulses.<br><br>**Duration/Intensity:** Once a day five days a week for the first two weeks (2 minutes at 8 Hz pulse width 250 µs; 20 minutes at 35 Hz pulse width 350 µs; 3 minutes at 3 Hz pulse width 250 µs). Then three times a week for weeks 3 and 4 and twice a week for the last two weeks (2 minutes at 8 Hz pulse width 250 µs; 20 minutes at 45 Hz pulse width 350 µs; 3 minutes at 3 Hz pulse width 250 µs.).	X

Callaghan, et al. 2004	***"Experimental stimulation"***<br><br>**Intervention:** This was a two-channel portable stimulator which produced a balanced, asymmetrical biphasic pulse. Two self-adhesive electrodes were used over the quadriceps muscle group. Patients were instructed to maintain knee extension and remain in rest during stimulation.<br><br>**Duration:** 6 weeks, 42 sessions, 7 days per week, 1 hour per day.<br><br>**Intensity:** Contained 5 simultaneously delivered frequency components of 125, 83, 50, 2.5 and 2Hz.	***"Standard stimulation"***<br><br>**Intervention:** It used an asymmetric biphasic rectangular waveform. Four self-adhesive electrodes were placed over the quadriceps muscle group. Patients were instructed to maintain knee extension and not to walk or do other activities during stimulation.<br><br>**Duration:** 6 weeks, 42 sessions, 7 days per week, 1 hour per day.<br><br>**Intensity:** Received 1 uniform constant frequency component of 35Hz.	X
Celik, et al. 2019	***"Strengthening exercises plus NMES"***<br><br>**Intervention:** The same exercise program from the other group plus NMES protocol. Two self-adhesive surface electrodes were placed on the quadriceps on the motor points. NMES superimposed on voluntary contraction was applied during two different exercises. 1) Isometric quadriceps strengthening (30 isometric contractions); 2) 0–30◦ terminal knee extension (30 isotonic contractions).<br><br>**Duration:** Received 20-minute superimposed NMES after each standardized session for 18 sessions in 6 weeks.<br><br>**Intensity:** Stimulation frequency of 50Hz.	***"Strengthening exercises only"***<br><br>**Intervention:** Participants performed exercises for knee, hip and calf, plus patellar mobilization. Cold pack was given for 15 minutes after the sessions. They had an additional home exercise program including knee and hip strengthening exercises.<br><br>**Duration:** 6 weeks, 12 sessions, 3 times per week, 45 minutes per session.<br><br>**Intensity:** They performed 3 sets of 10 repetitions. Resistance NI.	X
Cho, et al. 2025	***"Hip and knee treatment"***<br><br>**Intervention:** Instrument-assisted soft tissue mobilization targeting the quadriceps, vastus medialis, and vastus lateralis trigger points, patellar borders, iliotibial band, iliotibial band-tensor fascia late interface, and iliotibial band-gluteus medius interface.<br><br>**Duration:** Single session of 7 minutes.<br><br>**Intensity:** Not applicable.	***"Knee treatment"***<br><br>**Intervention:** Instrument-assisted soft tissue mobilization session targeting the quadriceps, vastus medialis, and vastus lateralis trigger points and patellar borders.<br><br>**Duration:** Single session of 3 minutes.<br><br>**Intensity:** Not informed.	X
Clark, et al. 2000	***"Exercise, taping, and education"***<br><br>**Intervention:** This group receveid the same exercises of	***"Exercise and education"***<br><br>**Intervention:** Patients were taught hamstring, iliotibial	***"Taping and education"***<br><br>**Intervention:** Tape was applied from the lateral border of

	the "Execise and Education" group, focusing on strenghthening and stretching lower limb muscles. They also received the taping and the orientation.<br><br>**Duration:** 3 months, 1 time per day of exercise and 6 sessions of taping.<br><br>**Intensity:** Not informed.	band, quadriceps, and gastrocnemius stretches. Other functional isotonic exercises included sit to stand, proprioceptive balance work using a trampet, and specific exercises for gluteus medius and maximus. Progressive step down exercises were performed.<br><br>**Duration**: 3 months, daily.<br><br>**Intensity**: Not informed.	the patella pulling medially and upwards over the medial femoral condyle. If this did not eliminate the pain then the taping was repeated in knee flexion.<br><br>**Duration:** 3 months, 6 sessions.<br><br>**Intensity:** Not applicable.
	**Intervention 4**<br>***"Education alone"***<br><br>**Intervention:** All four groups received the same advice. This involved receiving the Arthritis Research Campaign leaflet "Knee pain in young adults" and sessions on (a) an explanation of the nature of anterior knee pain, the anatomy of the patellofemoral joint, and possible causes of anterior knee pain; (b) footwear and appropriate sporting activities; (c) pain controlling drugs; (d) stress relaxation techniques, ice and massage; (e) diet and weight advice; and (f) prognosis and self help.<br><br>**Duration:** Not informed.<br><br>**Intensity:** Not applicable.		
Constantinou, et al. 2022	***"Strengthening with blood flow restriction"***<br><br>**Intervention:** Hip and knee strengthening exercise program using blood flow restriction plus stretching. Sports Rehab Tourniquet cuffs were used. Exercises focused on strengthening the hip extensors and abductors, as well as the quadriceps both in open and closed kinetic chain. Both groups received passive stretching of the hamstrings, plantar flexors, quadriceps and iliotibial band.<br><br>**Duration:** 4 weeks, 12 sessions, 3 times per week, 1 hour per session.<br><br>**Intensity:** Mechanical resistance was used at a load of 30% of 1-RM with a limb occlusion pressure of 70%. Exercise progression was individualized for both groups with weekly repetition maximum testing. Assessments were performed at the beginning of each week.	***"Standard strengthening"***<br><br>**Intervention:** Hip and knee strengthening exercises with a combination of stretching. Exercises focused on strengthening the hip extensors, abductors and external rotators, as well as, the quadriceps and hamstrings in both open and closed kinetic chain. Both groups received passive stretching of the hamstrings, plantar flexors, quadriceps and iliotibial band.<br><br>**Duration:** 4 weeks, 12 sessions, 3 times per week, 45 minutes per session.<br><br>**Intensity:** Exercises with ankle weights or mechanical resistance were set at a load of 70% of 1-RM. Elastic resistance exercises were based on participants 10 repetitions maximum for 3 sets.	X
Corum, et al. 2018	***"Whole body vibration training plus home exercise"***<br><br>**Intervention:** Performed on oscillating vibration platform plus home exercise protocol. Lower extremity stretching	***"Home exercise only"***<br><br>**Intervention:** The patients in the control group were instructed on how to perform the exercises at home and	X

	consisted of quadriceps, hamstring, gastrocnemius and iliotibial band stretching. Four exercises of whole body vibration were applied: lunge-step, semi-squat, ball squeeze squat and dynamic squat position.<br><br>**Duration:** 8 weeks, 24 sessions, 3 times per week, 40 minutes per session.<br><br>**Intensity:** Whole body vibration used only body weight resistance with no added external weight. Home exercise NI.	supervised individually once a week throughout the program. Lower extremity stretching exercises consisted of quadriceps, hamstring, gastrocnemius and iliotibial band stretching with 3 repetitions for 30 s each muscle.<br><br>**Duration:** 8 weeks, 24 sessions, 3 times per week, 40 minutes per session.<br><br>**Intensity:** Not informed.	
Dolak, et al. 2011	***"Hip strengthening"***<br><br>**Intervention:** Both groups received the same flexibility exercises: hamstring, quadriceps triceps surae stretch. Therapeutic exercises for the first 4 weeks were specifically targeting the hip musculature. After completing the fourth week, women from both groups were instructed on the second phase of rehabilitative exercises, focused on functional weight-bearing resistance exercises an balance.<br><br>**Duration:** 8 weeks, 3 sessions per week.<br><br>**Intensity:** Variable with the exercise, participants had the goal of performing exercises against a resistance equal to 7% of their bodyweight.	***"Quadriceps strengthening"***<br><br>**Intervention:** Both groups received the same flexibility exercises: hamstring, quadriceps triceps surae stretch. Therapeutic exercises for the first 4 weeks were specifically targeting the quadriceps musculature. After completing the fourth week, women from both groups were instructed on the second phase of rehabilitative exercises, focused on functional weight-bearing resistance exercises an balance.<br><br>**Duration:** 8 weeks, 3 sessions per week.<br><br>**Intensity:** Variable with the exercise, participants had the goal of performing exercises against a resistance equal to 7% of their bodyweight.	X
Drew, et al. 2017	***"Matched treatment"***<br><br>**Intervention:** Participants were given education and justification of the treatment followed by three exercises aimed at targeting coronal, sagittal and transverse strength of the hip using resistance bands.<br><br>**Duration:** 6 weeks, 1 session per week, 30 minutes per session.<br><br>**Intensity:** To progress the load and resistance, a Borg Rate of Perceived Exertion scale was used. A scale of >6 was considered desirable and participants were monitored after a few repetitions to ensure that was being achieved.	***"Usual care"***<br><br>**Intervention:** Participants continued with the same management of their condition as they were planning to receive prior to the commencement of the study.<br><br>**Duration:** Not applicable.<br><br>**Intensity:** Not applicable.	X
Emamvirdi, et al. 2019	***"Knee Valgus Control Training"***	***"Written Instructions"***	X

	**Intervention:** Neuromuscular and strength exercises for knee and hip muscles with instruction to control knee valgus. Verbal and visual feedback were used to control movement of the pelvis and the knee in frontal plane.<br><br>**Duration:** 6 weeks, 18 sessions, 3 times per week, 60 minutes per session.<br><br>**Intensity:** The intensity of exercise was >75% 1RM and was increased every 2 weeks.	**Intervention:** The control group received written instructions that included postural corrections and tips for improving general health. They were asked to come to the clinic once or twice a week and received heat or ice treatment according to their needs.<br><br>**Duration:** 6 weeks, 1 or 2 times per week.<br><br>**Intensity:** Not applicable.	
Esculier, et al. 2017	***"Education plus gait retraining"***<br><br>**Intervention:** Together with education, runners received personalised advice on running gait modifications. Participants had a 10-minute treadmill session with physiotherapist feedback at every visit to the clinic.<br><br>**Duration:** Not informed.<br><br>**Intensity:** Progress based on the evolution of symptoms, avalaible on supplementaty material of the article.	***"Education plus exercises"***<br><br>**Intervention:** In addition to the education component, runners were asked to perform a standardised HEP.<br><br>**Duration:** 8 weeks, 7 days per week.<br><br>**Intensity:** The personalised programme included four phases of 2 weeks and gradually progressed through higher difficulty under physiotherapist guidance.	***"Education only"***<br><br>**Intervention:** Runners were asked to increase the frequency of their weekly trainings, to decrease each session's duration and speed and to avoid downhill and stairs running. Gradually, running distance was increased according to symptoms. The education group received no other instructions.<br><br>**Duration:** Not informed.<br><br>**Intensity:** Not applicable.
Ferber, et al. 2015	***"Knee-focused rehabilitation"***<br><br>**Intervention:** Patients initially performed non–weight-bearing quadriceps strengthening and then progressed to weight-bearing quadriceps-strengthening exercises. No emphasis was placed on stabilizing the core musculature before initiating any of the movements.<br><br>**Duration:** 6 weeks, 3 times per week<br><br>**Intensity:** The TheraBand resistance allowed the patient to complete 10 repetitions, with the final 3 repetitions being challenging in intensity but with the patient still able to maintain good form.	***"Hip and core strengthening"***<br><br>**Intervention:** Patients initially performed non–weight-bearing, muscle-strengthening exercises that focused on activating the hip musculature. Those exercises progressed to weight-bearing exercises, including core-strengthening and balance exercises that were designed to target the core musculature, with specific emphasis placed on stabilizing the core musculature.<br><br>**Duration:** 6 weeks, 3 times per week<br><br>**Intensity:** The TheraBand resistance was chosen based on the patient's ability to complete 10 repetitions of the exercise, with the final 3 repetitions being challenging but with the patient still able to maintain good form.	X
Giles, et al. 2017	***"Quadriceps strengthening with blood flow restriction"***<br><br>**Intervention:** Each group performed leg press between 0°	***"Standard strengthening"***<br><br>**Intervention:** Each group performed leg press between 0°	X

	and 60°, knee flexion and leg extension from 90° to 45° knee flexion. The cuff was placed on the proximal thigh and inflated to the prescribed pressure.<br><br>**Duration:** 8 weeks, 3 times per week.<br><br>**Intensity:** The exercises were performed at approximately 30% of 1RM with the blood flow restriction cuff inflated. If pain was reported to be greater than 2/10 on the visual scale the load was reduced by 20%.	and 60°, knee flexion and leg extension from 90° to 45° knee flexion. The placebo was a 5cm elastic cuff placed firmly around the proximal thigh.<br><br>**Duration:** 8 weeks, 3 times per week.<br><br>**Intensity:** Strengthening performed at 70% of 1RM, with placebo blood flow restriction. If pain was reported to be greater than 2/10 on the visual scale the load was reduced by 20%.	
Girardi, et al. 2022	***"Quadriceps strengthening with Kaatsu Training"***<br><br>**Intervention:** The treatment was conducted in a leg extension machine. Partial vascular occlusion (sphygmomanometer) was attached to the proximal region of the unilateral thigh, with insufflation pressure of 20mmHg + systolic blood pressure at rest.<br><br>**Duration:** 6 weeks, 18 sessions, 3 times per week.<br><br>**Intensity:** Not informed.	***"Standard strengthening"***<br><br>**Intervention:** The control group underwent the same protocol with the same load of the experimental group, without partial vascular occlusion and performing only a conservative exercise.<br><br>**Duration:** 6 weeks, 18 sessions, 3 times per week.<br><br>**Intensity:** Not informed.	X
Glaviano, et al. 2019	***"Patterned Electrical Neuromuscular Stimulation"***<br><br>**Intervention:** Neuromuscular, stretching, and strength exercises for trunk, hip and knee muscles, plus patterned electrical neuromuscular stimulation in gluteal and thigh muscles.<br><br>**Duration:** 4 weeks, 12 sessions, 3 times per week.<br><br>**Intensity:** The stimulus amplitude was increased until a strong motor contraction was seen by the administering clinician. Intensity and progression is described on article appendix.	***"Sham"***<br><br>**Intervention:** The participants in the sham group had an identical setup. They were told that they were receiving a "subsensory" treatment and that they should notify the researcher if anything was felt during the treatment.<br><br>**Duration:** 4 weeks, 12 sessions, 3 times per week.<br><br>**Intensity:** Intensity and progression is described on article appendix.	X
Hammami, et al. 2024	***"Rehabilitation program in Passive Compensation of Movement mode"***<br><br>**Intervention:** Patients performed a traditional program on the isokinetic dynamometer (quadriceps, hamstring, gastrosoleus muscle group, hip adductors and iliotibial band) while working in passive compensation of	***"Rehabilitation program plus an isokinetic muscle strengthening"***<br><br>**Intervention:** Standard rehabilitation program plus an isokinetic muscle strengthening.<br><br>**Duration:** 6 weeks, 12 sessions, 2 times per week.	X

	movement.<br><br>**Duration:** 6 weeks, 12 sessions, 2 times per week.<br><br>**Intensity:** Not informed.	**Intensity:** Not informed.	
Hansen, et al. 2023	***"Quadriceps-focused exercise"***<br><br>**Intervention:** Warm-up with 20 repetitions of sitting leg extension without external load. Next, sitting leg extension with load, squat and lunge.<br><br>**Duration:** 12 weeks, 36 sessions, 3 times per week.<br><br>**Intensity:** The participants were instructed to increase resistance whenever they were able to perform two repetitions more than the desired number (14 or more). The means of progression were adding elastic bands, weight in a backpack or holding dumbbells in the hands.	***"Hip-focused exercise"***<br><br>**Intervention:** Warm-up with 20 repetitions of clam-shell without external load. Next, clam-shell with load, side-lying/standing hip abduction and standing hip extension.<br><br>**Duration:** 12 weeks, 36 sessions, 3 times per week<br><br>**Intensity:** The participants were instructed to increase resistance whenever they were able to perform two repetitions more than the desired number (14 or more). The means of progression were adding elastic bands, weight in a backpack or holding dumbbells in the hands.	X
Hasan, et al. 2022	***"EMG-BF strengthening plus taping"***<br><br>**Intervention:** EMG-Biofeedback was used to help participants strengthen their quadriceps muscles. After the electrode was properly prepared and positioned, each participant was given three different exercise regimens. Each participant was guided to perform three types of quadriceps exercises: isometric quadriceps, MVIC at 30°, 60°, and 90° angles, and isometric hip adduction exercises.<br><br>**Duration:** 6 weeks, 5 days a week.<br><br>**Intensity:** Not informed.	***"Control group"***<br><br>**Intervention:** Participants in the control group were given the same series of exercises along with sham patellar taping and EMG-BF. When the patient's knee was flexed, a placebo tape was applied in a vertical manner from the center of the patella. Electrodes were placed in a location that was remote from the muscles that were being stimulated.<br><br>**Duration:** 6 weeks, 5 days a week.<br><br>**Intensity:** Not informed.	X
Hasan, et al. 2025	***"Strengthening exercise plus taping"***<br><br>**Intervention:** Patellar taping included lateral and anteroposterior tilt and medial patellar glide. Strenghtening exercises included isometric quadriceps contration at 60º knee flexion, isometric hip adduction and abduction.<br><br>**Duration:** 5 days a week, 6 weeks.<br><br>**Intensity:** Not informed.	***"Strengthening exercise plus placebo taping"***<br><br>**Intervention:** For the placebo taping, nonrigid tape as appplied vertically from the center of the patella with the knee in a flexed position. Strenghtening exercises were the same as the other group.<br><br>**Duration:** 5 days a week, 6 weeks.<br><br>**Intensity:** Not informed.	X

Herrington, et al. 2007	***"Non-weight bearing single-joint quadriceps exercise"***<br><br>**Intervention:** Patients in this group performed the knee extension exercises in a seated position from 90° of knee flexion to full extension.<br><br>**Duration:** 6 weeks, 3 times per week.<br><br>**Intensity:** The intervention program was based on 6-RM. First set was performed at 50% of 6-RM, followed by a set of 6 repetitions performed at 75% of the 6-RM. For the third set, the patient performed as many repetitions as he was able to do using 100% of the 6-RM as resistance. The number of repetitions used for the third set was used to determine the resistance for the fourth set. For the fourth set, the patient performed as many repetitions as possible using the new adjusted weight.	***"Weight-bearing multiple-joint quadriceps exercise"***<br><br>**Intervention:** Patients in this group performed the leg press exercise in a seated position from 90° of knee flexion to full extension.<br><br>**Duration:** 6 weeks, 3 times per week.<br><br>**Intensity:** The intervention program was based on 6-RM. First set was performed at 50% of 6-RM, followed by a set of 6 repetitions performed at 75% of the 6-RM. For the third set, the patient performed as many repetitions as he was able to do using 100% of the 6-RM as resistance. The number of repetitions used for the third set was used to determine the resistance for the fourth set. For the fourth set, the patient performed as many repetitions as possible using the new adjusted weight.	***"Control group"***<br><br>**Intervention:** The patients in the control group participated only in the testing procedures, and all participants were asked to avoid sports or other pain-provocative activities during the exercise intervention period but to participate in their standard army (clerical and administration) duties.<br><br>**Duration:** Not applicable.<br><br>**Intensity:** Not applicable.
Hong, et al. 2023	***"Home-based exercise program with health education"***<br><br>**Intervention:** Participants completed one-on-one sessions with a physiotherapist to learn the technique of each exercise. In addition, the participants received a video with instructions for each exercise included in the program. Physiotherapists remotely reviewed the participants progress on intervention, adjusted the load of exercises if necessary and provided health education via 3 biweekly health education sessions.<br><br>**Duration:** 6 weeks, 18 sessions, 3 times per week.<br><br>**Intensity:** The load was adjusted every 2 weeks to maintain an effort of perception between 6 and 7 on the OMNI scale.	***"Health education"***<br><br>***Intervention:*** The individuals in the control group attended similar 3 biweekly health education sessions related to protecting their knee joints during activities in daily life. The sessions introduced the basic concept of PFP and the methods used to manage the risks of PFP, but their health education materials did not include exercise information or exercise instructions.<br><br>**Duration:** 6 weeks, 3 sessions.<br><br>**Intensity:** Not applicable.	X
Hott, et al. 2019	***"Hip-focused exercise"***<br><br>**Intervention:** The hip-focused exercises consisted of side-lying hip abduction, hip external rotation and prone hip extension. These exercises were intended to maximally isolate the hip abductors, extensors, and external rotators without stimulating the quadriceps muscles.	***"Knee-focused exercise"***<br><br>**Intervention:** The knee-focused exercise regimens intended to maximally isolate the quadriceps muscles. The exercises consisted of straight-leg raises in the supine position, supine terminal knee extensions, and a mini-squat with the back supported against the wall.	***"Free physical activity"***<br><br>**Intervention:** Patient education to reduce kinesiophobia and encourage self-mastery of symptoms. All patients received standardized oral and written information at the time of inclusion to the study and at randomization. Patients were advised to gradually increase physical activity without excessively provoking knee pain. At

	**Duration:** 6 weeks, 3 times per week.<br><br>**Intensity:** Initial dosage was 3 sets of 10 repetitions for each exercise, with progression to a maximum 20 repetitions. Additional resistance was achieved through weights or elastic tubing depending on the exercise.	**Duration:** 6 weeks, 3 times per week.<br><br>**Intensity:** Initial dosage was 3 sets of 10 repetitions for each exercise, with progression to a maximum 20 repetitions. Additional resistance was achieved through weights or elastic tubing depending on the exercise.	randomization, the control group was encouraged by the study physiotherapist to be physically active.<br><br>**Duration:** Not informed.<br><br>**Intensity:** Not informed.
Ismail, et al. 2013	***"Closed kinetic chain exercises with hip strengthening"***<br><br>**Intervention:** Both groups receveid closed kinetic chain program and instruction for conventional static stretching of hamstring, iliotibial band, gastrocnemius and quadriceps. All subjects were instructed to perform three repetitions of 30-second static stretch of these muscle groups. This group received additional resistive exercises for hip abduction and external rotation.<br><br>**Duration:** 6 weeks, 3 times per week.<br><br>**Intensity:** Each patient was trained at 60% of 10-repetition maximum for hip exercises. A new 10 repetition maximum was established weekly for adjustment.	***"Closed kinetic chain exercises"***<br><br>**Intervention:** The closed kinetic chain program included mini wall squat exercise, forward step up, lateral step up, and terminal knee extension. Conventional static stretching of hamstring, iliotibial band, gastrocnemius and quadriceps. All subjects were instructed to perform three repetitions of 30-second static stretch of these muscle groups.<br><br>**Duration:** 6 weeks, 3 times per week.<br><br>**Intensity:** Not informed.	X
Kamel, et al. 2024	***"Short foot exercise plus hip and knee exercises"***<br><br>**Intervention:** To perform short foot exercise, participants were to elevate the medial longitudinal arch, shorten the foot in the anterior-posterior line, and approximate the first metatarsal head toward the heel without toe flexion. The participants had to start the exercise in a sitting position (in the first and second weeks) and then progress to a double stance (in the third and fourth weeks), then a single-leg stance position (in the fifth and sixth weeks). The hip and knee focused exercises were the same as the other group.<br><br>**Duration:** 6 weeks, 12 sessions, 2 times per week.<br><br>**Intensity:** Resistance was increased using a weight cuff or resistance tubing.	***"Hip and knee exercises"***<br><br>**Intervention:** The hip-focused exercises consisted of side-lying hip abduction, hip external rotation (clamshell), and prone hip extension. The knee-focused exercises consisted of supine straight leg raises, supine terminal knee extensions (from 10° flexion to full extension), and a mini-squat (45° flexion) with the back supported against the wall (to reduce stabilizing requirements from the hip muscles)<br><br>**Duration:** 6 weeks, 12 sessions, 2 times per week.<br><br>**Intensity:** Resistance was increased using a weight cuff or resistance tubing.	X
Keet, et al. 2007	Crossover Design = All participants received medial tape, placebo tape and no tape, in a randomized order. Taping technique and application Patellar tape with the medial glide componente was used in this study. This technique makes use of flexible Fixomull hypoallergenic tape under the rigid leucotape. The placebo patellar tape was Fixomull tape alone and was applied directly over the patella, as above, but without the medial glide component. The tape was applied by an experienced physiotherapist. The order of tape application was randomised. The potential differences between the two taping conditions (placebo and medial patellar tape) were not explained to the subjects.		

Khayambashi, et al. 2012	***"Hip abductor and external rotator strengthening"***<br><br>**Intervention:** Individuals were asked to refrain from exercise-related activity and were allowed to take over-the-counter pain and/or anti-inflammatory medication as needed. Exercises: isolated hip abductor and isolated hip external rotator strengthening.<br><br>**Duration:** 8 weeks, 3 times per week.<br><br>**Intensity:** Resistance and repetitions were progressed at 2-week intervals. Thera-Band elastic tubing was used to provide exercise resistance.	***"Control group"***<br><br>**Intervention:** Patients assigned to the control group were instructed to take 1000 mg of Omega-3 and 400 mg of calcium daily for 8 weeks (placebo intervention). These individuals received no exercise training and were asked to refrain from exercise-related activity and were allowed to take over-the-counter pain and/or anti-inflammatory medication as needed<br><br>**Duration:** 8 weeks, daily.<br><br>**Intensity:** Not applicable.	X
Khojasteh, et al. 2024	***"Physiotherapy plus EMG biofeedback"***<br><br>**Intervention:** This group received conventional treatment plus functional training to activate external rotators and hip abductors, aiming to correct femur and knee alignment during gait initiation and stair descent. Visual EMG biofeedback of gluteal activity was used to enhance muscle contraction during these tasks.<br>**Duration:** 8 weeks, 3 times per week.<br><br>**Intensity:** Not informed.	***"Physiotherapy"***<br><br>**Intervention:** Treatment involved electrotherapy (30 min of conventional TENS at 100 Hz, 50 ms pulse width around the knee), isometric exercises for gluteal and quadriceps muscles, and stretching of quadriceps, iliotibial band, hamstrings, hip adductors, and calf muscles.<br><br>**Duration:** 8 weeks, 3 sessions per week.<br><br>**Intensity:** Not informed.	X
Kısacık, et al. 2021	***"Short Foot exercise"***<br><br>**Intervention:** Aditionally to the treatment program, participants performed short foot exercises, targeting isolated contraction of the plantar intrinsic muscles. To exclude the confounding effects, the patients were asked not to change their shoes and not use orthoses during the treatment.<br><br>**Duration:** 6 weeks, daily<br><br>**Intensity:** Not informed.	***"Control group"***<br><br>**Intervention:** The treatment program of the control group consisted of hip and knee strengthening and stretching exercises, considered as the standard exercise therapy approach.<br><br>**Duration:** 6 weeks, daily<br><br>**Intensity:** Not informed.	X
Kong, et al. 2025	***"Intermittent blood flow-restriction training"***<br><br>**Intervention:** Low-load resistance with blood-flow restriction. Cuff inflated to 80% of indiividualized limb occlusion. Exercises included hip abduction with weights,	***"Placebo blood flow-restriction"***<br><br>**Intervention:** High-load resistance exercise with placebo restriction. Same cuf but inflated to 20 mmHg, insufficient to achieve the effect. Same exercise protocol as the other	X

	clam exercises, bridge, leg press, knee extension and hamstring strecthing.<br><br>**Duration:** 8 weeks, 3 times per week.<br><br>**Intensity:** The limb occlusion pressure is measured to tailor the pressure for each participant, with a retest scheduled at 4 wk. The participants performed exercises with 30% of IRM. 1 set of 30 repetitions followed by 3 sets of 15 repetitions.	group.<br><br>**Duration:** 8 weeks, 3 times per week.<br><br>**Intensity:** 70% of 1RM. 4 sets of 10 repetitions.	
Kowall, et al. 1996	***"Physical therapy program plus patellar taping"***<br><br>**Intervention:** This group did the same therapy program plus taping their knees appropriately before exercise. On initial evaluation, patients in the tape group were to properly tape their knees for home exercises.<br><br>**Duration:** 4 weeks, daily, 30 minutes.<br><br>**Intensity:** Not informed.	***"Physical therapy only"***<br><br>**Intervention:** Extensive stretching and quadriceps muscle-strengthening program. Quadriceps muscle strengthening involved progressive isometric, isotonic and isokinetic exercises. Each group was instructed in a standard home exercise program.<br><br>**Duration:** 4 weeks, daily, 30 minutes.<br><br>**Intensity:** Not informed.	X
Kumar, et al. 2024	***"Positional release of gluteus medius trigger point"***<br><br>**Intervention:** Participants were treated in a prone position with knees flexed. After identifying gluteus medius trigger points, submaximal pressure (~1 kg) was applied, and held for 90 seconds, twice per session. This was followed by therapeutic ultrasound and a 3-minute cold pack on the anterior knee. Strengthening exercises for hip and knee muscles were also prescribed.<br><br>**Duration:** 6 weeks, 18 sessions (2 per week), 45 minutes.<br><br>**Intensity:** Not informed.	***"Myofascial release of gluteus medius trigger point"***<br><br>**Intervention:** In a side-lying position, therapists applied slow, sustained pressure with their knuckle over gluteus medius trigger point until resistance was felt. Upon release in muscle tone, tension was added and the knuckle moved inferiorly for 5–10 minutes. Treatment also included therapeutic ultrasound, a cold pack on the anterior knee and strengthening exercises for hip and knee.<br><br>**Duration:** 6 weeks, 18 sessions (2 per week), 45 minutes.<br><br>**Intensity:** Not informed.	X
Kurt, et al. 2016	***"Kinesiotape"***<br><br>**Intervention:** For the Kinesio tape group: with the knee flexed approximately 90°, Y strips were applied to the quadriceps. Subjects were instructed to avoid taking analgesics or anti-inflammatory medication.	***"Placebo taping"***<br><br>**Intervention:** For the placebo kinesio tape group, 2 strips of tape were applied horizontally with the knee straight, with no tension on the tape. This placement was chosen to avoid interaction of the tape with patellar positioning. Subjects were instructed to avoid taking analgesics or anti-	X

	**Duration:** 2 days.<br><br>**Intensity:** Not applicable.	inflammatory medication.<br><br>**Duration:** 2 days.<br><br>**Intensity:** Not applicable.	
Lee, et al. 2020	***"Dynamic hamstring stretching"***<br><br>**Intervention:** In the supine position, the affected leg was maintained at 90° of flexion of both the hip and knee. The distal thigh was grasped using a towel; then, active knee extension was performed. In the standing position, this exercise was performed by repeated hip flexion with the knee extended. All the participants were treated with the same protocol, including open and closed kinetic chain exercises, balance exercises and core and hip strengthening.<br><br>**Duration:** 12 weeks, daily.<br><br>**Intensity:** Not informed.	***"Static hamstring stretching"***<br><br>**Intervention:** In the sitting or standing position, the affected leg was maintained in a straightened position with ankle dorsiflexion. The foot was grasped using the ipsilateral hand or a towel, with the contralateral hand keeping the affected knee straight, with a slight trunk forward flexion. All the participants were treated with the same rehabilitation protocol except for stretching exercises.<br><br>**Duration:** 12 weeks, daily.<br><br>**Intensity:** Not informed.	X
Lee, et al. 2021	***"Dynamic quadriceps stretching"***<br><br>**Intervention:** In the prone position, the knee joint of the involved leg was flexed maximally, followed by hip extension and hyperextension of the hip joint. In the standing position, the involved leg maintained 90° of knee flexion, followed by continuing with the hip extension motion actively. All the participants were treated with the same rehabilitation protocol, including open and closed kinetic chain exercises, balance exercises and core and hip muscle strengthening.<br><br>**Duration:** 6 months, daily.<br><br>**Intensity:** Not informed.	***"Static quadriceps stretching"***<br><br>**Intervention:** In the prone and standing positions, the involved leg maintained 90° of knee flexion, followed by grasping of the foot using the ipsilateral hand, with the heel of the involved leg slowly pressed on the buttock. All the participants were treated with the same rehabilitation protocol, except for stretching exercises.<br><br>**Duration:** 6 months, daily.<br><br>**Intensity**: Not informed.	X

Lee, et al. 2025	***"Digital therapeutics app"***<br><br>**Intervention:** Structured home-based exercise program incorporating hip and knee targeted muscle strengthening and stretching.<br><br>**Duration:** 8 weeks, daily sessions, 30 minutes per session<br><br>**Intensity:** Adjusted according to the participants' reported pain levels (Borg and NPRS).	***"Conventional treatment"***<br><br>**Intervention:** Disease education materials and 30 minute in-person exercise education session.<br><br>**Duration:** Not informed.<br><br>**Intensity:** Not informed.	X
Lee, et al. 2026	***"Exercise plus blood-flow restriction"***<br><br>**Intervention:** Low-intensity multi-joint exercise protocol. Cuff inflated to 80% of the individual's systolic blood pressure. Exercises included knee extension, hip abdcution, hip external rotation and squat with resistance band.<br><br>**Duration:** 6 weeks, 2 times per week.<br><br>**Intensity:** The resistance band was selected based on a pretest in wich participants rated their perceived exertion for each band using Borg scale (6-20). The initial exercise intensity was set using a color corresponding to the level of 6-8, defined as very light.	***"Exercise only"***<br><br>**Intervention:** Same exercises but without blood-flow restriction.<br><br>6 weeks, 2 times per week.<br><br>**Intensity:** The resistance band was selected based on a pretest in wich participants rated their perceived exertion for each band using Borg scale (6-20). The initial exercise intensity was set using a color corresponding to the level of 6-8, defined as very light.	X
León-Morillas, et al. 2024	***"Standard Physiotherapy Group"***<br><br>**Intervention:** The protocol focused on neuromuscular training to strengthen key muscle groups (quadriceps, hamstrings, hip abductors) for knee stabilization. Proprioceptive exercises were included to improve joint position sense.<br><br>**Duration:** 8 weeks, 2 sessions per week.<br><br>**Intensity:** Not informed.	***"Flossing Band and Physiotherapy Group"***<br><br>**Intervention:** This group followed the same protocol as the Standard Physiotherapy Group, with the addition of a flossing band technique. Before each session, the band was applied around the knee for moderate compression. Exercises were performed as per the previous protocol.<br><br>**Duration:** 8 weeks, 2 sessions per week.<br><br>**Intensity:** Not informed.	X
Liu, et al. 2023	***"Instrument-assisted Soft Tissue Mobilization"***<br><br>**Intervention:** Participants received mobilization treatment in various positions, according to the purpose.<br><br>**Duration:** 4 weeks, 2 sessions per week.	***"Tui-na manipulation"***<br><br>**Intervention:** Tui-na is a relaxation-based therapy combining movement and static manipulation, targeting tendons and bones with gradual progression. The therapy focused on loosening the anterolateral, medial-lateral,	X

	**Intensity:** Not applicable.	posterior, and anterolateral thighs and calves.<br><br>**Duration:** 4 weeks, 2 sessions per week.<br><br>**Intensity:** Not applicable.	
Liu, et al. 2023	***"Instrument-assisted soft tissue mobilization with blood flow restriction"***<br><br>**Intervention:** The experimental group received tissue mobilization combined with blood flow restriction using pressurized equipment. Exercises included seated leg curls, standing leg curls, and weighted hip flexion squats. Blood flow restriction was applied 30 minutes after mobilization, with pressure band placed on the proximal 1/3 of the thigh.<br><br>**Duration:** 4 weeks, 2 sessions per week.<br><br>**Intensity:** Specified according to the exercise, available on table 3 of the article.	***"Instrument-assisted soft tissue mobilization"***<br><br>**Intervention:** Participants received mobilization treatment in various positions, according to the purpose.<br><br>**Duration:** 4 weeks, not informed.<br><br>**Intensity**: Not applicable.	X
Mahmoud, et al. 2015	***"Balance group"***<br><br>**Intervention:** Participants trained balance, in addition to the strengthening, through three stages: static, dynamic and functional balance. Each exercise was repeated 3-5 times.<br>**Duration:** 6 weeks, 3 days per week.<br><br>**Intensity:** The exercise graduated from easy to difficult and the patient didn't progress to a difficult stage until performing the easier one, according to the following protocol shown on table 1 of the article.	***"Control group"***<br><br>**Intervention:** Dynamic strengthening exercises of gluteus medius were done in sidelying or quadruped position, performing hip abduction with external rotation.<br>**Duration:** 6 weeks, 3 days per week.<br><br>**Intensity:** The demand increased by applying an external load using either theraband tied around the thighs, or a soft weight around the ankles. The amount of weight was increased, using the guideline mentioned in the article.	X
Mason, et al. 2011	***"Infrapatellar taping"***<br><br>**Intervention:** In this study, the taping was applied infrapatellar taping only. The taping was applied immediately distal to the patella with the patient in long-sitting and the quadriceps relaxed. Subjects were instructed to keep the tape as dry as possible and to remove the tape if any signs of allergy arose. All subjects were taped when tested at the end of week two.<br><br>**Duration:** 2 week	***"Quadriceps strengthening"***<br><br>**Intervention:** This group did open chain terminal extension quadriceps strenghening. The specific instructions were to observe VMO contractions and avoid pain. The exercises for strengthening were isometric contraction of quadriceps using a towel under knee, towel under heel, heel lifts and low leg lifts.<br><br>**Duration:** 2 weeks, daily, 3 times per day.	"***Quadriceps stretching"***<br><br>**Intervention:** This group did the quadriceps stretching. The stretches were individualized for each patient, so they would not cause knee pain. The instruction was to bring heel towards the same buttock using a strap.<br><br>**Dose:** 2 weeks, daily, 4 times per day.<br><br>**Intensity:** The duration and frequency of the stretching exercises were 10–20 second holds, 3–5 repetitions up to

	**Intensity:** On application, pressure was directed posteriorly and superiorly so as to create a supporting sling for the patella.	**Intensity:** For the strengthening, the patient should hold the contraction for 10 seconds and repeat 10 times.	four times per day.
	**Intervention 4**<br>***“Control group”***<br><br>**Intervention:** The control group received no taping or exercise. All subjects received an overview of knee anatomy and function, and the importance of the quadriceps.<br>*Intervention for the second week:* all subjects followed an identical programme of combined taping, quadriceps strengthening and stretching.<br><br>**Dose:** Not applicable.<br><br>**Intensity:** Not applicable.		
Melo, et al. 2018	***“Kinesio tape with tension”***<br><br>**Intervention:** Participants were positioned with their knee flexed at 90º, then they were submitted to a tape application in Y on the initial medial third área of the thigh, then extending it to the medial edge of the patela.<br><br>**Duration:** 3 days.<br><br>**Intesity:** A 5-cm-wide elastic bandage with 50% tension on the therapeutic zone and 0% tension on the anchors.	***“Kinesio tape without tension”***<br><br>**Intervention:** In this group, the participants were submitted to the application of tape in the same region, but without tension in the bandage and with the limb extended.<br><br>**Duration:** 3 days.<br><br>**Intesity:** KT without tension.	***“Control group”***<br><br>**Intervention:** Participants did not perform the protocol and remained at rest for 5 minutes (time required for the technique application in the other groups).<br><br>**Duration:** NA.<br><br>**Intesity:** NA.
Melo, et al. 2024	**“Exercise combined with NMES”**<br><br>**Intervention:** Both groups followed a lower limb exercise routine targeting knee extensors and hip abductors, organized into three levels based on movement difficulty, external resistance, and electrical current intensity. This group also received NMES, with the proximal electrode placed on the lower third of the anteromedial thigh and the distal electrode 3 cm above the superomedial patella to stimulate the vastus medialis oblique. For the gluteus medius, the proximal electrode was below the iliac crest and the distal electrode above the greater trochanter.<br><br>**Duration:** 8 weeks, 16 sessions (2 per week).<br><br>**Intensity:** Exercise intensity was consistent with the other group. NMES parameters included rectangular, biphasic,	***“Exercise only”***<br><br>**Intervention:** Both groups followed a lower limb exercise routine targeting knee extensors and hip abductors, organized into three levels based on movement difficulty, external resistance, and electric current intensity.<br><br>**Duration:** 8 weeks, 16 sessions (2 per week).<br><br>**Intensity:** At level 1, participants used a light elastic band and 1 kg shin guard for 12–15 repetitions based on the Borg scale. In level 2, a moderate elastic band, 2 kg ankle weights, and 2 kg dumbbells were used, with 10–12 repetitions based on the Borg scale. In level 3, a strong elastic band, 3 kg ankle weights, and 3 kg dumbbells were applied, with 10–12 repetitions. Two sets were performed per exercise, with a 60-second rest between sets and	X

	symmetrical pulse shape, 200 µs pulse duration, 10 ms interburst interval, and 2,500 Hz modulated in 50 bursts. The Ton:Toff ratio was set to 1:1, with 1-second ascent and descent ramps.	exercises.	
Nakagawa, et al. 2008	***"Strengthening of quadriceps plus strengthening of hip abductor and lateral rotator muscles"***<br><br>**Intervention:** The intervention group received the same exercise protocol as the control group as well as additional time for strengthening and functional training exercises focused on the transversus abdominis muscle, hip abductors and lateral rotator muscles.<br><br>**Duration:** 6 weeks, 5 times per week.<br><br>**Intensity:** Specified for each exercise on article appendix.	***"Control group"***<br><br>**Intervention:** The exercise protocol for the control group consisted of patellar mobilization, stretching of the quadriceps, gastrocnemius, iliotibial band and hamstrings and open and closed kinetic chain exercises for quadriceps strengthening.<br><br>**Duration:** 6 weeks, 5 times per week.<br><br>**Intensity:** Specified for each exercise on article appendix.	X
Ozlu, et al. 2024	***"High intensity laser"***<br><br>**Intervention:** The exercise program included stretching and strengthening exercises, with new exercises added weekly. The laser was used, applied directly to the skin on the patellar margins with circular motions.<br><br>**Duration:** 2 weeks, 10 sessions (5 per week).<br>**Intensity:** Exercises were performed as 1 set of 10 repetitions, 3 sets per day during treatment, and continued as a home program for 3 months. Laser parameters included a frequency of 25 Hz at 10 watts with a dosage of 12 J/cm² for 4 minutes in analgesia mode, and 150 J/cm² with 7 watts of power for 6 minutes in continuous mode for biostimulation.	***"Ultrasound and TENS"***<br><br>**Intervention:** All groups followed the same exercise program. Participants were positioned supine with the knee extended. Ultrasound (US) was applied around the patella using circular movements, while TENS was applied using four adhesive electrodes on the medial and lateral knee joint.<br>**Duration:** 2 weeks, 10 sessions (5 per week).<br><br>**Intensity:** Ultrasound was applied at 1.5 W/cm², 1 MHz, 50% intermittent for 5 minutes. TENS was set to 80 Hz frequency and 50–100 ms pulse duration for 20 minutes, with current intensity adjusted to the patient's tolerance.<br>.	***"Ultrasound and interferential"***<br><br>**Intervention:** All groups followed the same exercise program. Participants were positioned supine with the knee extended. Ultrasound was applied around the patella using circular movements. Interferential current was applied with four vacuum probes placed on the medial and lateral patella.<br>**Duration:** 2 weeks, 10 sessions (5 per week).<br><br>**Intensity:** Ultrasound was applied at 1.5 W/cm², 1 MHz, 50% intermittent for 5 minutes. Interferential current was applied at 4 Hz frequency, 90–100 Hz pulse frequency, and a 1/1 rectangular spectrum for 20 minutes.

Pompeo, et al. 2022	***"Standard Rehabilitation Group"***<br><br>**Intervention:** The standard exercise program included exercises targeting core, hip abductors, hip external rotators and hip extensor muscles, in addition to traditional hip and knee exercises.<br><br>**Duration:** 12 weeks, 24 sessions**,** 2 times a week, 60-90min in each training session.<br><br>**Intensity:** Varied accordingly to the exercise type.	***"Distal Exercise Group"***<br><br>**Intervention:** The distal exercise program included exercises targeting foot inversors, ankle plantar flexors, ankle dorsiflexors, and intrinsic foot muscles, in addition to traditional hip and knee exercises.<br><br>**Duration:** 12 weeks, 24 sessions**,** 2 times a week, 60-90min in each training session.<br><br>**Intensity/dose: V**aried accordingly to the exercise type.	X
Qureshi, et al. 2025	***"Dry needling plus conventional physiotherapy"***<br><br>**Intervention:** Dry needling was applied to trigger points on the quadriceps femoris muscles. Conventional physiotherapy included hot pack, strengthening and stretching of quadriceps and hamstring muscles.<br><br>**Duration:** 4 weeks, 2 sessions per week, 30 minutes per session of dry needling and 30 minutes of exercise.<br><br>**Intensity:** Not informed.	***"Conventional physiotherapy"***<br><br>**Intervention:** Conventional physiotherapy included hot pack, strengthening and stretching of quadriceps and hamstring muscles.<br><br>**Duration:** 4 weeks, 2 sessions per week, 30 minutes per session of dry needling and 30 minutes of exercise.<br><br>**Intensity:** Not informed.	X
Rabelo, et al. 2017	***"Movement Control & Strengthening"***<br><br>**Intervention:** Same strengthening program as the other group, but were informed about movement control disorders common in women with PFP and were instructed to correct these during the execution of the exercises and during daily living activities<br><br>**Duration:** 4 weeks, 12 sessions**,** 3 sessions per week, 40-60 minutes.<br><br>**Intensity:** 70% of 1-RM. Exercises using elastic resistance were standardized for the maximum load that each patient could support while completing 15 repetitions of the exercise.	***"Strengthening"***<br><br>**Intervention:** Set of conventional weight-bearing and non-weight-bearing exercises emphasizing knee extensor, abductor, and lateral rotator hip strengthening.<br><br>**Duration:** 4 weeks, 12 sessions**,** 3 sessions per week**,** 40-60 minutes.<br><br>**Intensity:** 70% of 1-RM. Exercises using elastic resistance were standardized for the maximum load that each patient could support while completing 15 repetitions of the exercise. Non-weight-bearing exercises were initiated using ankle weights and elastic bands and progressed to a machine.	X
Riel, et al. 2017	***"Feedback group"***<br><br>**Intervention:** This group was provided with visual and auditory feedback on contraction time and pulling force.	***"Control group"***<br><br>**Intervention:** Control group was only provided with real-time feedback on pulling force. Adolescents were	X

	They were instructed to perform three elastic band exercises: seated knee extension and freestanding hip abduction and extension.<br><br>**Duration:** 6 weeks, 3 times per week.<br><br>**Intensity:** Each adolescent's 10–12 repetition maximum was determined by shortening the elastic band to a length where they felt that they would not be able to perform more than 10 repetitions.	instructed to perform three elastic band exercises: seated knee extension and freestanding hip abduction and extension.<br><br>**Duration:** 6 weeks, 3 times per week.<br><br>**Intensity:** Each adolescent's 10–12 repetition maximum was determined by shortening the elastic band to a length where they felt that they would not be able to perform more than 10 repetitions.	
Rodrigues, et al. 2021	***"Strengthening plus anodic transcutaneal stimulation"***<br><br>**Intervention:** In this group, subjects received a stimulus of 2 mA for 20 minutes over the motor cortex. The stimulus was applied preceding knee extension exercise in a leg extension at 60% of 10RM with a 1-minute interval between sets.<br><br>**Duration:** 12 sessions, 2-3 times per week.<br><br>**Intensity:** Subjects were submitted to a 10RM test and retest to stabilize the load on the tests. The highest load obtained between tests was considered for the training sessions. The RT protocol consisted of 3 sets of 12 repetitions.	***"Strengthening plus sham stimulation"***<br><br>**Intervention:** In the Sham condition, subjects received the same 2-mA stimulus on the motor cortex, but for only 30 seconds. In both conditions, the stimulus was applied preceding the reabilitation treatment.<br><br>**Duration:** 12 sessions, 2-3 times per week.<br><br>**Intensity:** Subjects were submitted to a 10RM test and retest to stabilize the load on the tests. The highest load obtained between tests was considered for the training sessions. The RT protocol consisted of 3 sets of 12 repetitions.	X
Roh, et al. 2021	**"Electro-cryotherapy plus exercise"**<br><br>**Intervention:** Patients received a 30-min simultaneous application of TENS and knee joint cooling on the involved leg. The exercise program contained warming-up, stretching, strengthening, and balancing exercises.<br><br>**Duration:** 4 weeks, 10 sessions.<br><br>**Intensity:** TENS intensity was set at the highest amplitude that could be reached without any muscle contraction or subjective uncomfortable sensations noted. The exercise intensity (external load) was determined by the DAPRE technique (table 2 on the article).	***"Exercise alone"***<br><br>**Intervention:** The exercise program contained warming-up, stretching, strengthening, and balancing exercises.<br><br>**Duration:** 4 weeks, 10 sessions.<br><br>**Intensity:** The exercise intensity (external load) was determined by the DAPRE technique (table 2 on the article).	X

Saad, et al. 2018	***"Hip exercises"***<br><br>**Intervention:** This group performed exercises to strengthen hip stabilizing muscles.<br><br>**Duration:** 8 weeks, 2 times per week, 50 minutes per session.<br><br>**Intensity:** Weights were increasing as the patient's reported changes in their following the Rating of Perceived Exertion scale based on Borg's Scale of Effort.	***"Quadriceps exercises"***<br><br>**Intervention:** The exercises in this group focused specifically on quadriceps strengthening.<br><br>**Duration:** 8 weeks, 2 times per week, 50 minutes per session.<br><br>**Intensity:** Weights were increasing as the patient's reported changes in their following the Rating of Perceived Exertion scale based on Borg's Scale of Effort.	***"Stretching exercises"***<br><br>**Intervention:** The physical therapist monitored and stabilized the patients during the stretching exercises.<br><br>**Duration:** 8 weeks, 2 times per week, 50 minutes per session.<br><br>**Intensity:** Weights were increasing as the patient's reported changes in their following the Rating of Perceived Exertion scale based on Borg's Scale of Effort.
	**Intervention 4**<br>***"Control group"***<br><br>**Intervention:** Patients included in this group did not have any kind of intervention for eight weeks, but they were tested at the start of the program & at the end like the other 3 groups. At the end of the experiment, a rehabilitation program was made available to all patients.		
Şahin, et al. 2016	***"Hip plus knee exercises"***<br><br>**Intervention:** The same exercises as the knee group, plus hip abductor and external rotator strengthening exercises. Patients were asked to hold the muscles in contraction for 3.5s in each exercise.<br><br>**Duration:** 6 weeks, 30 sessions, 5 sessions per week; plus 6 weeks of home exercise.<br><br>**Intensity:** Variable to each exercise. Exercises utilizing elastic resistance were standardized to the maximum resistance at which was able to perform 10 repetitions.	***"Knee exercises only"***<br><br>**Intervention:** Exercises: lower extremity stretches, knee extensor strengthening, straight leg raise and minisquatting. Both groups received patient education.<br><br>**Duration:** 6 weeks, 30 sessions, 5 sessions per week; plus 6 weeks of home exercise.<br><br>**Intensity:** Variable to each exercise. Exercises utilizing elastic resistance were standardized to the maximum resistance at which each patient was able to perform 10 repetitions of the exercise.	X
Scafoglieri, et al. 2021	***"Local exercise"***<br><br>**Intervention:** This group focused on strengthening knee and hip muscles. The exercises were supplemented with mobilisations of the patellofemoral joint.<br><br>**Duration:** 6 weeks, 3 times per week.<br><br>**Intesity:** The physiotherapist gradually increased the intensity of the exercises improving muscle endurance.	***"Spinal manual therapy"***<br><br>**Intervention:** Manipulation of a joint was conducted if a movement impairment was found in any of the regions. Manipulations were only provided in case of a clinical indication. Patients were also asked to do home exercises focusing on mobilizing the thoracolumbar region.<br><br>**Duration:** 6 weeks, once a week.<br><br>**Intensity:** Not applicable.	X

Shahnaz, et al. 2012	***"EMG-biofeedback guided isometric exercise"***<br><br>**Intervention:** Both groups received paraffin wax bath (temperature 52ºC) for 20 minute prior to exercise. Three sets of exercise were given: isometric quadriceps exercise, terminal knee extension and straight leg raising. The participants were asked to watch the muscle activity and try to increase the activity level of vastus medialis oblique and vastus lateralis while performing the exercises.<br><br>**Duration:** 3 weeks, 5 days a week.<br><br>**Intensity:** Not informed.	***"Sham EMG-biofeedback with isometric exercise"***<br><br>**Intervention:** Paraffin wax bath for 20 minutes. Same set of exercise as the other group but the electrodes was placed away from the quadriceps vastus, and reference electrode was placed below the tibial tuberosity.<br><br>**Duration:** 3 weeks, 5 days a week.<br><br>**Intensity:** Not informed.	X
Sheikhi, et al. 2024	***"Education plus trunk and hip exercises"***<br><br>**Intervention:** Patients in the experimental group received three supervised educational sessions, each lasting 30 to 45 minutes. These sessions were personalized based on the patient's characteristics, focusing on understanding pain and treatment expectations. Additionally, they followed the same exercise program as the control group.<br><br>**Duration:** 8 weeks, 3 sessions per week, 60 minutes per session.<br><br>**Intensity:** The exercises were divided in three parts: first part focused on motor control, second aimed to increase strength and third involved weight-bearing exercises, but they did not inform how they increased load.	***"Trunk and hip exercises"***<br><br>**Intervention:** All patients participated in 6 group-based classes, supervised by a physical therapist. The control group trained on even days.<br><br>**Duration:** 8 weeks, 3 sessions per week, 60 minutes per session.<br><br>**Intensity:** The exercises were divided in three parts: first part focused on motor control, second aimed to increase strength and third involved weight-bearing exercises, but they did not inform how they increased load.	X
Silva, et al. 2023	***"Strengthening Group"***<br><br>**Intervention:** The participants were submitted to strengthening exercises that focused on the core, lateral rotator, abductor, hip extensor, and quadriceps muscles.<br><br>**Duration:** 12 weeks, 24 sessions, 2 times a week, 60 minutes per session.<br><br>**Intensity:** The initial load was based on 70% of 1-RM. The load was increased 5–10% weekly, from the 3rd week of treatment.	***"Neuromuscular Training Group"***<br><br>**Intervention:** This group performed the same strengthening protocol, with neuromuscular exercises on stable and unstable surfaces, from the fourth week onwards.<br><br>**Duration:** 12 weeks, 24 sessions, 2 times a week, 80 minutes per session.<br><br>**Intensity:** The initial load was based on 70% of 1-RM. The load was increased 5–10% weekly, from the 3rd week of treatment.	X

Steinberg, et al. 2020	***"Somatosensory"***<br><br>**Intervention:** The program included weight-bearing exercises that emphasized muscle strengthening and postural balance/proprioceptive stimulations.<br><br>**Duration:** 12 weeks, 2 times a week, 15 minutes per session.<br><br>**Intensity:** All exercises were performed from the easiest to the most difficult. The exercises were performed on both hard and unstable surfaces. After practicing the exercise with open eyes, the exercises were performed with closed eyes.	***"Isometric exercises"***<br><br>**Intervention:** The program included isometric exercises with a straight leg raised in the air in static form to strengthen groups of muscles around the knee and around the hip.<br><br>**Duration:** 12 weeks, 2 times a week, 15 minutes per session.<br><br>**Intensity:** To raise the level of the exercises: the number of seconds for each set was increased each week; number of repetitions was increased; ankle weight of 0.5kg for each leg for the exercise; different variations, with additional isometric exercises included for variation.	***"Control group"***<br><br>**Intervention:** This group did an upper-body stretching program. The exercises were performed in both standing and lying positions.<br><br>**Duration:** 12 weeks, 2 times a week, 15 minutes per session.<br><br>**Intensity:** 15 repetitions.
Talbot, et al. 2020	***"NMES with home exercise"***<br><br>**Intervention:** The NMES protocol consisted of 20 min of NMES stimulation while concurrently performing the HEP.<br><br>**Duration:** 9 weeks, 31 sessions.<br><br>**Intensity:** Stimulation intensity was based on calculated percentages of maximal voluntary contraction (MVC). Participants were trained at 20–30% of MVC during weeks 1–3, 31–40% weeks 4–6, and 41–50% weeks 7–9.	***"TENS with home exercise"***<br><br>**Intervention:** The TENS group used a battery-operated KneeHAB XP controller with lead wire TENS applicator. The TENS protocol consisted of 20 min of TENS stimulation performed with HEP.<br><br>**Duration:** 9 weeks, 31 sessions.<br><br>**Intensity:** Parameters were a pulse duration of 200 µs with a frequency of 99 Hz. Electrode placement consisted of a crisscross technique around the knee.	***"NMES+TENS with home exercise"***<br><br>**Intervention:** The group receiving all three treatments had electrical stimulation via the KneeHAB XP controller following the protocols described in the other groups. NMES and TENS were performed on alternating days concurrently with HEP. This group also used daily email logs and received follow-up contact from study staff.<br><br>**Duration:** 9 weeks, 31 sessions.<br><br>**Intensity:** The same as the other groups.
	**Intervention 4**<br>***"Home exercise only"***<br><br>**Intervention:** All participants received a home exercise program (HEP), which teaches quadriceps muscle strengthening exercises and self-anagement to promote adherence. The exercises consist of straight leg raises, quad sets, step-ups, and squats.<br><br>**Duration:** 9 weeks, 31 sessions.<br><br>**Intensity:** NI.		
Talbot, et al. 2023	***"NMES plus high blood flow restriction"***<br><br>**Intervention:** The NMES protocol consisted of 20 minutes of stimulation while performing active exercises. A tourniquet system applied high blood flow restriction, with	***"NMES plus low blood flow restriction"***<br><br>**Intervention:** Participants in the low blood flow restriction group underwent the same program as the previous one, with the cuff inflated to 20mmHg while performing	X

	four 5-minute cuff inflations at 80% limb occlusion pressure, separated by 1-minute rests, while simultaneously performing NMES and exercises. The sequence of exercises began with stretching the quadriceps and hamstring and then a combination of open and closed chain exercises including active straight leg raises, quadriceps straightening, step-up, and squats.<br><br>**Duration:** 9 weeks.<br><br>**Intensity:** The stimulation intensity was set as a percentage of maximal voluntary contraction (% MVC) based on strength results. Participants trained at 20-30% MVC during weeks 1-3, 31-40% MVC during weeks 3-6, and 41-50% MVC during weeks 6-9, with adjustments made during clinic visits to maintain appropriate intensity.	exercises. The sequence of exercises began with stretching the quadriceps and hamstring and then a combination of open and closed chain exercises including active straight leg raises, quadriceps straightening, step-up, and squats.<br><br>**Duration:** 9 weeks.<br><br>**Intensity:** The stimulation intensity was set as a percentage of maximal voluntary contraction (% MVC) based on strength results. Participants trained at 20-30% MVC during weeks 1-3, 31-40% MVC during weeks 3-6, and 41-50% MVC during weeks 6-9. Adjustments were made during clinic visits to maintain appropriate intensity.	
Thomée 1997	***"Isometric muscle contractions"***<br><br>**Intervention:** Educational component and a training program. This group performed four different straight leg raises, toe raises on one and both feet, exercises in standing position (hip flexion, extension, adduction and abduction with a rubber tule), exercise for lower-extremity balance and stationary bicycle.<br><br>**Duration:** 12 weeks, 3 times per week.<br><br>**Intensity:** The progression of exercises and loads were individually prescribed using the progression plan, the pain monitoring system and the diary.	***"Eccentric muscle contractions"***<br><br>**Intervention:** Educational component and a training program. This group performed two different leg-lowering exercises, eccentric contractions in a sitting position, step-down and one-legged squat using eccentric contractions, lower-extremity balance and stationary bicycle.<br><br>**Duration:** 12 weeks, 3 times per week.<br><br>**Intensity:** The progression of exercises and loads were individually prescribed using the progression plan, the pain monitoring system and the diary.	X
Vasconcelos, et al. 2024	***"Strength and power training"***<br><br>**Intervention:** The program followed the same structure as the strength training group but included exercises for muscle power. Participants were instructed to perform the concentric phase of power exercises as quickly as possible.<br><br>**Duration:** 12 weeks, 3 sessions per week, 75 minutes per session.<br><br>**Intensity:** Participants used ankle weights, free weights, dumbbells, and/or elastic bands. The initial focus (first 2	***"Strength training"***<br><br>**Intervention:** This program focused on progressive resistance exercises to build strength. The first two weeks aimed at developing neuromotor control and muscle endurance (load < 50% 1RM). From weeks 3 to 12, the goal was to develop muscle strength (load > 70% 1RM).<br><br>**Duration:** 12 weeks, 3 sessions per week, 60 minutes per session.<br><br>**Intensity:** Participants used ankle weights, free weights,	X

	weeks) was on neuromotor control and muscle endurance development (load < 50% 1RM). From weeks 3 to 12, the focus shifted to developing strength (load > 70% 1RM) and power (load between 40-60% 1RM).	dumbbells, and/or elastic bands. The first two weeks aimed at developing neuromotor control and muscle endurance (load < 50% 1RM). From weeks 3 to 12, the goal was to develop muscle strength (load > 70% 1RM).	
Witvrouw, et al. 2000	***"Open kinetic chain"***<br><br>**Intervention:** Consisted of maximal static quadriceps muscle contractions with the knee in full extension; straight-leg raises with the patient supine; short arc movements from 10° of knee flexion to terminal extension; and leg adduction exercises in the lateral decubitus position. The patients were instructed to perform the conventional static quadriceps, hamstring, and gastrocnemius muscle stretching exercises.<br><br>**Duration:** 5 weeks, 3 times per week, 30 to 45 minutes per session.<br><br>**Intensity:** Before the beginning of the program, a 10-RM was determined. Patients were instructed to train at 60% of their maximum. A new 10-RM was established at the end of a week of training.	***"Closed kinetic chain"***<br><br>**Intervention:** Consisted of seated leg presses; one-third knee bends on one leg and on both legs; stationary bicycling; rowing-machine exercises; step-up and step-down exercises; and progressive jumping exercises. The patients were instructed to perform the conventional static quadriceps, hamstring, and gastrocnemius muscle stretching exercises after each training session.<br><br>**Duration:** 5 weeks, 3 times per week, 30 to 45 minutes per session.<br><br>**Intensity:** Before the beginning of the program, a 10-RM was determined. Patients were instructed to train at 60% of their maximum. A new 10-RM was established at the end of a week of training.	X
Won, et al. 2025	***"Lumbar spinal manipulation"***<br><br>**Intervention:** Participants were positioned in side-lying with the painful knee upward. The therapist guided the upper leg into 90◦ hip flexion, resting the foot behind the contralateral knee. A high-velocity, low-amplitude thrust was delivered following preloading into the rotational barrier at end range.<br><br>**Duration:** 4 weeks, 2 times per week.<br><br>**Intensity:** Not applicable.	***"Placebo manipulation"***<br><br>**Intervention:** The placebo procedure mimicked the setup and therapist contact used in the manipulation group, including the same hand placements and positioning. However, no thrust or end-range movement was applied; instead, the final position was held for 5 s to simulate therapeutic intent while.<br><br>**Duration:** 4 weeks, 2 times per week.<br><br>**Intensity:** Not applicable.	X
Yalfani, et al. 2025	***"Forefoot strike pattern plus strengthening exercises"***<br><br>**Intervention:** The rehabilitation program included foot-knee exercises and gait retraining based on the forefoot strike pattern. The foot exercises included posterior tibia strengthening, heel raises, and short foot. The knee exercises consisted of straight-leg raises, supine terminal	***"Control Group"***<br><br>The control group did not receive any intervention.	X

	knee extensions, and a mini-squat. Foot strike pattern included verbal instructions and feedback with mirror.<br><br>**Duration:** 8 weeks, 3 times per week, 15-30 minutes of forefoot strike pattern and 60 minutes of exercise<br><br>**Intensity:** Not informed.		
Yang, et al. 2026	***"Eletrical stimulation plus strength traning"***<br><br>**Intervention:** Exercises included knee extension, wall squat, clamshell hip abduction, bridge, squat and hip thrust with barbell. Warm-up and stretching were also performed. In this group, during the strength training, eletrical stimulation was applied via a constant current stimulator.<br><br>**Duration:** 6 weeks, 3 sessions per week, 60 minutes per session.<br><br>**Intensity:** 70% of 1RM. Eletrical stimulation intensity was individualized to each participant to elicit maximal vastus medialis contraction while minimizing discomfort.	***"Strength traning"***<br><br>**Intervention:** Exercises included knee extension, wall squat, clamshell hip abduction, bridge, squat and hip thrust with barbell. Warm-up and stretching were also performed.<br><br>**Duration:** 6 weeks, 3 sessions per week, 60 minutes per session.<br><br>**Intensity:** 70% of 1RM.	X
Yelvar, et al. 2015	***"Knee and postural control exercises"***<br><br>**Intervention:** The principles of postural stabilization were explained before the treatment and patients were asked to comply with these during the exercise. Stabilization exercises were done with diaphragmatic breathing. After instruction on the principles, the exercise program was started. Exercises are available on table 1 of the article.<br><br>**Duration:** The stabilization exercises were performed 3 days a week for 6 week. Knee exercises were done 3 times per day during 6 weeks.<br><br>**Intensity:** First two weeks, exercise using their own body weight. During the next 4 weeks, in order to increase force, an appropriate elastic resistance band for the patient was chosen and exercises were done with it. Not informed how the elastic band was chosen.	***"Knee exercises only"***<br><br>**Intervention:** Patients were prescribed only the therapeutic knee exercise program at home and asked to write an exercise diary for completed exercises. They were encouraged to do these exercises correctly and consistently by being invited to the clinic every week and contacted by telephone. Exercises are available on table 1 of the article.<br><br>**Duration:** 6 weeks, 3 times per day.<br><br>**Intensity:** First two weeks, exercise using their own body weight. During the next 4 weeks, in order to increase force, an appropriate elastic resistance band for the patient was chosen and exercises were done with it. Not informed how the elastic band was chosen.	X

Yip, et al. 2006	***"EMG biofeedback plus exercise"***<br><br>**Intervention:** The subjects were given a EMG biofeedback machine, able to provide real-time normalized visual biofeedback signals according to the intensity of contraction of the muscles. The participants were asked to selectively increase the activity of vastus medialis obliquus while maintaining a relatively stable activity in vastus lateralis during the exercises. Exercise protocol is the same as the other group.<br><br>**Duration:** 8 weeks, 7 sessions per week, 15 minutes per session.<br><br>**Instensity:** Not informed.	***"Exercise only"***<br><br>**Intervention:** The exercise protocol composed flexibility exercises (stretching of lower limb muscles including quadriceps, hamstring, gastrocnemius, hip adductors and iliotibial band; and mobilization to the patella), strengthening exercises for the quadriceps with emphasis on vastus medialis obliquus recruitment (quadríceps set, terminal knee extension, semi-squatting, wall slide, lunge, step-up, step-down, eccentric hamstring exercise, hip adduction exercise), balance and proprioception training, pylometric and agility training.<br><br>**Duration:** 8 weeks, 7 sessions per week, 15 minutes per session.<br><br>**Intensity:** Not informed.	X

Abbreviations: EMG-BF: Electromyographic Biofeedback; NMES: Neuromuscular Electrical Stimulation; RM: Repetition Maximum; PENS: Percutaneous Electrical Nerve Stimulation; TENS: Transcutaneous Electrical Nerve Stimulation.

Supplementary Material G. Risk of bias for the included studies using the RoB 2.0.

Study	1	S	2	3	4	5	6 (Overall)
Abdelmohsen et al. 2025							High
Abdo et al. 2025							High
Aghakeshizadeh et al. 2021							Low
Ahmadi, et al. 2020							High
Almeida et al. 2021							Low
Alonazi et al. 2021							High
Arslan et al. 2023							High
Asif et al. 2022							High
Aytar et al. 2011							High
Azab et al. 2022							Low
Baldon et al. 2014							Low
Basbug et al. 2021							High
Bily et al. 2008							High
Boitrago et al. 2021							High
Callaghan et al. 2001							High
Callaghan et al. 2004							High
Celik et al. 2019							High
Cho et al. 2025							High
Clark et al. 2000							Concerns
Constantinou et al. 2022							High
Corum et al. 2018							High
Dolak et al. 2011							Concerns
Drew et al. 2017							High
Emamvirdi et al. 2019							High
Esculier et al. 2017							Low
Ferber et al. 2015							Concerns
Giles et al. 2017							Low
Girardi et al. 2022							High
Glaviano et al. 2019							Low
Hammami et al. 2024							High
Hansen et al. 2023							Low
Hasan et al. 2022							High
Hasan et al. 2025							High
Herrington et al. 2007							High
Hong et al. 2023							High
Hott et al. 2019							Low
Ismail et al. 2013							Concerns
Kamel et al. 2024							High
Keet et al. 2007							High
Khayambashi et al. 2012							High
Khojasteh et al. 2024							High
Klsaclk et al. 2021							High
Kong et al. 2025							High
Kowall et al. 1996							High
Kumar et al. 2024							High
Kurt et al. 2016							High
Lee et al. 2020							High
Lee at al. 2021							High
Lee at al. 2025							High

Domains

1. Randomization process
S. Period and carryover effects (for crossover)
2. Deviations from intended interventions
3. Missing outcome data
4. Measurement of the outcome
5. Selection of the reported result
6. Overall risk of bias

- *Green shading*: indicates low risk of bias
- *Yellow shading*: indicates some concerns
- *Red shading*: indicates high risk of bias
- *Black shading*: not applicable

Lee at al. 2026							High
León-Morillas et al. 2024							High
Liu and Wang 2023							High
Liu and Wu 2023							High
Mahmoud et al. 2015							High
Mason et al. 2011							High
Melo et al. 2018							High
Melo et al. 2024							Low
Nakagawa et al. 2008							Concerns
Ozlu et al. 2024							High
Pompeo et al. 2022							Concerns
Qureshi et al. 2025							High
Rabelo et al. 2017							Low
Riel et al. 2017							High
Rodrigues et al. 2021							High
Roh et al. 2021							High
Saad et al. 2018							Concerns
Sahin et al. 2015							High
Scafoglieri et al. 2021							High
Shahnaz et al. 2012							High
Sheikhi et al. 2024							Low
Silva et al. 2023							Low
Steinberg et al. 2020							High
Talbot et al. 2020							High
Talbot et al. 2023							High
Thomeé et al. 1997							High
Vasconcelos et al. 2024							Concerns
Witvrouw et al. 2000							High
Won et al. 2025							High
Yalfani et al. 2025							High
Yang et al. 2026							High
Yelvar et al. 2014							High
Yip et al. 2006							High

Supplementary Material H. Individual data of the included studies [mean (SD)].

Study	Results				
	**Variables**	**Weight-bearing exercise (n=15)**		**Control group (n=15)**	
		**Pre (Baseline)**	**Post (8 weeks)**	**Pre (Baseline)**	**Post (8 weeks)**
Abdelmohsen, et al. 2025	VAS (cm)	6.19 (1.68)	3.13 (1.41)	5.21 (1.48)	4.93 (1.27)
	PPT	2.69 (0.62)	3.59 (1.03)	2.78 (0.73)	3.13 (0.66)
	Oswestry Disability Index (%)	58.38 (9.64)	42.75 (11.02)	51.71 (8.87)	53.86 (8.44)
	Hip flexion 60º/s	43.44 (17.74)	81.06 (27.98)	42.80 (12.62)	49.66 (11.26)
	Hip extension 60º/s	29.70 (12.27)	47.50 (21.01)	38.08 (45.28)	45.28 (16.33)
	Hip abduction 60º/s	28.61 (14.83)	42.53 (21.35)	41.14 (21.12)	34.11 (15.54)
	Hip adduction 60º/s	23.39 (12.93)	54.64 (21.50)	25.56 (12.71)	20.61 (8.87)
	Knee flexion 60º/s	18.71 (5.00)	36.64 (8.42)	20.86 (5.72)	24.00 (5.28)
	Knee extension 60º/s	51.79 (9.61)	123.12 (41.65)	63.53 (20.70)	67.56 (20.51)
	Concentric strength muscle assessment using an isokinetic dynamometer.				
	**Variables**	**Exercise + Manual therapy (n=25)**		**Exercise only (n=28)**	
		**Pre (Baseline)**	**Post (4 weeks)**	**Pre (Baseline)**	**Post (4 weeks)**
Abdo, et al. 2025	VAS - Ascending (cm)	50.68 (23.43)	20.96 (17.16)	50.39 (25.42)	23.25 (18.12)
	VAS - Descending (cm)	38.28 (24.75)	15.52 (17.12)	40.93 (23.36)	17.64 (17.79)
	VAS - Squatting (cm)[!]	64.28 (28.59)	23.96 (18.56)	53.43 (26.88)	26.89 (22.31)
	VAS - Sittng (cm)	57.16 (31.34)	27.68 (25.52)	58.54 (31.55)	32.86 (24.22)
	AKPS (points)	67.08 (13.56)	83.84 (10.63)	70.86 (13.76)	83.75 (9.50)
	Step-down test	13 (4)	16 (4)	12 (5)	15 (5)
	Hip abduction (%BW)	13.15 (4.46)	16.08 (5.39)	14.83 (5.66)	17.51 (4.88)
	Hip external rotation (%BW)	12.01 (4.72)	13.88 (6.38)	13.34 (5.02)	15.28 (4.93)
	Knee extension (%BW)	25.74 (11.22)	30.28 (12.01)	23.81 (9.76)	31.41 (12.07)
	Isometric strength muscle assessment using handheld dynamometer.				

Aghakeshizadeh, et al. 2021

Variables	Internal Focus (n=23)		External Focus (n=23)		Control (n=24)	
	Pre (Baseline)	Post (6 weeks)	Pre (Baseline)	Post (6 weeks)	Pre (Baseline)	Post (6 weeks)
VAS (points)	7.0 (1.7)	2.7 (1.2)	7.6 (1.4)	2.1 (0.8)	6.5 (2.7)	4.2 (0.9)
AKPS (points)	70.6 (4.3)	80.0 (8.9)	65.4 (3.8)	85.6 (7.0)	68.4 (5.0)	74.2 (7.9)
Hip abduction (%BW)	13.3 (4.8)	18.3 (3.1)	11.9 (4.7)	21.1 (6.2)	14.0 (6.0)	17.5 (4.4)
Hip external rotation (%BW)	9.2 (1.2)	13.5 (2.1)	10.1 (2.1)	16.1 (2.3)	7.8 (1.0)	10.1 (3.0)

Isometric strength muscle assessment using handheld dynamometer.

Ahmadi, et al. 2020

Variables	Neurofeedback training (n=16)		Control group (n=16)	
	Pre (Baseline)	Post (12 weeks)	Pre (Baseline)	Post (12 weeks)
VAS (cm)	5.66 (1.39)	4.13 (1.18)	7.00 (1.85)	6.53 (1.35)
Hip abduction	30.21 (4.20)	28.22 (3.10)	32.08 (7.41)	29.48 (3.92)
Knee extension	36.91 (5.69)	36.98 (6.17)	35.83 (9.67)	33.41 (8.75)

Isometric strength muscle assessment using handheld dynamometer.

Almeida, et al. 2021

Variables	Anteromedial hip strengthening (n=26)		Posterolateral hip strengthening (n=26)	
	Pre (Baseline)	Post (6 weeks)	Pre (Baseline)	Post (6 weeks)
VAS (cm)[!]	5.04 (1.58)	0.57 (1.17)	5.27 (2.01)	1.15 (2.32)
VAS step down (cm)	2.11 (2.30)	0.38 (1.13)	2.46 (2.5)	0.53 (1.02)
AKPS (points)	65.96 (10.03)	79.46 (9.44)	68.5 (8.21)	79.11 (9.73)
Hip Abductor (Nm/kg)	1.05 (0.28)	1.18 (0.22)	1.01 (0.21)	1.26 (0.23)
Hip Adductor (Nm/kg)	0.50 (0.19)	0.70 (0.17)	0.57 (0.17)	0.61 (0.20)
Hip External Rotation (Nm/kg)	0.41 (0.09)	0.47 (0.08)	0.42 (0.12)	0.52 (0.14)
Hip Internal Rotation (Nm/kg)	0.45 (0.14)	0.58 (0.13)	0.53 (0.13)	0.57 (0.12)

Isometric strength muscle assessment using a handheld dynamometer.

Alonazi, et al. 2021

Variables	EMG-BF guided (n=30)		Sham EMG-BF (n=30)	
	Pre (Baseline)	Post (4 weeks)	Pre (Baseline)	Post (4 weeks)

VAS (cm)	6.95 (0.66)	1.68 (0.67)	6.76 (0.69)	4.45 (0.86)
AKPS (points)	42.56 (6.64)	73.26 (7.22)	46.1 (7.74)	69.06(6.04)
Knee extension (kg)	11.47 (1.34)	14.06 (1.03)	11.97 (1.11)	13.18 (1.08)

Isometric strength muscle assessment using an isokinetic dynamometer.

Arslan, et al. 2023

Variables	Online supervised exercise group (n=20)		Home exercise group (n=20)		Control group (n=20)	
	Pre (Baseline)	Post (6 weeks)	Pre (Baseline)	Post (6 weeks)	Pre (Baseline)	Post (6 weeks)
Rest VAS (0–10)	3.85 (2.79)	1.60 (2.11)	4.05 (2.72)	3.35 (3.00)	4.45 (2.35)	4.95 (2.76)
Activity VAS (0–10)[!]	8.20 (1.88)	4.20 (3.27)	7.90 (2.05)	6.15 (2.81)	7.00 (1.69)	7.15 (2.37)
Kujala Questionnaire (0-100)	62.25 (19.02)	77.15 (20.21)	57.45 (10.37)	64.10 (14.24)	59.00 (23.09)	57.85 (23.17)
Knee extension	0.97 (0.33)	1.36 (0.43)	0.96 (0.17)	1.29 (0.31)	1.05 (0.33)	1.07 (0.32)
Knee flexion	0.74 (0.20)	0.92 (0.22)	0.73 (0.18)	0.87 (0.23)	0.70 (0.20)	0.70 (0.20)
Hip posterolateral (clam)	1.38 (0.32)	1.98 (0.41)	1.23 (0.21)	1.66 (0.43)	1.47 (0.38)	1.45 (0.35)

Isometric strength muscle assessment using a handheld dynamometer.

Asif, et al. 2022

Variables	Specific hip strengthening (n=14)		Conventional knee exercises (n=14)	
	Pre (Baseline)	Post (6 weeks)	Pre (Baseline)	Post (6 weeks)
NPRS step up and down	6.14 (1.70)	1.71 (1.06)	6.07 (1.73)	2.28 (1.54)
AKPS (points)	65.43 (21.52)	92.14 (5.12)	65.29 (22.53)	91.75 (7.65)
Knee extension	32.43 (3.81)	33.85 (3.73)	34.43 (4.89)	35.64 (5.06)
Knee flexion	31.71 (5.12)	33.00 (4.55)	32.07 (4.89)	33.14 (4.86)
Hip abduction	13.57 (1.82)	14.78 (1.42)	14.29 (2.16)	15.50 (1.45)
Hip extension	20.07 (2.99)	21.42 (2.73)	19.93 (2.89)	21.50 (3.00)

Isometric strength muscle assessment using a handheld dynamometer.

Aytar, et al. 2011

Variables	Kinesiotape (n=12)		Placebo Kinesiotape (n=10)	
	Pre (Baseline)	Post (45 minutes)	Pre (Baseline)	Post (45 minutes)
Descending Stairs (VAS points)	50.41 (24.35)	46.25 (26.38)	35.00 (21.21)	33.00 (21.62)

Ascending Stairs (VAS points)[!]	52.91 (20.27)	51.25 (23.75)	50.00 (17.63)	48.00 (16.86)
Walking (VAS points)	42.50 (24.54)	42.08 (23.88)	45.00 (23.21)	41.00 (23.78)
Knee extension 60◦/s (Nm)[!]	100.43 (26.20)	106.64 (24.39)	74.97 (24.67)	79.85 (25.55)
Knee extension 180◦/s (Nm)	70.04 (31.21)	77.95 (28.78)	45.69 (15.83)	50.30 (22.41)

Concentric strength muscle assessment using an isokinetic dynamometer.

Azab, et al. 2022

**Variables**	**Traditional Physical Therapy (n=17)**		**Pilates + TPT (n=17)**	
	**Pre (Baseline)**	**Post (3 months)**	**Pre (Baseline)**	**Post (3 months)**
Pain VAS (points)*	7 (5-8)	5 (4-6)	7 (5-9)	4 (2-6)
AKPS (points)	68.33 (11.94)	71.19 (8.65)	67.86 (12.34)	75.49 (11.49)
Hip abduction (Newton)	44.64 (7.83)	45.81 (7.31)	45.98 (8.21)	50.80 (6.62)
Hip external rotation (Newton)	33.24 (3.13)	34.32 (2.49)	36.46 (5.70)	42.43 (6.13)
Knee extension (Newton)	59.10 (7.55)	61.20 (5.83)	59.85 (8.69)	65.52 (6.96)

*Median (min-max).
Isometric strength muscle assessment using a handheld dynamometer.

Baldon, et al. 2014

**Variables**	**Stabilization (n=15)**		**Standard (n=16)**	
	**Pre (Baseline)**	**Post (8 weeks)**	**Pre (Baseline)**	**Post (8 weeks)**
Worst VAS (cm)	6.6 (1.1)	1.4 (1.4)	6.1 (1.8)	3.1 (3.2)
LEFS (points)	55.4 (12.8)	74.3 (4.6)	57.6 (7.2)	70.6 (8.0)
Triple hop (cm)	336.4 (34.8)	375.3 (48.3)	325.1 (82.4)	330.1 (72.5)
Hip abductor torque (Nm/kg)	1.3 (0.2)	1.5 (0.2)	1.2 (0.3)	1.3 (0.3)
Hip adductor torque (Nm/kg)	1.8 (0.2)	1.9 (0.2)	1.8 (0.3)	1.8 (0.3)
Hip lateral rotator torque (Nm/kg)	0.8 (0.1)	0.8 (0.1)	0.7 (0.1)	0.8 (0.1)
Hip medial rotator torque (Nm/kg)	1.4 (0.2)	1.5 (0.2)	1.3 (0.3)	1.3 (0.3)
Knee flexor torque (Nm/kg)	1.3 (0.2)	1.5 (0.1)	1.3 (0.2)	1.3 (0.2)
Knee extensor torque (Nm/kg)	2.9 (0.4)	3.4 (0.4)	2.8 (0.7)	3.1 (0.6)

Eccentric strength muscle assessment using an isokinetic dynamometer (hip muscles at 30º/sec and knee muscles at 60º/sec).

Study	Variables	Exercise and taping group (n=15)		Exercise group (n=15)	
		Pre (Baseline)	Post (12 weeks)	Pre (Baseline)	Post (12 weeks)
Basbug, et al. 2022	VAS stair descending (cm)	5.4 (2.7)	0.1 (0.5)	5.3 (2.6)	2.1 (1.0)
	VAS stair ascending (cm)[!]	5.5 (2.9)	1.2 (0.7)	5.6 (2.4)	2.1 (0.9)
	Knee flexion 60^0/sec (Nm/kg)[!]	0.7 (0.3)	1.0 (0.1)	0.7 (0.3)	0.9 (0.2)
	Knee extension 60^0/sec (Nm/kg)[!]	0.7 (0.3)	0.9 (0.1)	0.8 (0.4)	0.9 (0.3)
	Knee flexion 180^0/sec (Nm/kg)	0.4 (0.2)	0.8 (0.0)	0.5 (0.3)	0.7 (0.1)
	Knee extension 180^0/sec (Nm/kg)	0.4 (0.2)	0.6 (0.1)	0.5 (0.4)	0.6 (0.1)

Concentric strength muscle assessment using an isokinetic dynamometer.

Study	Variables	PT group (n=13)		PT and Electric muscle stimulation group (n=16)	
		Pre (Baseline)	Post (12 weeks)	Pre (Baseline)	Post (12 weeks)
Bily, et al. 2008	VAS (cm)*	5.3 (2.9–7.6)	1.3 (0.4–3.3)	5.6 (3.5–8.3)	1.5 (0.3–2.8)
	AKPS (Points)*	83 (72–88)	90 (85–95)	73 (66–85)	89 (82–96)
	Knee extension at 30º of flexion (N)	94.7 (23)	89.7 (20.8)	108.7 (29)	128 (49)
	Knee extension at 60º of flexion (N)[!]	152 (45)	149 (33)	188 (77)	199 (77)

*Median (quartiles).
Isometric muscle strength assessment using a strain gauge.

Study	Variables (median)	Exercise (n=30)		Guidance (n=30)	
		Pre (Baseline)	Post (6 weeks)	Pre (Baseline)	Post (6 weeks)
Boitrago, et al. 2021	NPRS (cm)	6.7 (1.7)	1.9 (1.8)	6.67 (2.3)	6.07 (2.2)
	AKPS (points)[!]	67.6 (15.2)	85.8 (12.3)	71.6 (11.1)	71.83 (11.8)
	KOS-ADLS (points)	59.2 (14.2)	83 (15.5)	62.27 (19.9)	62 (18.9)
	Hip abduction (Nm/kg)	20.49 (8-39)*	25.06 (16-40)*	20.45 (4.9)	20.55 (19-30)
	Hip external rotation (Nm/kg)	9.17 (5-19)*	10.43 (7-15.3)*	9.7 (2.4)	9.72 (6.2-13.4)
	Knee extension (Nm/kg)	25.47 (9.3-48)*	32.92 (21.6-49.2)*	29.19 (6.8)	28.88 (16-47)

*Median (min-max) – Isometric strength muscle assessment using a handheld dynamometer.

Callaghan, et al. 2001 (not included in meta-analyses)

**Variables**	**Experimental stimulation (n=7)**		**Standard stimulation (n=7)**	
	**Pre (Baseline)**	**Post (6 weeks)**	**Pre (Baseline)**	**Post (6 weeks)**
VAS (cm)	5.3 (1.6)	3.8 (1.8)	4.6 (1.8)	3.4 (2.3)
Number of steps up and down	13.6 (12.6)	18.7 (14.9)	8.1 (2.8)	14.2 (8.5)
AKPS (points)	61.3 (8.4)	67.0 (10.4)	65.4 (11.6)	72.9 (9.1)
Isometric lower limb strength (Nm)	66.2 (19.4)	79.4 (27.7)	70.7 (30.8)	76.5 (28.1)
Isokinetic lower limb strength (Nm)	94.6 (36.2)	106.3 (41.7)	93.6 (26.1)	100.2 (27.0)

Strength muscle assessment using an isokinetic dynamometer.

Callaghan, et al. 2004 (not included in meta-analyses)

**Variables**	**EXPER (n=37)**		**EMPI (n=37)**	
	**Pre (Baseline)**	**Post (6 weeks)**	**Pre (Baseline)**	**Post (6 weeks)**
VAS (cm)*	3 (2-5)	2 (0-4)	3 (1-4)	2 (1-4)
Number of steps up and down*	16 (9-24)	27 (16-46)	13 (7-60)	28 (11-60)
AKPS (points)	72 (12.9)	78 (14.6)	69.3 (14.5)	77.1 (15)
Isometric lower limb strength (Nm)	107.6 (30.8)	118.1 (37.4)	117.8 (43.5)	120.9 (39.9)
Isokinetic lower limb strength (Nm)	104.2 (35.3)	108.1 (36.8)	110.5 (44)	119.1 (47.7)

*Median (interquartile range). Strength muscle assessment using an isokinetic dynamometer.

Celik, et al. 2019

**Variables**	**Superimposed NMES + Standardized Rehabilitation Program (n = 14)**		**Standardized Rehabilitation Program (n = 13)**	
	**Pre (Baseline)**	**Post (6 weeks)**	**Pre (Baseline)**	**Post (6 weeks)**
Kujala score (points)[!]	60.4 (14.8)	73.0 (14.3)	60.9 (15.2)	75.0 (8.1)
Lysholm score (points)	56.1 (16.4)	74.2 (16.6)	55.3 (15.7)	60.1 (14.2)
Knee extension 60^0/sec	130.6 (19.2)	138.0 (19.2)	98.0 (10.0)	123.2 (14.1)
Knee flexion 60^0/sec	74.0 (25.6)	85.4 (20.9)	68.43 (32.9)	76.2 (23.6)

Concentric strength muscle assessment using an isokinetic dynamometer.

Cho, et al. 2025

Variables	Instrument-assisted soft tissue mobilization to the hip and knee (n = 14)			Instrument-assisted soft tissue mobilization to the knee (n = 14)		
	Pre (Baseline)	Immediately post	Follow-up (1 week)	Pre (Baseline)	Immediately post	Follow-up (1 week)
VAS - Running (cm)	5.49 (2.14)	NI	1.30 (1.08)	5.30 (1.45)	NI	1.57 (1.20)
VAS – Step-down (cm)[!]	3.44 (1.29)	0.51 (0.66)	0.56 (1.02)	3.26 (1.41)	1.44 (1.39)	0.47 (0.59)
Step-down (repetitions)	8.79 (4.41)	11.57 (4.13)	12.36 (4.67)	11.00 (2.88)	13.36 (3.67)	13.93 (4.68)
Hip abduction (%BW)	14.11 (2.34)	15.45 (1.87)	15.41 (2.39)	15.79 (3.02)	16.01 (2.94)	15.79 (3.43)
Hip external rotation (%BW)	17.91 (3.24)	18.43 (2.55)	17.44 (5.3)	18.03 (2.02)	18.11 (1.96)	18.47 (2.52)

Isometric strength muscle assessment using a handheld dynamometer.

Clark, et al. 2000

Variables	Exercise + tape (n=20)		Exercise (n=20)		Tape (n=19)		Education (n=22)	
	Pre (Baseline)	Post (3 months)	Pre (Baseline)	Post (3 months)	Pre (Baseline)	Post (3 months)	Pre (Baseline)	Post (3 months)
WOMAC (points)	25.2 (12.5)	11.5 (10.5)	23.7 (12.9)	10.0 (11.8)	33.4 (16.8)	20.9 (15.5)	28.7 (15.4)	13.8 (15.8)
VAS score (points)	75.6 (32.6)	35.9 (28.7)	77.1 (44.4)	30.0 (39.9)	83.9 (39.8)	57.8 (38.7)	76.99 (41.8)	41.8 (40.6)
Knee extension R (KgF)[!]	186.8 (133.0)	280.4 (149.9)	233.2 (148.5)	320.1 (193.6)	198.7 (144.7)	222.5 (143.1)	190.6 (107.8)	258.8 (159.6)
Knee extension L (KgF)	169.4 (100.0)	266.9 (132.9)	224.7 (130.9)	307.9 (162.4)	156.7 (106.1)	189.4 (122.4)	205.6 (130.8)	279.9 (155.1)

Isometric strength muscle assessment using a modified Tornvall chair.

Constantinou, et al. 2022

Variables	Hip and knee strengthening (n=30)		Strengthening with blood flow restriction (n=30)	
	Pre (Baseline)	Post (4 weeks)	Pre (Baseline)	Post (4 weeks)
AKPS (points)	74.1 (71.66, 76.54)	94.1 (92.25, 96.09)	72.7 (69.89, 75.57)	94.9 (93.19, 96.61)
VAS worst (points)[!]	5.73 (5.21, 6.24)	1.30 (0.91, 1.68)	5.40 (4.96, 5.85)	0.76 (0.48, 1.04)
VAS usual (points)	3.80 (3.39, 4.20)	0.40 (0.14, 0.65)	3.64 (3.37, 3.91)	0.22 (0.07, 0.38)
VAS squatting - shallow (points)	3.50 (3.20, 3.80)	0.21 (-0.04, 0.46)	3.35 (3.12, 3.59)	0.15 (0.02, 0.30)
VAS squatting - deep (points)	4.84 (4.41, 5.28)	0.78 (0.34, 1.21)	4.46 (4.09, 4.83)	0.51 (0.22, 0.79)
Knee extension (N)	229.1 (212.6, 245.69)	312.6 (336.69, 424.66)	249.6 (220.66, 278.61)	412.9 (367.20, 458.72)
Hip extension (N)	171.9 (151.7, 195.04)	204.9 (187.04, 222.83)	215.3 (192.22, 235.56)	252.2 (234.33, 270.13)

Hip abduction (N)	114.6 (104.79, 124.52)	133.4 (121.11, 135.71)	124.9 (113.44, 136.49)	147.3 (137.36, 157.35)

* Mean (95% CI). Isometric strength muscle assessment using a handheld dynamometer.

Corum, et al. 2018

Variables	Whole body vibration (n=18)		Control (n=16)	
	**Pre (Baseline)**	**Post (8 weeks)**	**Pre (Baseline)**	**Post (8 weeks)**
VAS (cm)	4.9 (1.5)	1.5 (1.6)	5.0 (1.7)	3.4 (1.9)
AKPS (points)	81.9 (6.2)	87.5 (9.2)	70.4 (15.7)	73.3 (10.8)
Knee extension 60º/s (Nm/kg)[!]	132.3 (36.5)	160.2 (28.7)	153.1 (31.0)	176.8 (25.9)
Knee extension 240º/s (Nm/kg)	60.3 (14.7)	75.2 (14.0)	71.3 (14.5)	79.1 (17.1)
Knee flexion 60º/s (Nm/kg)[!]	85.6 (21.1)	106.6 (15.8)	96.7 (16.6)	121.0 (15.2)
Knee flexion 240º/s (Nm/kg)	49.7 (16.8)	64.2 (11.9)	55.8 (15.0)	65.2 (16.6)

Concentric strength muscle assessment using an isokinetic dynamometer.

Dolak, et al. 2011

Variables	Hip group (n=17)		Quad group (n=16)	
	**Pre (Baseline)**	**Post (4 weeks)**	**Pre (Baseline)**	**Post (4 weeks)**
VAS (cm)	4.6 (2.5)	2.4 (2.0)	4.2 (2.3)	4.1 (2.5)
LEFS (points)	59 (12)	67 (11)	54 (12)	59 (14)
Step-down test (repetitions)	15 (5)	17 (5)	14 (8)	17 (7)
Hip abduction (%)	5.2 (1.5)	6.2 (1.1)	5.7 (2.2)	5.5 (1.9)
Hip external rotation (%)	2.1 (0.7)	2.5 (0.7)	2.1 (1.0)	2.2 (0.8)
Knee extension (%)	6.1 (2.6)	6.8 (1.9)	6.3 (2.1)	6.1 (1.9)

Isometric strength muscle assessment using a handheld dynamometer.

Drew et. 2017

Variables	Hip Training (n=13)		Usual Care (n=11)	
	**Pre (Baseline)**	**Post (6 weeks)**	**Pre (Baseline)**	**Post (6 weeks)**
VAS worst (points)[!]	4.9 (1.7)	4.6 (2.1)	5.3 (2.3)	4.6 (2.2)
VAS average (points)	3.1 (1.4)	2.5 (1.3)	3.7 (2.2)	3.1 (1.9)
AKPS (points)	75.1 (10.1)	80.3 (8.7)	73.6 (12.2)	72.5 (17.0)

Hip abduction 30º/s(Nm)	91.02 (28.45)	99.40 (27.89)	81.82 (31.76)	89.57 (33.43)
Knee extension 60º/s (Nm)	91.44 (28.21)	93.12 (27.19)	94.32 (44.10)	103.95 (46.09)

Concentric strength muscle assessment using an isokinetic dynamometer.

Emamvirdi, et al. 2019

Variables	Valgus Control Instruction (n = 32)		Control group (n=32)	
	Pre (Baseline)	Post (6 weeks)	Pre (Baseline)	Post (6 weeks)
VAS (0 - 10 cm)	6.1 (1.18)	3.1 (1.61)	6 (1.35)	6.1 (1.12)
Single-leg hop test (cm)	112.43 (9.60)	140.12 (11.50)	118.60 (14.01)	117.50 (13.05)
Triple-hop test (cm)	308.31 (58.44)	381.54 (33.40)	343.18 (42.66)	324.12 (35.22)
Crossover-hop test (cm)	301.25 (14.63)	340.06 (18.95)	306.37 (40.47)	299.62 (36.25)
Single-legged 6-m timed hop test (cm)	10.90 (0.60)	10.09 (0.62)	10.16 (0.44)	10.29 (0.67)
Hip abduction 30º/s (Nm/kg)	97.75 (7.22)	98.54 (6.70)	94.16 (4.41)	92.62 (13.72)
Hip adduction 30º/s (Nm/kg)	106.78 (11.06)	95.87 (9.31)	106.86 (8.59)	103.67 (10.17)
Hip external rotation 30º/s (Nm/kg)	40.83 (5.75)	65.22 (6.30)	45.75 (6.30)	43.16 (6.50)
Hip internal rotation 30º/s (Nm/kg)	45.93 (7.20)	53.03 (7.87)	50.91 (10.49)	47.27 (12.17)

Concentric strength muscle assessment using an isokinetic dynamometer.

Esculier, et al. 2017

Variables	Education (n=23)		Exercises (n=23)		Gait retraining (n=23)	
	Pre (Baseline)	Post (8 weeks)	Pre (Baseline)	Post (8 weeks)	Pre (Baseline)	Post (8 weeks)
KOS-ADLS (0–100)	70.7 (8.8)	88.8 (9.0)	70.4 (9.0)	89.3 (10.5)	68.9 (11.1)	83.6 (13.4)
VAS-Usual (0–10)	2.8 (1.5)	1.1 (1.5)	3.4 (1.6)	1.2 (1.3)	3.1 (1.9)	1.6 (1.7)
VAS-Worst (0–10)[!]	5.8 (1.8)	2.4 (1.9)	7.0 (1.4)	3.1 (2.4)	6.0 (2.0)	3.0 (2.7)
VAS-Running (0–10)	5.3 (1.9)	2.4 (2.3)	6.5 (1.6)	2.4 (2.1)	6.1 (2.2)	2.8 (2.7)
Knee extension (%BW)	73.76 (14.79)	70.77 (14.44)	65.39 (16.63)	72.2 (18.51)	73.22 (13.67)	73.17 (11.72)
Hip external rotation (%BW)	23.99 (5.49)	24.13 (4.76)	23.91 (6.86)	24.79 (7.16)	24.65 (5.7)	25.77 (6.54)
Hip abduction (%BW)	60.59 (7.29)	60.93 (8.4)	58.69 (10.47)	62.79 (10.11)	61.96 (9.5)	64.06 (9.98)
Hip extension (%BW)	129.79 (17.67)	128.02 (22.37)	128.33 (23.69)	130.68 (24.96)	133.54 (23.23)	134.09 (25.78)

Isometric strength muscle assessment using a handheld dynamometer.

Ferber, et al. 2014

Variables	Hip and core strengthening (n = 111)		Knee focused (n = 88)	
	**Pre (Baseline)**	**Post (6 weeks)**	**Pre (Baseline)**	**Post (6 weeks)**
VAS (cm)	5.12 (1.66)	1.96 (1.92)	4.96 (1.66)	1.99 (2.05)
AKPS (points)	75.00 (9.74)	87.95 (11.26)	75.62 (9.81)	87.67 (10.53)
Hip abduction (Nm/kg)	3.21 (1.14)	3.58 (1.08)	3.15 (1.19)	3.41 (1.28)
Hip extension (Nm/kg)	2.39 (1.01)	2.66 (1.15)	2.44 (1.09)	2.61 (1.18)
Hip external rotation (Nm/kg)	1.19 (0.42)	1.29 (0.41)	1.18 (0.45)	1.25 (0.44)
Hip internal rotation (Nm/kg)	1.48 (0.55)	1.56 (0.59)	1.42 (0.64)	1.49 (0.62)
Knee extension (Nm/kg)	3.88 (1.59)	4.19 (1.50)	3.93 (1.47)	4.18 (1.60)

Isometric strength muscle assessment using a handheld dynamometer.

Giles, et al. 2017

Variables	Blood flow restriction (n=40)		Standardised quadriceps strengthening (n=39)	
	**Pre (Baseline)**	**Post (8 weeks)**	**Pre (Baseline)**	**Post (8 weeks)**
VAS worst pain in past week (mm)[!]	55.7 (13.9)	27.4 (20.1)	51.4 (15.3)	29.9 (25.6)
AKPS (score)	73.6 (9.9)	86.5 (10.5)	72.6 (10.5)	83.2 (12.3)
VAS stairs, squat or sitting (mm)	58.2 (17.5)	21.6 (25.0)	42.5 (22.8)	23.5 (24.1)
Knee extension (Nm)	131.2 (61.9)	166.4 (59.4)	135.1 (55.1)	158.7 (57.4)

Isometric strength muscle assessment using an isokinetic dynamometer.

Girardi, et al. 2022

Variables	Experimental group – Kaatsu training (n=8)		Control group (n=7)	
	**Pre (Baseline)**	**Post (6 weeks)**	**Pre (Baseline)**	**Post (6 weeks)**
NRPS (0–10)	6.12 (0.64)	1.87 (1.55)	6.71 (1.11)	3.28 (1.11)
AKPS (0-100)	77.75 (11.53)	90.87 (6.08)	79.85 (13.92)	90.28 (6.04)
Knee extension R (kg)[!]	39.47 (5.46)	47.57 (5.50)	37.42 (8.16)	39.28 (7.78)
Knee extension L (kg)	39.72 (7.58)	48.25 (7.16)	38.65 (5.34)	40.20 (6.63)

Isometric strength muscle assessment using a handheld dynamometer.

Glaviano, et al. 2019

Variables	Electrical (n=11)		Sham (n=10)	
	Pre (Baseline)	Post (4 weeks)	Pre (Baseline)	Post (4 weeks)
VAS current pain (cm)	0.8 (0.8)	0.5 (0.6)	1.8 (2.0)	0.7 (0.6)
VAS worst pain (cm)[!]	4.2 (1.1)	1.9 (2.0)	5.6 (1.2)	2.2 (1.2)
AKPS (points)[!]	80.4 (5.0)	87.2 (9.7)	73.1 (7.9)	87.0 (5.6)
ADLS (points)	79.1 (8.5)	88.6 (5.9)	79.6 (12.0)	87.3 (5.2)
LEFS (points)	67.0 (5.9)	72.7 (4.9)	64.2 (10.7)	73.5 (4.6)
Knee extension (N/kg)	4.3 (1.3)	5.5 (3.6)	3.7 (1.7)	4.3 (1.9)
Knee flexion (N/kg)	2.5 (0.6)	2.5 (0.7)	1.7 (0.4)	2.4 (0.6)
Hip abduction (N/kg)	3.0 (0.8)	4.6 (2.9)	2.9 (0.8)	4.3 (2.3)
Hip external rotation (N/kg)	1.7 (0.4)	3.7 (4.4)	1.4 (0.5)	2.8 (2.5)
Hip internal rotation (N/kg)	1.7 (0.5)	1.7 (0.4)	1.2 (0.4)	1.7 (0.4)
Hip extension (N/kg)	3.5 (1.1)	4.2 (1.1)	3.7 (1.5)	4.8 (1.4)

Isometric strength muscle assessment using a handheld dynamometer.

Hammami, et al. 2024

Variables	Passive Compensation of Movement group (n=12)		Isokinetic group (n=12)	
	Pre (Baseline)	Post (6 weeks)	Pre (Baseline)	Post (6 weeks)
KOOS Pain (A.U.)	13.42 (1.28)	2.92 (1.37)	13.58 (1.31)	2.58 (1.38)
Stair Climb Test (seconds)	7.48 (0.73)	4.95 (0.97)	7.41 (1.19)	5.21 (1.04)
10m Walk Test (seconds)	7.63 (0.70)	5.49 (0.50)	7.45 (0.82)	5.72 (0.40)
Chair Stand Test (A.U.)	9.08 (1.00)	19.17 (1.27)	10.08 (1.44)	18.83 (1.11)
Knee flexors 60º/sec (right member - Nm)	0.52 (0.18)	0.52 (0.22)	0.52 (0.12)	0.77 (0.16)
Knee extensors 60º/sec (right member - Nm)	1.06 (0.46)	1.03 (0.39)	1.07 (0.36)	1.41 (0.42)
Knee flexors 60º/sec (left member - Nm)	0.66 (0.16)	0.38 (0.11)	0.42 (0.12)	0.39 (0.13)
Knee extensors 60º/sec (left member - Nm)	1 (0.40)	1 (0.34)	0.79 (0.25)	1.05 (0.21)

A.U.: Arbitrary Unit. Concentric muscle strength assessment using an isokinetic dynamometer (at 60ª/s). The strength of knee flexors and extensors were evaluated.

Hansen, et al. 2023

Variables	Quadriceps-focused exercise (n=100)		Hip-focused exercise (n=100)	
	Pre (Baseline)	Post (12 weeks)	Pre (Baseline)	Post (12 weeks)
NPRS	1.9 (2.2)	1.2 (1.6)	1.8 (1.8)	1.7 (1.9)
AKPS[ǀ]	74.2 (11.6)	82.3 (11.7)	73.3 (13.0)	80.4 (11.4)
KOOS Pain	70.8 (15.6)	80.4 (15.5)	72.2 (14.1)	78.4 (16.1)
KOOS Physical function	84.1 (13.2)	89.9 (11.2)	83.4 (13.1)	88.0 (14.2)
Hip abductors (N)	129.5 (40.9)	142.3 (41.8)	129.5 (41.2)	141.6 (41.8)
Hip adductors (N)	121.2 (40.6)	132.3 (38.6)	122.4 (47.3)	136.3 (48.2)
Hip extensors (N)	175.3 (46.8)	184.9 (48.3)	181.0 (56.0)	193.8 (56.3)
Hip flexors (N)	189.1 (55.8)	205.8 (54)	194.3 (63.0)	204.7 (61.4)
Hip external rotators (N)	101.1 (30.2)	123.5 (30.8)	100.7 (37.7)	120.8 (41.3)
Hip internal rotators (N)	123.9 (100.9)	110.4 (25.1)	109.4 (42.8)	112.1 (35.1)
Knee extensors (quadriceps) (N)	299.2 (113.1)	332.8 (119.2)	292.0 (121.9)	325.5 (125.8)
Knee flexors (hamstrings) (N)	316.6 (117.1)	354.0 (123.8)	302.8 (129.5)	345.1 (133.9)

Isometric muscle strength assessment using a handheld dynamometer.

Hasan, et al. 2022.

Variables	EMG-BF (n=30)		Quad group (n=30)	
	Pre (Baseline)	Post (6 weeks)	Pre (Baseline)	Post (6 weeks)
VAS (cm)	7.0 (0.7)	1.3 (0.8)	6.8 (0.7)	4.5 (0.8)
AKP (points)	42.6 (6.7)	80.4 (5.1)	46.1 (7.5)	69.1 (6.1)
Single-leg triple hop (cm)	501.3 (53.9)	540.7 (51.2)	499.9 (50.6)	509.4 (49.8)
Knee extension (pooled; Nm)[ǀ]	126.97 (29.39)	172.43 (44.86)	117.87 (21.92)	128.97 (23.7)
30 degrees (n=10)	95.1 (10.7)	117.5 (14.2)	93.9 (13.9)	104.8 (13.2)
60 degrees (n=10)	158.6 (12.5)	212.9 (16.2)	133.7 (13.5)	149.6 (15.3)
90 degrees (n=10)	127.2 (16.5)	186.9 (24.8)	126.0 (13.5)	132.5 (16.3)

Isometric muscle strength assessment using an isokinetic dynamometer.

Hasan, et al. 2025

Variables	Patellar taping + Quadriceps strengthening (n=26)		Placebo taping + Quadriceps strengthening (n=27)	
	**Pre (Baseline)**	**Post (6 weeks)**	**Pre (Baseline)**	**Post (6 weeks)**
NPRS (points)	6.98 (0.61)	1.38 (0.65)	7.06 (0.73)	4.26 (0.81)
AKPS (points)	42.27 (6.26)	80.38 (4.78)	43.96 (7.01)	69.52 (5.12)
Single-leg triple hop test (cm)	245.08 (24.13)	356.88 (36.42)	248.41 (18.89)	268.19 (14.33)
Knee extension	109.85 (6.25)	154.38 (9.10)	108.33 (6.23)	122.37 (9.51)

Isometric muscle strength assessment using an isokinetic dynamometer.

Herrington, et al. 2007

Variables	Non–Weight-Bearing (n=15)		Weight-Bearing (n=15)		Control group (n=15)	
	**Pre (Baseline)**	**Post (6 weeks)**	**Pre (Baseline)**	**Post (6 weeks)**	**Pre (Baseline)**	**Post (6 weeks)**
VAS with step up and down (points)	4.99 (1.51)	2.79 (1.91)	5.19 (1.21)	1.99 (1.00)	5.19 (1.00)	5.99 (1.9)
Kujala (points)	70.86 (10.11)	88.93 (12.13)	55.92 (10.36)	90.91 (6.1)	58.12 (11.83)	46.11 (8.05)
Knee extension strength	115.30 (44.7)	144.91 (42.99)	85.05 (40.31)	120.00 (44.13)	110.18 (39.78)	70.96 (38.08)

Isometric muscle strength assessment using an isokinetic dynamometer.

Hong, et al. 2023

Variables	Exercise program plus health education (n=19)		Health education (n=19)	
	**Pre (Baseline)**	**Post (6 weeks)**	**Pre (Baseline)**	**Post (6 weeks)**
VAS worst pain (0-100 mm)[!]	50.5 (12.0)	29.4 (12.8)	49.6 (9.2)	47.8 (8.8)
VAS ADL pain (0-100 mm)	49.0 (10.0)	23.7 (8.8)	46.0 (8.8)	43.6 (11.0)
AKPS (points)	74.2 (9.8)	85.1 (4.9)	74.2 (9.3)	76.0 (8.2)
Knee extensor 60º/sec (Nm/kg x 100)	171.5 (52.2)	190.9 (45.3)	168.0 (38.6)	167.3 (37.6)
Knee flexor 60º/sec (Nm/kg x 100)	108.4 (36.8)	108.9 (35.9)	109.9 (24.4)	109.3 (23.9)

Concentric muscle strength assessment using an isokinetic dynamometer (at 60º/s).

Hott, et al. 2019

Variables	Patient education with isolated hip-focused exercise (n=39)		Traditional knee-focused exercise (n=37)		Free physical activity (n=36)	
	**Pre (Baseline)**	**Post (6 weeks)**	**Pre (Baseline)**	**Post (6 weeks)**	**Pre (Baseline)**	**Post (6 weeks)**
AKPS (points)	64.5 (61.1-67.9)	73.3 (69.9-76.7)	67.2 (62.7-71.7)	74.5 (69.7-79.4)	65.3 (61.2-69.5)	73.6 (69.1-78.1)
VAS usual pain (points)	4.4 (3.8-4.9)	3.1 (2.5-3.7)	4.3 (3.6-5.0)	2.6 (1.9-3.2)	3.7 (3.0-4.4)	2.8 (2.0-3.5)
VAS worst pain (points)[!]	6.5 (5.8-7.1)	5.3 (4.7-6.0)	6.0 (5.2-6.8)	4.1 (3.2-5.0)	5.8 (5.1-6.5)	4.7 (3.9-5.5)
Step-down (repetitions)	16.7 (15.2-18.2)	18.7 (17.1-20.2)	15.6 (13.6-17.6)	18.1 (15.8-20.4)	16.4 (14.4-18.4)	18.6 (16.2-20.9)c
Hip abduction (N)	138 (124-152)	150 (135-166)	126 (110-141)	143 (128-158)	138 (122-154)	141 (124-158)
Hip external rotation (N)	111 (96-125)	124 (109-139)	103 (88-118)	117 (102-132)	114 (97-131)	119 (103-136)
Knee extension (N)	321 (283-358)	345 (311-379)	317 (272-362)	326 (283-368)	337 (289-386)	322 (273-371)

* Mean (95% CI) – Isometric muscle strength assessment using a force sensor.

Ismail, et al. 2013

Variables	Closed kinetic chain + Hip (n=16)		Closed kinetic chain (n=16)	
	**Pre (Baseline)**	**Post (6 weeks)**	**Pre (Baseline)**	**Post (6 weeks)**
VAS (cm)	5.3 (1.6)	2.0 (1.1)	4.5 (1.8)	2.3 (1.1)
AKPS (points)	71.5 (7.8)	85.1 (6.2)	76.4 (10.4)	85.0 (6.7)
Concentric hip abduction 60º/sec (Nm/BMI)	1.7 (0.6)	2.4 (0.8)	2.1 (0.6)	2.5 (0.7)
Eccentric hip abduction 60º/sec (Nm/BMI)[!]	2.0 (0.6)	2.4 (0.8)	2.2 (0.5)	2.4 (0.5)
Concentric hip external rotation 60º/sec (Nm/BMI)	0.9 (0.6)	1.3 (0.6)	1.0 (0.4)	1.2 (0.4)
Eccentric hip external rotation 60º/sec (Nm/BMI)[!]	1.4 (0.4)	1.8 (0.8)	1.4 (0.4)	1.6 (0.3)

Muscle strength assessment using an isokinetic dynamometer.

Kamel, et al. 2024

Variables	Short foot exercises plus hip and knee exercises (n=14)		Hip and knee exercises (n=14)	
	**Pre (Baseline)**	**Post (6 weeks)**	**Pre (Baseline)**	**Post (6 weeks)**
VAS (0-10 cm)	5.8 (1.1)	0.7 (0.6)	6.2 (1)	3.3 (1.2)
AKPS (points)	79.6 (4.9)	96.6 (3.9)	81 (5.3)	92.6 (3.7)
Hip abductors	132 (39)	198 (43)	116 (25)	179 (21)
Knee extensors	102.2 (15.4)	139.4 (20)	104.2 (22.8)	125.3 (19.8)

Isometric muscle strength assessment using a handheld dynamometer.

Keet, et al. 2007	**Variables**	**PFP group (n = 15, crossover)**		
		**No Tape[!]**	**Placebo**	**Tape[!]**
	VAS pain during contraction (0-10)	1.7984 (0.8759; 2.7209)	2.2015 (1.0387; 3.3643)	1.7984 (0.8372; 2.7596)
	Knee extension (N)	362 (311; 414)	348 (307; 390)	376 (316; 436)

Isometric muscle strength assessment using an isokinetic dynamometer.

Khayambashi, et al. 2012	**Variables**	**Exercise group (n=14)**		**Control group (n=14)**	
		**Pre (Baseline)**	**Post (8 weeks)**	**Pre (Baseline)**	**Post (8 weeks)**
	VAS (0 to 10 cm)	7.9 (1.7)	1.4 (1.9)	6.6 (2.0)	6.7 (2.4)
	WOMAC (0 to 96 numbers)	54.0 (18.1)	10.7 (16.1)	55.9 (15.7)	59.9 (12.6)
	Hip abduction R (%)[!]	11.6 (2.3)	15.3 (2.5)	12.3 (2.9)	11.2 (2.5)
	Hip abduction L (%)	11.2 (2.7)	15.9 (3.1)	12.5 (3.7)	11.4 (3.1)
	Hip external rotation R (%)[!]	8.6 (2.3)	11.8 (2.2)	8.9 (2.1)	8.3 (2.3)
	Hip external rotation L (%)	7.0 (1.8)	10.9 (2.6)	7.5 (1.6)	7.3 (1.9)

Isometric muscle strength assessment using a handheld dynamometer.

Khojasteh, et al. 2024 (not included in meta-analyses)	**Variables**	**Physiotherapy plus EMG biofeedback (n=15)**		**Physiotherapy (n=15)**	
		**Pre (Baseline)**	**Post (8 weeks)**	**Pre (Baseline)**	**Post (8 weeks)**
	VAS (0-10 cm)	Not informed			
	Hip abductors (N)	23.44 (3.12)	29.65 (4.33)	24.1 (3.9)	24.6 (2.7)
	Hip external rotators (N)	21.53 (2.65)	23.41 (1.11)	20.8 (0.9)	23.8 (2.1)

Isometric muscle strength assessment using a load cell.

Study	Variables	Control group (n=15)		Short foot exercise group (n = 15)	
		Pre (Baseline)	Post (6 weeks)	Pre (Baseline)	Post (6 weeks)
	VAS Walking pain (points)	4.13 (3.22)	3.46 (2.99)	3.66 (2.28)	1.40 (1.54)
	VAS Sitting pain (points)	3.13 (3.13)	3.00 (3.22)	4.93 (2.46)	2.33 (1.87)
	VAS Stair-up pain (points)	4.46 (2.87)	3.30 (2.38)	5.80 (2.11)	2.20 (1.52)
	VAS Stair-down pain (points)	4.86 (3.02)	3.63 (3.00)	5.06 (1.75)	2.26 (1.22)
	VAS Squatting pain (points)[!]	5.20 (3.23)	4.00 (3.25)	6.73 (2.28)	3.53 (1.76)
	VAS Nocturnal pain (points)	1.80 (2.65)	1.80 (2.65)	3.60 (3.35)	1.33 (1.83)
	Kujala Patellofemoral Score (points)	63.86 (10.07)	72.60 (12.14)	62.06 (14.16)	72.73 (11.39)
	Hip abduction R (N)[!]	35.40 (5.19)	38.44 (4.31)	34.71 (6.27)	39.52 (4.70)
Kısacık, et al. 2021	Hip abduction L (N)	31.96 (4.70)	35.99 (4.51)	32.65 (5.58)	36.77 (3.92)
	Hip extension R (N)[!]	33.63 (3.92)	36.57 (4.41)	34.22 (3.53)	40.50 (4.31)
	Hip extension L (N)	32.55 (4.80)	37.26 (4.90)	34.12 (6.47)	40.10 (4.60)
	Knee flexion R (N)[!]	33.34 (5.78)	37.95 (7.25)	35.69 (4.02)	40.59 (5.88)
	Knee flexion L (N)	32.65 (5.88)	39.03 (5.00)	35.20 (5.00)	39.22 (6.37)
	Knee extension R (N)[!]	25.00 (2.54)	27.36 (2.35)	25.49 (3.23)	28.83 (2.94)
	Knee extension L (N)	24.81 (4.60)	28.04 (4.11)	24.90 (3.72)	35.79 (29.81)
	Ankle dorsiflexion R (N)	22.65 (4.02)	24.71 (3.43)	23.33 (2.64)	25.69 (2.94)
	Ankle dorsiflexion L (N)	22.75 (2.45)	25.39 (2.05)	24.12 (2.54)	25.98 (2.15)
	Ankle plantar flexion R (N)	30.59 (5.78)	33.53 (5.78)	30.10 (5.29)	31.67 (4.90)
	Ankle plantar flexion L (N)	26.08 (5.88)	29.22 (6.57)	25.49 (4.02)	28.24 (3.62)
	Flexor hallucis longus R (N)	21.57 (2.25)	22.26 (2.74)	21.77 (2.05)	23.04 (2.25)
	Flexor hallucis longus L (N)	25.10 (14.41)	22.35 (2.94)	20.69 (2.25)	22.84 (2.05)

Isometric muscle strength assessment using a handheld dynamometer.

Kong, et al. 2025

Variables	Intermittent blood flow restriction (n=21)		Control group (n=21)	
	Pre (Baseline)	Post (8 weeks)	Pre (Baseline)	Post (8 weeks)
VAS (mm)	62.33 (17.73)	19.25 (13.66)	48.00 (15.0)	16.57 (13.56)
AKPS (points)	82.62 (7.90)	90.18 (6.24)	83.52 (8.29)	89.76 (7.77)
Knee extension 60º/s (Nm)	140.59 (49.32)	158.53 (44.94)	128.86 (43.97)	150.18 (45.68)

Concentric muscle strength assessment using an isokinetic dynamometer.

Kowall, et al. 1996 (not included in meta-analyses)

Variables	Tape group (n = 12)		Control group (n = 13)	
	Pre (Baseline)	Post (4 weeks)	Pre (Baseline)	Post (4 weeks)
VAS (0-10)	Not informed			
Knee extension 60º/sec (Nm)				
Knee extension 180º/sec (Nm)				

Concentric muscle strength assessment using an isokinetic dynamometer.

Kumar, et al. 2024

Variables	Positional release technique of gluteus medius trigger point (n=32)		Myofascial release of gluteus medius trigger point (n=32)	
	Pre (Baseline)	Post (6 weeks)	Pre (Baseline)	Post (6 weeks)
VAS (cm)[!]	5.46 (0.57)	0.20 (0.48)	5.33 (0.66)	0.06 (0.25)
Algometry	2.77 (0.55)	6.26 (1.44)	2.91 (0.48)	5.97 (0.55)
AKPS (points)	53.13 (11.59)	94.33 (4.59)	52.63 (9.08)	94.40 (4.53)
Hip abductor	7.26 (1.38)	12.21 (2.03)	7.30 (1.57)	12.03 (1.93)

Isometric muscle strength assessment using a handheld dynamometer.

Kurt, et al. 2016

Variables	KT group (n=55)		PKT group (n=51)	
	Pre (Baseline)	Post (2 days)	Pre (Baseline)	Post (2 days)
VAS (points)	4.7 (2.3)	2.4 (1.6)	4.2 (1.8)	4.1 (1.7)
AKPS (points)	67.9 (9.6)	70.5 (9.8)	73.8 (7.8)	73.6 (7.8)

Knee flexion 60°/sec (Nm)[!]	44.4 (23.9)	46.2 (24.5)	49.9 (22.7)	51.3 (22.0)
Knee flexion 180°/sec (Nm)	37.7 (18.9)	38.7 (19.1)	40.1 (18.7)	41.4 (17.7)
Knee extension 60°/sec (Nm)[!]	72.8 (23.9)	76.2 (28.1)	82.9 (21.7)	84.9 (18.4)
Knee extension 180°/sec (Nm)	61.8 (20.1)	64.2 (18.9)	70.3 (18.3)	71.4 (17.1)

Concentric muscle strength assessment using an isokinetic dynamometer.

Lee, et al. 2020

Variables	Static quadriceps stretching (n=25)		Dynamic quadriceps stretching (n=21)	
	**Pre (Baseline)**	**Post (12 weeks)**	**Pre (Baseline)**	**Post (12 weeks)**
VAS (cm)	4 (1)	3 (0.9)	5 (1.2)	1 (0.7)
AKPS	58 (9.1)	68 (7.7)	57 (8.2)	76 (6.5)
Knee flexion 180º/sec (%)	69 (24.6)	100 (17)	82 (40)	103 (11.5)
Knee extension 180º/sec (%)	105 (38.7)	170 (52.6)	102 (41.8)	182 (54.2)

Concentric muscle strength assessment using an isokinetic dynamometer.

Lee, et al. 2021

Variables	Static stretching group (n=20)		Dynamic stretching group (n=24)	
	**Pre (Baseline)**	**Post (6 months)**	**Pre (Baseline)**	**Post (6 months)**
VAS (points)	5 (0.9)	3 (1.0)	5 (1.1)	2 (1.0)
AKPS (points)	48 (6.1)	70 (6.8)	51 (5.8)	74 (8.4)
Knee extension 180º/sec (%)	94 (40)	165 (49.1)	93 (37.6)	183 (46.5)

Concentric muscle strength assessment using an isokinetic dynamometer.

Lee, et al. 2025

Variables	Digital therapeutic app (n=18)		Control group (n=17)	
	**Pre (Baseline)**	**Post (8 weeks)**	**Pre (Baseline)**	**Post (8 weeks)**
NPRS – Usual pain (points)	3.9 (2.0)	2.3 (1.7)	3.7 (1.5)	3.1 (2.1)
NPRS – Worst pain (points)[!]	4.8 (2.5)	3.2 (2.2)	5.2 (1.9)	3.6 (2.7)
Pain during stair ascent (0-10)	3.7 (2.4)	2.2 (2.0)	3.4 (2.1)	2.2 (1.8)
Pain during stair descent (0-10)	4.0 (2.2)	2.1 (1.6)	4.0 (2.1)	2.8 (2.4)
Pain during sit-to-stand transition (0-10)	2.8 (2.6)	1.3 (1.4)	3.7 (2.3)	2.4 (1.9)

Pain during running (0-10)	4.5 (2.7)	2.5 (1.7)	4.7 (2.0)	3.1 (2.1)
AKPS (points)	74.5 (11.8)	81.45 (12.9)	73.5 (12.8)	78.3 (17.3)
Knee extension (Nm)	78.0 (19.4)	100.8 (20.9)	68.5 (32.2)	82.9 (33.5)

Isometric muscle strength assessment using an isokinetic dynamometer.

Lee, et al. 2026 (not included in meta-analyses)

Variables	Exercise + Blood flow restriction (n=20)		Exercise only (n=19)	
	Pre (Baseline)	Post (6 weeks)	Pre (Baseline)	Post (6 weeks)
PPT – Rectus femoris (N/cm^2)	31.48 (15.16)	44.68 (14.16)	31.81 (13.07)	37.13 (12.87)
PPT – Vastus medialis (N/cm^2)	33.84 (17.01)	45.04 (16.70)	31.23 (12.88)	36.01 (12.02)
PPT – Vastus lateralis (N/cm^2)	30.93 (12.43)	40.89 (13.66)	25.80 (8.26)	31.87 (8.96)
PPT – Semitendinosus (N/cm^2)	33.03 (12.09)	47.89 (11.51)	38.15 (14.07)	45.40 (13.19)
PPT – Biceps femoris (N/cm^2)	36.45 (14.50)	48.39 (12.47)	37.00 (9.55)	42.20 (9.01)
Stair-descending task	4.30 (0.80)	1.75 (0.85)	3.53 (0.61)	1.89 (0.80)
Knee extension (kgf)	18.12 (4.62)	25.54 (4.11)	19.44 (3.50)	21.69 (3.81)
Knee flexion (kgf)	11.20 (3.07)	16.43 (4.08)	13.02 (2.67)	14.99 (2.60)

Isometric muscle strength assessment using a traction dynamometer.

León-Morillas et al. 2024

Variables	Standard Physiotherapy Group (n=25)		Flossing Band and Physiotherapy Group (n=25)	
	Pre (Baseline)	Post (8 weeks)	Pre (Baseline)	Post (8 weeks)
VAS (cm)	5.8 (4.9, 6.7)	2.8 (1.4, 5.1)	5.2 (3.7, 5.9)	1.8 (1.1, 3.9)
AKPS (points)[!]	69.5 (67.1, 71.9)	81.5 (77.1, 91.9)	73.8 (67.2, 78.4)	86.8 (75.2, 90.4)
LEFS (points)	37.7 (28.2, 45.2)	62.7 (60.2, 65.2)	39.1 (27.6, 45.8)	69.2 (67.6, 70.8)
Knee extensors (kg)	16.3 (15.5, 25.1)	21.3 (18.5, 35.1)	14.1 (12.2, 32.6)	27.9 (21.2, 46.6)

Isometric muscle strength assessment using a handheld dynamometer.

Liu, et al. 2023

Variables	Instrument-assisted Soft Tissue Mobilization (n=13)		Tui-na manipulation (n=12)	
	**Pre (Baseline)**	**Post (4 weeks)**	**Pre (Baseline)**	**Post (4 weeks)**
VAS (cm)	14.92 (2.86)	2.25 (1.38)	20.58 (2.86)	3.58 (1.38)
LKSS (points)	62.46 (4.17)	93.36 (2.93)	61.33 (3.99)	85.83 (2.80)
Leg extension (leg-press)	76.15 (39.25)	92.52 (42.01)	90.97 (61.55)	109.07 (57.97)

Isometric muscle strength assessment using a testing system.

Liu, et al. 2023 (not included in meta-analyses)

Variables	Instrument-assisted Soft Tissue Mobilization with blood flow restriction (n=13)		Instrument-assisted Soft Tissue Mobilization (n=13)	
	**Pre (Baseline)**	**Post (4 weeks)**	**Pre (Baseline)**	**Post (4 weeks)**
VAS (cm)	23.23 (13.91)	2.77 (3.22)	14.54 (5.98)	2.92 (5.75)
LKSS (points)	61.62 (12.64)	84.08 (9.71)	70.85 (17.20)	94.54 (7.23)
Leg extension (leg-press)	100.77 (53.63)	118.06 (52.04)	61.50 (37.90)	79.62 (38.00)

Isometric muscle strength assessment using a testing system.

Mahmoud, et al. 2015

Variables	Balance group (n=30)		Control group (n=30)	
	**Pre (Baseline)**	**Post (6 weeks)**	**Pre (Baseline)**	**Post (6 weeks)**
VAS (cm)	5.3 (1.7)	1.9 (0.9)	5.6 (2)	3.5 (1.5)
Hip abduction (Nm/kg)	87.8 (2.9)	120.1 (10.6)	89.03 (3.5)	102.8 (8.2)

Isometric muscle strength assessment using an isokinetic dynamometer.

Mason, et al. 2011

Variables	Taping group (n=15)		Strengthening group (n=15)		Stretching group (n=15)		Control group (n=15)	
	**Pre (Baseline)**	**Week 1**	**Pre (Baseline)**	**Week 1**	**Pre (Baseline)**	**Week 1**	**Pre (Baseline)**	**Week 1**
VAS up stairs (Points)	1.55 (1.7)	1.0 (1.1)	1.73 (1.7)	0.53 (0.8)	2.15 (2.2)	1.39 (2)	0.81 (0.8)	0.7 (0.9)
VAS down stairs (Points)	1.56 (1.2)	1.09 (1)	2.32 (2.4)	0.99 (1.8)	3.43 (3.5)	2.1 (2.2)	1.1 (1.6)	0.82 (1.2)
VAS step down (Points)	1.1 (1.8)	0.85 (1.3)	1.41 (1.7)	0.72 (1.6)	2.82 (3.2)	1.85 (2.8)	0.69 (1.2)	0.22 (0.47)
VAS self-selected activity (Points)[!]	3.43 (2.4)	1.83 (1.6)	3.25 (2.1)	1.74 (1.7)	4.97 (2.4)	2.65 (2)	3.36 (1.9)	2.26 (1.5)
Knee extension 60º/sec (ft/lbs)	72.2 (32)	71.67 (32)	65.4 (28)	76 (27)	57.87 (29)	63.93 (24)	66.13 (28)	67.87 (27)

Concentric muscle strength assessment using an isokinetic dynamometer.

Melo, et al. 2018

Variables	KT with tension in the VMO (TG; n=18)		KT without tension in the VMO (WTG; n=18)		Nothing (CG; n=18)	
	**Pre (Baseline)**	**Post (3 days)**	**Pre (Baseline)**	**Post (3 days)**	**Pre (Baseline)**	**Post (3 days)**
AKPS (points)	74.66 (8.4)	NI	70.38 (7.8)	NI	73.27 (10.02)	NI
NPS (points)	2.44 (1.54)	2.66 (2.11)	3.83 (2.33)	2.83 (1.85)	3.61 (2.59)	3.23 (1.75)
Knee extension 60º/sec (%)[1]	177.95 (54.94)	185.14 (49.93)	194.71 (44.14)	188.08 (33.86)	179.91 (60.91)	173.21 (66.12)
Knee extension 60º/sec (Nm)	107.09 (37.46)	111.53 (35.23)	114.27 (32.80)	110.86 (28.18)	106.20 (35.47)	101.85 (38.60)

Concentric muscle strength assessment using an isokinetic dynamometer. The strength of quadriceps was evaluated.

Melo, et al. 2024

Variables	Exercise combined with NMES (n=17)		Exercise only (n=17)	
	**Pre (Baseline)**	**Post (8 weeks)**	**Pre (Baseline)**	**Post (8 weeks)**
NPS (points)	5.8 (1.4)	2.2 (1.6)	5.1 (1.4)	2.2 (2.3)
AKPS (points)	68 (6.4)	78 (6.6)	65 (7.4)	80 (9.5)
Knee extension PT/BW (%)	185 (53.4)	187 (57.7)	157 (33.3)	156 (41.3)
Knee extension Mean PT (Nm)	99 (35.4)	102 (37.5)	91 (20.4)	89 (21.9)
Hip abduction PT/BW (%)	114 (12.3)	114 (25.8)	104 (12.9)	106 (30.7)
Hip abduction Mean PT (Nm)	56 (12.3)	53 (11)	51 (12.9)	54 (13.6)

Concentric muscle strength assessment using an isokinetic dynamometer.

Nakagawa, et al. 2008

Variables	Quadriceps strengthening + hip abductor and lateral rotator strengthening (n=7)		Quadriceps strengthening (n=7)	
	**Pre (Baseline)**	**Post (6 weeks)**	**Pre (Baseline)**	**Post (6 weeks)**
Usual pain VAS (cm)	3.8 (2.1)	1.1 (1.2)	4.7 (2.6)	4.0 (2.6)
Worst pain VAS (cm)[!]	5.0 (2.1)	1.4 (1.3)	5.5 (1.5)	3.4 (1.9)
Stair-climbing pain VAS (cm)	3.5 (3.7)	0.4 (0.6)	5.0 (3.4)	2.6 (2.8)
Descending stair pain VAS (cm)	4.5 (3.1)	0.3 (0.4)	4.7 (3.3)	2.0 (2.4)
Squatting pain VAS (cm)	5.7 (3.2)	0.4 (0.6)	4.8 (3.0)	3.0 (3.1)
Prolonged sitting pain VAS (cm)	2.9 (3.2)	1.1 (1.6)	5.2 (2.8)	2.9 (3.1)
Knee extension 30º/sec (Nm/Kg)	264.9 (84.8)	318.9 (96.8)	283.6 (45.0)	301.9 (63.4)
Hip abduction 30º/sec (Nm/Kg)	89.1 (29.5)	102.2 (19.8)	114.6 (32.1)	120.4 (30.4)
Hip external rotation 30º/sec (Nm/Kg)	55.5 (14.6)	59.4 (18.9)	60.4 (16.5)	62.9 (24.9)

Eccentric muscle strength assessment using an isokinetic dynamometer.

Ozlu, et al. 2024

Variables	High-intensity laser (n=15)		Ultrasound and TENS (n=15)		Ultrasound and interferential (n=15)	
	**Pre (Baseline)**	**Post (2 weeks)**	**Pre (Baseline)**	**Post (2 weeks)**	**Pre (Baseline)**	**Post (2 weeks)**
VAS (cm)	6.80 (1.74)	0.93 (1.38)	6.86 (2.09)	4.73 (2.96)	6.46 (1.76)	3.20 (2.21)
AKPS (points)[!]	64.40 (14.53)	89.86 (7.55)	66.40 (10.08)	74.46 (10.39)	64.40 (13.17)	76.06 (10.15)
LEFS (points)	50.73 (14.56)	74.40 (8.50)	49.26 (14.37)	57.13 (14.20)	49.26 (14.65)	59.00 (12.22)
Hamstring strength	3.49 (0.80)	4.67 (1.01)	3.99 (0.73)	4.26 (0.58)	3.24 (0.90)	3.47 (0.68)
Quadriceps strength	3.30 (0.68)	4.11 (0.72)	3.92 (0.91)	4.02 (0.85)	3.30 (0.55)	3.57 (0.51)

Isometric muscle strength assessment using a handheld dynamometer.

Pompeo, et al. 2022

Variables	Standard (n=25)		Distal (n=25)	
	Pre (Baseline)	Post (12 weeks)	Pre (Baseline)	Post (12 weeks)
VAS (points)	5.40 (1.55)	2.56 (2.11)	4.80 (1.35)	2.72 (1.49)
AKPS (points)	68.12 (9.95)	77.72 (13.75)	72.32 (11.85)	78.68 (13.44)
Hip abduction (kgf/kg)	0.20 (0.05)	0.22 (0.06)	0.20 (0.06)	0.19 (0.05)
Hip extension (kgf/kg)	0.34 (0.09)	0.38 (0.09)	0.35 (0.09)	0.33 (0.08)
Hip external rotation (kgf/kg)	0.20 (0.04)	0.20 (0.06)	0.19 (0.04)	0.19 (0.04)
Knee extension (kgf/kg)	0.57 (0.15)	0.62 (0.20)	0.55 (0.10)	0.58 (0.11)
Knee flexion (kgf/kg)	0.38 (0.09)	0.38 (0.09)	0.36 (0.10)	0.36 (0.09)
Foot eversion (kgf/kg)	0.23 (0.08)	0.25 (0.06)	0.24 (0.08)	0.26 (0.06)
Foot inversion (kgf/kg)	0.31 (0.08)	0.32 (0.10)	0.32 (0.08)	0.33 (0.08)

Isometric muscle strength assessment using a handheld dynamometer.

Qureshi, et al. 2025 (not included in meta-analyses)

Variables	Exercise + Dry needling (n=15)		Exercise only (n=15)	
	Pre (Baseline)	Post (4 weeks)	Pre (Baseline)	Post (4 weeks)
AKPS (points)	51.6 (7.9)	94.8 (2.2)	50.1 (8.1)	74.13 (8.4)
LEFS (points)	27.2 (6.3)	75.1 (2.9)	22.53 (6.2)	64.8 (6.6)
NPRS (points)*	6.0 (1)	2.0 (1)	7.0 (1.3)	1.0 (2)
Manual muscle testing – Right quadriceps (RM)*	3 (0)	4 (1)	3 (0)	5 (1)
Manual muscle testing – Left quadriceps (RM)*	3 (0)	4 (0)	3 (0)	4 (1)

*Repetition maximum median (IQR); Muscle strength assessment using a manual testing.

Rabelo, et al. 2017

Variables	Motor control (n=17)		Exercise only (n=17)	
	**Pre (Baseline)**	**Post (4 weeks)**	**Pre (Baseline)**	**Post (4 weeks)**
NPRS (points)	6.1 (1.4)	2.0 (1.7)	6.6 (1.0)	2.2 (1.6)
AKPS (points)	67.1 (7.6)	85.8 (9.2)	67.5 (11.3)	83.7 (8.3)
Hip abduction (%BW)	23.9 (9.6)	29.5 (8.2)	23.2 (8.7)	29.1 (8.2)
Hip external rotation (%BW)	11.2 (3.1)	13.5 (2.9)	11.7 (3.5)	14.3 (3.4)
Knee extension (%BW)	39.4 (14.1)	47.0 (11.1)	38.1 (11.2)	47.5 (7.3)

Isometric muscle strength assessment using a handheld dynamometer.

Riel, et al. 2017

Variables	Feedback group (n=20)		Control group (n=20)	
	**Pre (Baseline)**	**Post (6 weeks)**	**Pre (Baseline)**	**Post (6 weeks)**
KPS (score)	69.2 (11.6)	76.5 (12.4)	67.1 (11.2)	76.1 (9.3)
Knee extension (N/kg)	3.7 (1.4)	4.0 (1.3)	3.8 (1.0)	4.0 (0.7)
Hip extension (N/kg)	2.6 (1.2)	3.2 (1.6)	2.7 (1.2)	3.2 (1.6)
Hip abduction (N/kg)	2.0 (0.5)	2.1 (0.5)	1.9 (0.6)	2.1 (0.5)

Isometric muscle strength assessment using a handheld dynamometer.

Rodrigues, et al. 2021

Variables	Anodic stimulus plus resistance training (n=14)		Placebo stimulus plus resistance training (n=14)	
	**Pre (Baseline)**	**Post (4 weeks)**	**Pre (Baseline)**	**Post (4 weeks)**
VAS (cm)*	5.0 (1 - 5)	3.5 (0 - 7)	3.5 (0 - 5)	3.0 (0 - 8)
Knee extension (kg)	35.6 (11.9)	45.0 (11.9)	28.6 (8.4)	35.1 (9.2)

*Median (min – max)
Concentric muscle strength assessment using a 10-repetition maximum test.

Roh, et al. 2021

Variables	Electro-cryotherapy plus exercise (n=15)		Exercise alone (n=15)	
	**Pre (Baseline)**	**Post (3.5 weeks)**	**Pre (Baseline)**	**Post (3.5 weeks)**
VAS during activities of daily living (cm)	4.3 (0.8)	1.3 (0.4)	4.0 (0.9)	1.2 (0.3)
VAS at least (cm)	1.2 (0.6)	0.6 (0.4)	0.9 (0.7)	0.5 (0.3)
VAS at worst (cm)[!]	7.3 (0.7)	4.1 (1.1)	6.6 (0.9)	3.9 (0.8)
LEFS (scores)	49.3 (5.5)	59.1 (5.8)	56.3 (5.1)	60.6 (6.8)
Knee extension (Nm/kg)	2.6 (0.3)	2.9 (0.4)	2.6 (0.3)	3.0 (0.4)

Isometric muscle strength assessment using an isokinetic dynamometer.

Saad, et al. 2018

Variables	Quadriceps strengthening group (n = 10)		Hip strengthening group (n = 10)		Stretching group (n = 10)		Control group (n=10)	
	**Pre (Baseline)**	**Post (8 weeks)**	**Pre (Baseline)**	**Post (8 weeks)**	**Pre (Baseline)**	**Post (8 weeks)**	**Pre (Baseline)**	**Post (8 weeks)**
VAS (cm)	6.34 (1.85)	0.56 (0.89)	5.05 (1.27)	0.55 (0.8)	4.08 (0.82)	0.14 (0.25)	4.03 (1.03)	3.69 (0.6)
AKPS (points)	76.9 (8.72)	90.11 (6.11)	77.4 (5.46)	91.8 (5.67)	79.8 (9.21)	91.0* (6.62)	80.8 (10.67)	81.9 (8.41)
Hip abduction (Kgf)	10.96 (2.99)	11.34 (3.74)	11.87 (2.68)	15.14 (2.48)	13.02 (6.02)	13.29 (5.45)	12.06 (3.58)	11.4 (3.22)
Hip addution (Kgf)	8.22 (3.55)	8.09 (2.61)	9.99 (2.3)	11.13 (1.69)	9.61 (4.43)	9.62 (4.57)	9.32 (1.43)	8.77 (1.68)
Hip extension (Kgf)	16.82 (5.99)	17.74 (6.99)	20.25 (5.3)	23.67 (2.93)	19.3 (7.57)	21.25 (8.33)	22.74 (6.03)	19.76 (6.18)
Hip flexion (Kgf)	11.82 (2.82)	13.97 (3.55)	14.94 (2.99)	16.55 (3.53)	14.6 (4.95)	15.02 (4.49)	13.42 (3.29)	13.42 (4.03)
Hip internal rotation (Kgf)	8.48 (2.69)	8.59 (2.75)	8.88 (3.36)	10.99 (2.95)	9.61 (4.18)	10.4 (4.18)	10.0 (2.14)	9.14 (1.86)
Hip external rotation (Kgf)	7.44 (1.52)	7.76 (1.87)	7.15 (1.21)	8.9 (1.07)	8.03 (3.49)	8.15 (2.86)	7.72 (1.27)	7.09 (1.61)
Knee extension (Kgf)	20.86 (9.17)	25.26 (11.16)	30.38 (10.69)	35.23 (7.28)	32.07 (13.56)	31.85 (15.26)	39.71 (9.54)	36.76 (10.44)
Knee flexion (Kgf)	8.95 (4.02)	13.46 (11.02)	12.76 (4.09)	14.07 (3.02)	14.15 (7.08)	13.71 (5.07)	12.53 (3.46)	11.39 (2.98)

Isometric muscle strength assessment using a handheld dynamometer.

	Variables	Knee group (n = 25)		Hip and knee group (n = 25)	
		Pre (Baseline)	Post (12 weeks)	Pre (Baseline)	Post (12 weeks)
Şahin, et al. 2016 (not included in meta-analyses)	VAS resting (points)	3 (3–4)	2 (1.5–3)	3 (3–4)	1 (0–2)
	VAS standing (points)	5 (4–6)	3 (2–4)	4 (3–5)	1 (0.5–2)
	VAS walking (points)	5 (4–6)	4 (3–5)	5 (4–5.5)	2 (2–3)
	VAS running (points)	6 (5–7)	5 (4–6)	6 (5–7)	3 (3–5.5)
	VAS prolonged sitting (points)	5 (4–7)	4 (3.5–5)	5 (4–6.5)	3 (2–4.5)
	VAS kneeling (points)	6 (5–8)	5 (3.5–6)	6 (6–7)	3 (2–5)
	VAS squatting (points)	6 (4.5–7)	4 (4–5.5)	6 (5–7)	3 (2–4.5)
	VAS stairs (points)	6 (5.5–7)	5 (3.5–6)	7 (5.5–7)	3 (3–5)
	VAS ramp (points)	6 (5.5–7)	5 (4–6)	7 (6–7)	4 (3–4.5)
	3-limb hop (cm)	249.9 (53.4)	315.2 (57.1)	244.2 (59.8)	328.7 (71.4)
	One-leg squat (N/30s)	11 (10–13)	15 (13–17)	12 (9–13)	17 (15–19.5)
	Step-down (N/30s)	9 (8–11.5)	14 (13–15.5)	10 (8.5–11.0)	16 (13.5–17)
	Kujala Function Score (points)	72.4 (8.5)	77.9 (6.6)	71.4 (5.5)	83.0 (6.80
	(60°/s)	NI	80.1 (28.2)	NI	74.2 (23.7)
	(180°/s)		46.0 (15.6)		38.2 (17.1)
	(60°/s)		48.2 (11.3)		41.5 (13.4)
	(120°/s)		33.4 (11.8)		27.3 (10.7)
	Hip abduction (Nm)	39.2 (18.6)	NI	37.0 (16.5)	NI
	(60°/s)	NI	49.1 (15.4)	NI	48.7 (16.1)
	(120°/s)		22.5 (8.3)		23.5 (15.1)
	Hip external rotation (Nm)	27.3 (12.9)	NI	28.6 (10.3)	NI
	(30°/s)	NI	42.0 (14.4)	NI	41.9 (9.7)
	(60°/s)		33.0 (9.7)		42.1 (15.0)

*Median (IQR). Concentric muscle strength assessment using an isokinetic dynamometer.

Scafoglieri, et al. 2021

Variables	Exercise group (n=18)		Spinal manual therapy group (n=25)	
	**Pre (Baseline)**	**Post (6 weeks)**	**Pre (Baseline)**	**Post (6 weeks)**
Maximum pain VAS (mm)*!	73.6 (13.6)	59.7 (22.0)	74.4 (14.3)	36.3 (23.0)
Minimum pain VAS (mm)*	10.0 (4.3 –22.5)	6.5 (0.0 - 12.5)	7.0 (0.0 –17.0)	0.0 (0.0 - 1.0)
Current pain VAS (mm)*	27.0 (9.5 –48.0)	15.0 (3.8 - 28.5)	33.0 (18.0 –53.0)	2.0 (0.0 - 11.5)
AKPS (points)	60.4 (10.6)	70.9 (14.3)	66.9 (11.6)	83.3 (10.9)
Knee extension (N-m)*	121.5 (105.0 –154.5)	140.0 (111.0 - 167.0)	125.0 (103.3 –137.8)	127.0 (117.0 - 144.5)

*Median (IQR). Isometric muscle strength assessment using an isokinetic dynamometer.

Shahnaz, et al. 2012

Variables	EMG-BF (n=15)		Sham EMG-BF (n=15)	
	**Pre (Baseline)**	**Post (3 weeks)**	**Pre (Baseline)**	**Post (3 weeks)**
VAS (cm)	6.13 (0.81)	1.53 (0.74)	6.06 (1.03)	2.93 (0.88)
Knee extensors (quadriceps)	9.07 (1.87)	13.33 (1.89)	9.42 (1.86)	11.54 (1.70)

Isometric muscle strength assessment using a strain gauge.

Sheikhi, et al. 2024

Variables	Education plus trunk and hip exercises (n=30)		Trunk and hip exercise (n=30)	
	**Pre (Baseline)**	**Post (8 weeks)**	**Pre (Baseline)**	**Post (8 weeks)**
VAS (0-10)	6.10 (1.52)	3.41 (1.47)	5.70 (1.34)	3.93 (1.26)
KOS-ADL (points)	64.90 (8.91)	76.35 (6.56)	66.57 (7.48)	74.49 (6.03)
Hip abductors (BW)	16.19 (5.08)	21.41 (4.94)	17.24 (4.30)	20.76 (3.85)
Hip external rotators (BW)	12.07 (3.62)	18.04 (4.02)	13.54 (3.78)	17.54 (3.77)
Hip extensors (BW)	18.64 (4.27)	23.38 (4.27)	19.46 (3.93)	22.84 (3.87)
Knee extensors (BW)	25.18 (4.78)	32.03 (4.51)	26.51 (4.63)	31.05 (4.32)

Isometric muscle strength assessment using a handheld dynamometer.

	Variables	Strengthening group (n=30)		Neuromuscular training group (n=34)	
		Pre (Baseline)	Post (12 weeks)	Pre (Baseline)	Post (12 weeks)
Silva, et al. 2023	VAS pre-test (cm)	2.4 (3.0)	0.1 (0.8)	1.9 (2.1)	0.2 (0.5)
	VAS post-squat test (cm)	4.0 (3.2)	0.9 (1.4)	3.4 (2.4)	0.7 (1.2)
	VAS post-step test (cm)[!]	5.2 (3.2)	1.2 (2.0)	3.9 (2.6)	0.8 (0.9)
	ADLS (points)	41.1 (11.0)	56.5 (10.2)	43.7 (10.6)	60.7 (9.1)
	AKPS (points)[!]	62.3 (11.5)	78.9 (8.7)	65.2 (10.3)	82.0 (9.4)
	Knee extension (%)	407.5 (146.86)	447.9 (135.9)	376.7 (137.2)	420.9 (141.0)
	Hip external rotation (%)	122.7 (40.43)	140.3 (41.6)	119.3 (38.3)	133.7 (36.4)
	Hip abduction (%)	106.7 (50.95)	121.3 (37.1)	109.9 (28.8)	129.9 (37.0)
	Hip extension (%)	172.0 (94.3)	243.8 (96.2)	180.6 (113.0)	216.1 (94.4)

Isometric muscle strength assessment using a handheld dynamometer.

	Variables	Isometric exercises (n=41)		Somatosensory training (n=28)		Control (n=29)	
		Pre (Baseline)	Post (12 weeks)	Pre (Baseline)	Post (12 weeks)	Pre (Baseline)	Post (12 weeks)
Steinberg, et al. 2020	Mean VAS scale (points)*	4.56 (1.79)	4.03 (2.73)	5.04 (2.13)	4.45 (2.18)	5.36 (1.98)	5.00 (2.23)
	Mean VAS PIT (points)	4.64 (2.22)	3.36 (2.24)	4.83 (2.24)	3.17 (2.76)	4.51 (2.16)	6.15 (1.73)
	Knee flexion (N/kg)	2.56 (0.48)	2.72 (0.68)	2.76 (0.47)	2.95 (0.65)	2.80 (0.70)	2.80 (0.48)
	Knee extension (N/kg)	5.25 (1.28)	5.52 (1.48)	5.53 (0.95)	5.68 (0.98)	5.26 (1.16)	5.19 (0.92)
	Hip abduction (N/kg)	2.51 (0.47)	2.83 (0.79)	2.72 (0.48)	2.71 (0.64)	2.82 (0.64)	2.25 (0.42)
	Hip adduction (N/kg)	2.32 (0.57)	2.65 (0.68)	2.50 (0.52)	2.65 (0.70)	2.47 (0.67)	2.53 (0.55)

Isometric muscle strength assessment using a handheld dynamometer.

Talbot, et al. 2020

Variables	HEP (n=34)		NMES + HEP (n=33)		TENS + HEP (n=33)		NMES/TENS + HEP (n=30)	
	Pre (Baseline)	Post (9 weeks)	Pre (Baseline)	Post (9 weeks)	Pre (Baseline)	Post (9 weeks)	Pre (Baseline)	Post (9 weeks)
Current pain severity[1]	3.48 (1.84)	2.77 (2.22)	3.42 (1.66)	2.44 (1.79)	4.24 (1.76)	3.01 (2.33)	3.98 (1.77)	3.27 (2.26)
30-Second Chair Stand Test (repetitions)	11.6 (2.8)	15.3 (4.0)	11.0 (3.0)	14.4 (4.4)	11.0 (3.1)	13.4 (3.5)	11.0 (3.8)	15.1 (4.8)
Timed Stair Climb Test (seconds)	3.7 (1.1)	3.3 (0.7)	4.0 (1.4)	3.5 (1.2)	4.2 (1.4)	3.3 (1.0)	4.1 (1.6)	3.4 (0.9)
Forward Step-Down Test (repetitions)	13.2 (3.9)	17.7 (5.2)	11.3 (3.6)	16.3 (5.3)	10.8 (4.9)	14.9 (5.7)	11.2 (4.0)	18.0 (6.0)
Six-Minute Timed Walk Test (inches)	24612 (2964)	25059 (3073)	23931 (2772)	25892 (3300)	23999 (2979)	25847 (3763)	23174 (2638)	25227 (2655)
30-Second Chair Stand Test pain	2.9 (1.8)	2.8 (2.2)	2.8 (1.7)	2.5 (1.9)	3.9 (1.8)	2.6 (2.2)	3.6 (2.0)	2.6 (2.4)
Timed Stair Climb Test pain	2.7 (1.9)	2.7 (2.0)	2.7 (1.8)	2.0 (1.8)	3.5 (1.9)	2.7 (2.3)	3.0 (2.2)	1.9 (2.1)
Forward Step-Down Test pain	4.2 (1.5)	3.5 (2.2)	4.2 (2.2)	2.8 (2.1)	5.2 (1.9)	3.3 (2.1)	5.3 (2.1)	3.2 (2.5)
Six-Minute Timed Walk Test pain	3.2 (1.7)	3.0 (2.1)	2.7 (2.2)	2.3 (2.0)	4.5 (1.7)	2.9 (2.3)	3.6 (2.3)	2.7 (2.5)
Knee extension (kg-force)	41.58 (16.56)	41.78 (13.46)	37.74 (15.39)	46.22 (18.31)	37.14 (15.36)	45.44 (23.16)	40.42 (18.87)	46.89 (17.78)
Knee flexion (kg-force)	21.85 (7.61)	24.15 (9.67)	20.32 (7.35)	26.95 (8.17)	21.33 (7.13)	25.01 (9.11)	21.95 (9.13)	26.64 (10.02)

Isometric muscle strength assessment using a handheld dynamometer. The strength of knee flexors and extensors were evaluated.

Talbot, et al. 2023

Variables	NMES plus high blood flow restriction (n=42)		NMES plus low blood flow restriction (n=42)	
	Pre (Baseline)	Post (9 weeks)	Pre (Baseline)	Post (9 weeks)
Pain VAS at muscle tests (cm)	3.36 (2.3)	2.67 (2.4)	3.47 (2.0)	2.35 (2.3)
30-second chair stand test (repetitions)	11.5 (3.19)	14.8 (5.00)	10.8 (2.60)	13.9 (3.54)
Forward step-down test (repetitions)	14.5 (5.2)	19.5 (7.1)	12.0 (4.9)	16.9 (6.0)
Timed stair climb test (seconds)	4.62 (2.61)	3.99 (1.07)	4.35 (0.88)	3.76 (0.68)
6-minute walk test (inches)	21,727 (3,589)	23,156 (3,578)	22,059 (3,270)	23,573 (4,0221)
Knee extensor (kgf)	54.8 (18.7)	62.7 (24.1)	54.4 (22.8)	63.0 (20.6)
Knee flexor (kgf)	21.0 (8.5)	21.5 (7.7)	21.6 (7.9)	19.8 (5.4)
Hip posterolateral stabilizers (kgf/kg)	29.6 (12.4)	32.5 (13.2)	28.3 (11.5)	31.3 (11.3)

Isometric muscle strength assessment using a handheld dynamometer.

Thomeé, et al. 1997 (not included in meta-analyses)

Variables	Isometric muscle contraction		Concentric muscle contraction	
	**Pre (Baseline)**	**Post (12 weeks)**	**Pre (Baseline)**	**Post (12 weeks)**
Vertical jump test	Not informed			
Isometric knee extension (Nm)	130.6 (81-200)	147.6 (89-209)	151.6 (82-210)	161.5 (97-229)
Concentric knee extension 25º-50º (Nm)	87.0 (43-125)	100.0 (44-143)	100.8 (74-133)	112.4 (81-167)
Concentric knee extension 50º-75º (Nm)	113.8 (69-147)	130.3 (55-171)	133.2 (91-171)	144.5 (95-225)
Eccentric knee extension 25º-50º (Nm)	104.8 (48-186)	122.6 (57-164)	121.7 (42-174)	136.9 (82-187)
Eccentric knee extension 50º-75º (Nm)	122.1 (58-215)	141.0 (80-209)	147.7 (77-212)	159.5 (88-273)

Muscle strength assessment using an isokinetic dynamometer.

Vasconcelos, et al. 2024

Variables	Strength and power training (n=18)		Strength training (n=19)	
	**Pre (Baseline)**	**Post (12 weeks)**	**Pre (Baseline)**	**Post (12 weeks)**
Usual pain (NRS 0-10)	4.27 (1.39)	1.72 (1.52)	4.41 (1.45)	2.38 (1.96)
Worst pain previous week (NRS 0 -10)[!]	5.44 (1.83)	3.33 (2.35)	5.55 (2.27)	2.66 (2.65)
AKPS (points)	69.05 (12.57)	82.33 (12.71)	70.83 (9.93)	83.55 (13.34)
Hip extensor (Nm/kg)	1.05 (0.40)	1.64 (0.61)	1.40 (0.79)	1.93 (0.89)
Hip abductor (Nm/kg)	1.48 (0.44)	1.89 (0.50)	1.47 (0.63)	1.81 (0.52)
Knee extensor (Nm/kg)	1.38 (0.30)	1.46 (0.31)	1.37 (0.46)	1.50 (0.43)

Maximal isometric muscle torque assessment using an isokinetic dynamometer.

Witvrouw, et al. 2000 (not included in meta-analyses)

Variables	Open kinetic chain (n=30)		Closed kinetic chain (n=30)	
	**Pre (Baseline)**	**Post (5 weeks)**	**Pre (Baseline)**	**Post (5 weeks)**
Kujala (points)	Not informed			
VAS (100 nm) daily activity				
VAS (100 nm) triple-jump test				
Knee extension 60º/sec				
Knee extension 180º/sec				

Knee extension 300º/sec
Knee flexion 60º/sec
Knee flexion 180º/sec
Knee flexion 300º/sec

Concentric muscle strength assessment using an isokinetic dynamometer.

Won, et al. 2025 (not included in meta-analyses)

**Variables**	**Lumbar spinal manipulation (n=15)**		**Placebo group (n=15)**	
	**Pre (Baseline)**	**Post (4 weeks)**	**Pre (Baseline)**	**Post (4 weeks)**
PPT – Rectus femoris	37.06 (6.73)	61.36 (7.33)	34.11 (8.58)	41.78 (8.91)
PPT – Vastus medialis	31.52 (9.47)	55.20 (11.51)	31.56 (7.02)	39.00 (8.51)
PPT – Vastus lateralis	33.23 (7.52)	58.08 (8.82)	32.23 (9.12)	38.55 (9.03)
Knee extension (N)	334.20 (40.08)	375.20 (41.87)	309.53 (48.10)	331.77 (48.10)

Isometric muscle strength assessment using a traction dynamometer.

Yalfani, et al. 2025

**Variables**	**Foot strike pattern + lower extremity strengthening (n=20)**		**Control group (n=20)**	
	**Pre (Baseline)**	**Post (8 weeks)**	**Pre (Baseline)**	**Post (8 weeks)**
VAS (cm)	4.65 (1.04)	1.55 (0.75)	4.05 (0.99)	6.10 (1.25)
Hip extension (BW)	32.49 (4.49)	40.21 (5.39)	32.65 (2.24)	30.38 (1.70)
Hip abduction (BW)	19.78 (3.03)	25.23 (3.71)	19.38 (1.94)	17.04 (1.42)
Knee extension (BW)	42.30 (5.66)	51.60 (4.61)	42.58 (4.39)	38.60 (4.76)

Isometric muscle strength assessment using a handheld dynamometer.

Yang, et al. 2026

Variables	Exercise + EMS (n=22)		Exercise only (n=20)	
	**Pre (Baseline)**	**Post (6 weeks)**	**Pre (Baseline)**	**Post (6 weeks)**
VAS (cm)	4.7 (0.7)	2.43 (0.85)	4.6 (0.9)	2.40 (0.75)
AKPS (points)	77.0 (7.3)	85.80 (7.89)	75.7 (5.3)	79.18 (5.05)
Knee flexion 60°/sec (Nm/kg)	0.88 (0.56)	1.31 (0.51)	0.87 (0.66)	1.41 (0.52)
Knee extension 60°/sec (Nm/kg)	2.15 (0.58)	3.03 (0.47)	2.24 (0.70)	2.65 (0.61)
Hip extension 60°/sec (Nm/kg)	2.13 (1.17)	2.88 (1.13)	2.15 (0.89)	2.97 (0.86)
Hip abduction 60°/sec (Nm/kg)	1.00 (0.33)	1.39 (0.44)	1.16 (0.55)	1.30 (0.26)
Hip external rotation 60°/sec (Nm/kg)	0.41 (0.16)	0.76 (0.32)	0.44 (0.14)	0.96 (0.31)

Concentric muscle strength assessment using an isokinetic dynamometer.

Yelvar, et al. 2015

Variables	Therapeutic Knee Exercises and Stabilization (n=22)		Therapeutic Knee Exercises (n=20)	
	**Pre (Baseline)**	**Post (6 weeks)**	**Pre (Baseline)**	**Post (6 weeks)**
VAS (Points)	7.54 (1.68)	3.02 (1.54)	7.65 (1.69)	5.50 (1.76)
Kujala (Points)	48.00 (12.54)	82.45 (13.02)	49.35 (11.23)	66.75 (16.19)
One leg hop (cm)	54.38 (22.53)	75.61 (20.09)	48.65 (24.75)	64.30 (23.52)
Timed get up & go test (sec)	3.86 (0.79)	3.37 (0.55)	4.33 (0.78)	3.89 (0.50)
Knee extension 60°/sec (Nm/kg)[!]	0.85 (0.31)	1.04 (0.32)	0.85 (0.46)	0.99 (0.47)
Knee extension 180°/sec (Nm/kg)	0.69 (0.29)	0.83 (0.27)	0.64 (0.25)	0.79 (0.28)
Knee flexion 60°/sec (Nm/kg)[!]	0.70 (0.22)	0.88 (0.19)	0.62 (0.27)	0.69 (0.26)
Knee flexion 180°/sec (Nm/kg)	0.64 (0.25)	0.83 (0.14)	0.51 (0.21)	0.66 (0.18)

Concentric muscle strength assessment using an isokinetic dynamometer.

Yip, et al. 2006

Variables	EMG + exercise group (n=13)		Exercise-only group (n=13)	
	Pre (Baseline)	Post (8 weeks)	Pre (Baseline)	Post (8 weeks)
Patellofemoral Pain Severity Scale (%)	41.99 (18.6)	35.49 (22.7)	39.99 (19.6)	29.99 (21.2)
Knee extension 120º/sec (Nm/kg)	114.29 (86.4)	138.19 (85.8)	86.89 (66.8)	116.19 (69.7)

Concentric muscle strength assessment using an isokinetic dynamometer.

Abbreviations: ADLS: Activities of Daily Living; AKPS/AKP: Anterior Knee Pain Scale; BW: Body Weight; EMG-BF: Electromyographic Biofeedback; EMS: Electric Muscle Stimulation; KgF: Kilogram-Force; KOS-ADL: Knee Outcome Survey – Activities of Daily Living Scale; LEFS: Lower Extremity Functional Scale; LKSS: Lysholm Knee Scoring Scale; Nm/kg: Newton-meter/ Kilogram; NMES: Neuromuscular Electrical Stimulation; NPRS: Numeric Pain Rating Scale; PPT: Pressure Pain Threshold; VAS: Visual Analog Scale; WOMAC: Western Ontario and McMaster Universities Osteoarthritis Index.

Supplementary Material I. Cluster analysis graphs.

Changes in Pain versus Changes in Knee Extension Strength

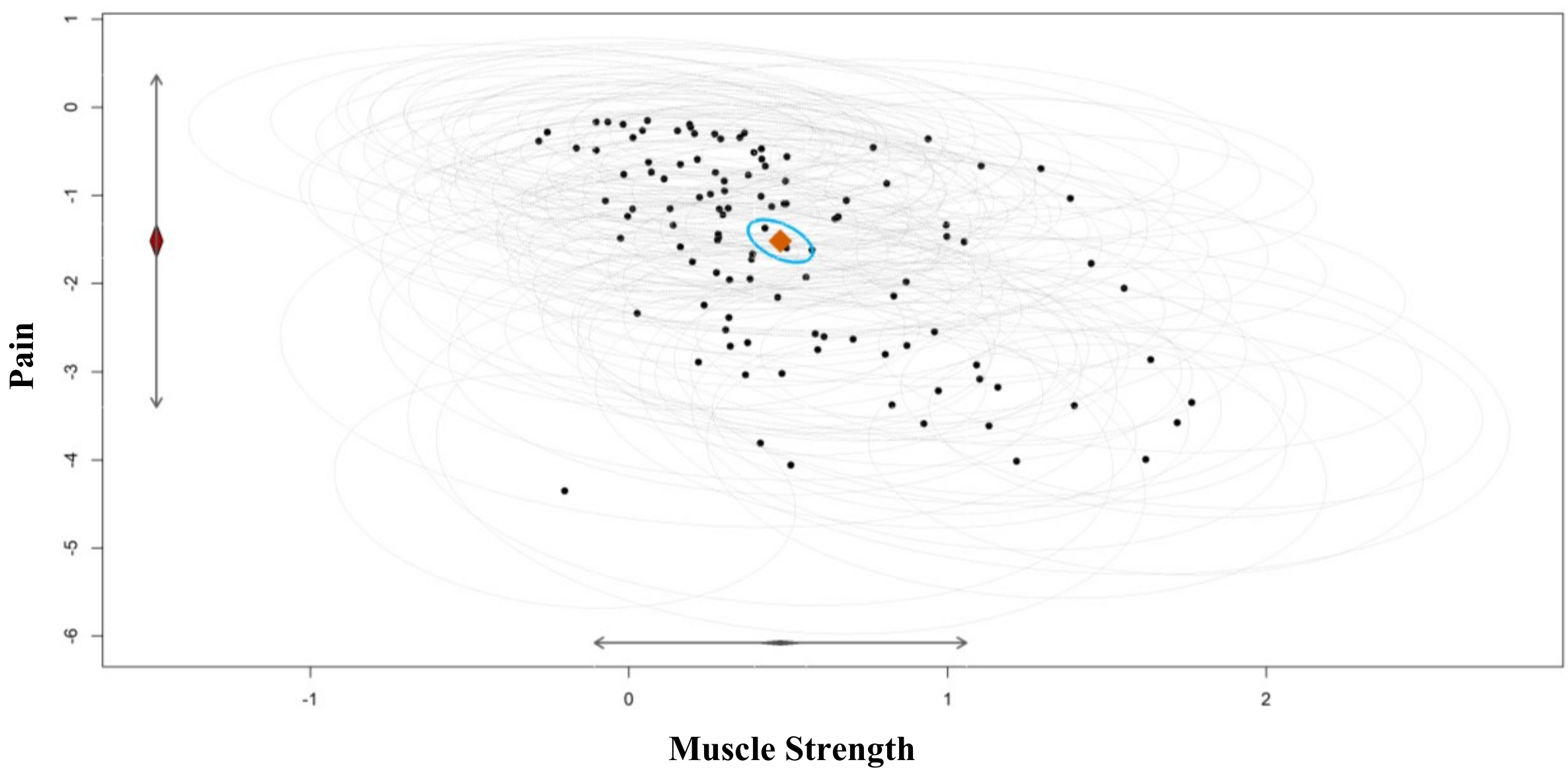

Outliers (clusters excluded): Abdelmohsen (exercise), Alonazi (EMG-BF), Arslan (control group), Aytar (both clusters), Constantinou (flow restriction), Dolak (quadriceps group), Hammami (both clusters), Hansen (hip focused group), Hasan (EMG-BF), Hasan 2025 (placebo), Herrington (control group), Kamel (short foot exercises plus hip and knee exercises), Keet (crossover), Kurt (sham kinesiotape), Lee (dynamic stretching group), Melo (KT with tension), Rodrigues (sham stimulus plus resistance training), Saad (stretching group), Shahnaz (EMG-BF), Yalfani (control group).

Changes in Pain versus Changes in Knee Flexion Strength

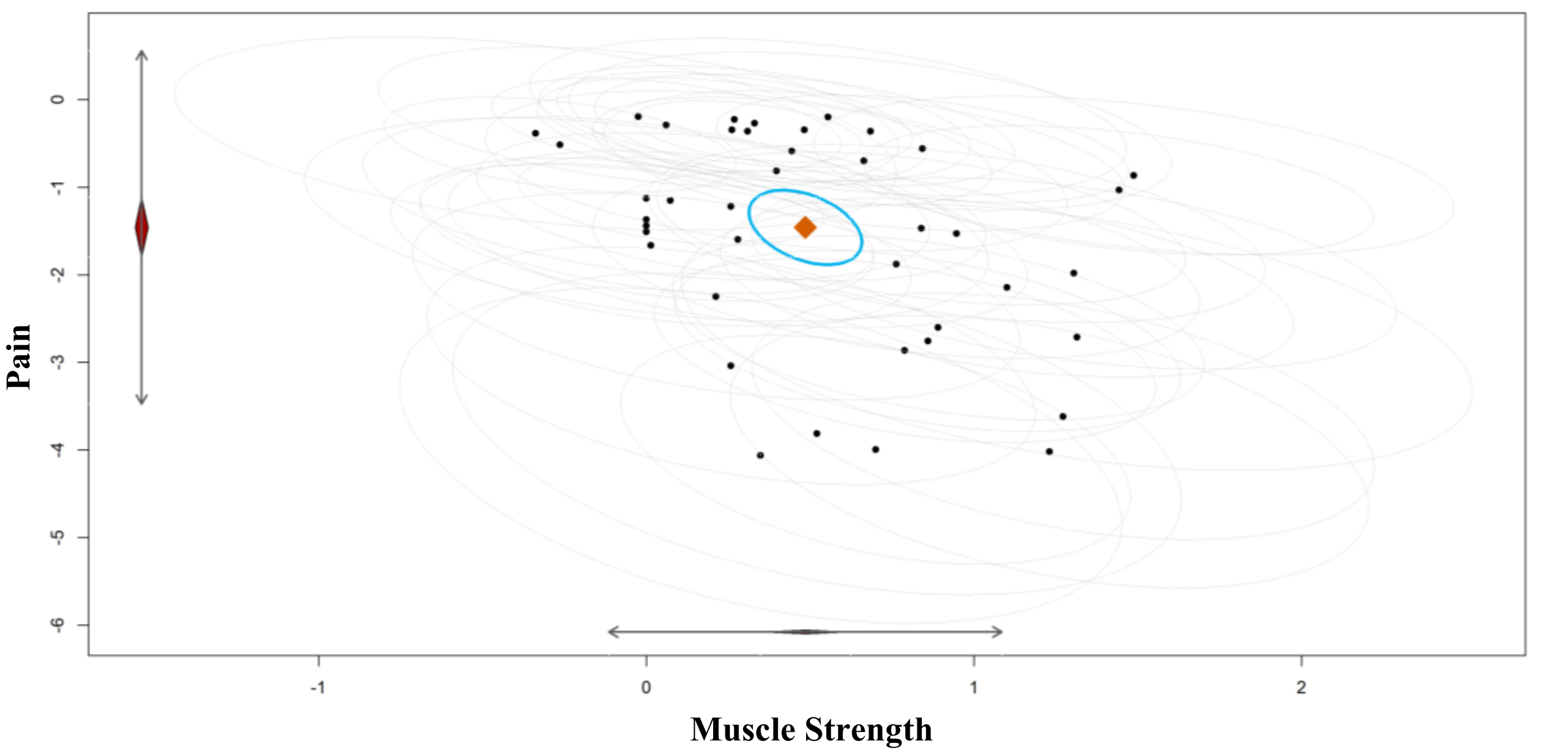

Outliers (clusters excluded): Abdelmohsen (exercise), Arslan (control group), Hammami (both clusters), Hansen (hip-focused exercise), Kurt (sham kinesiotape), Saad (stretching group and control group), Steinberg (control).

Changes in Pain versus Changes in Hip Abduction Strength

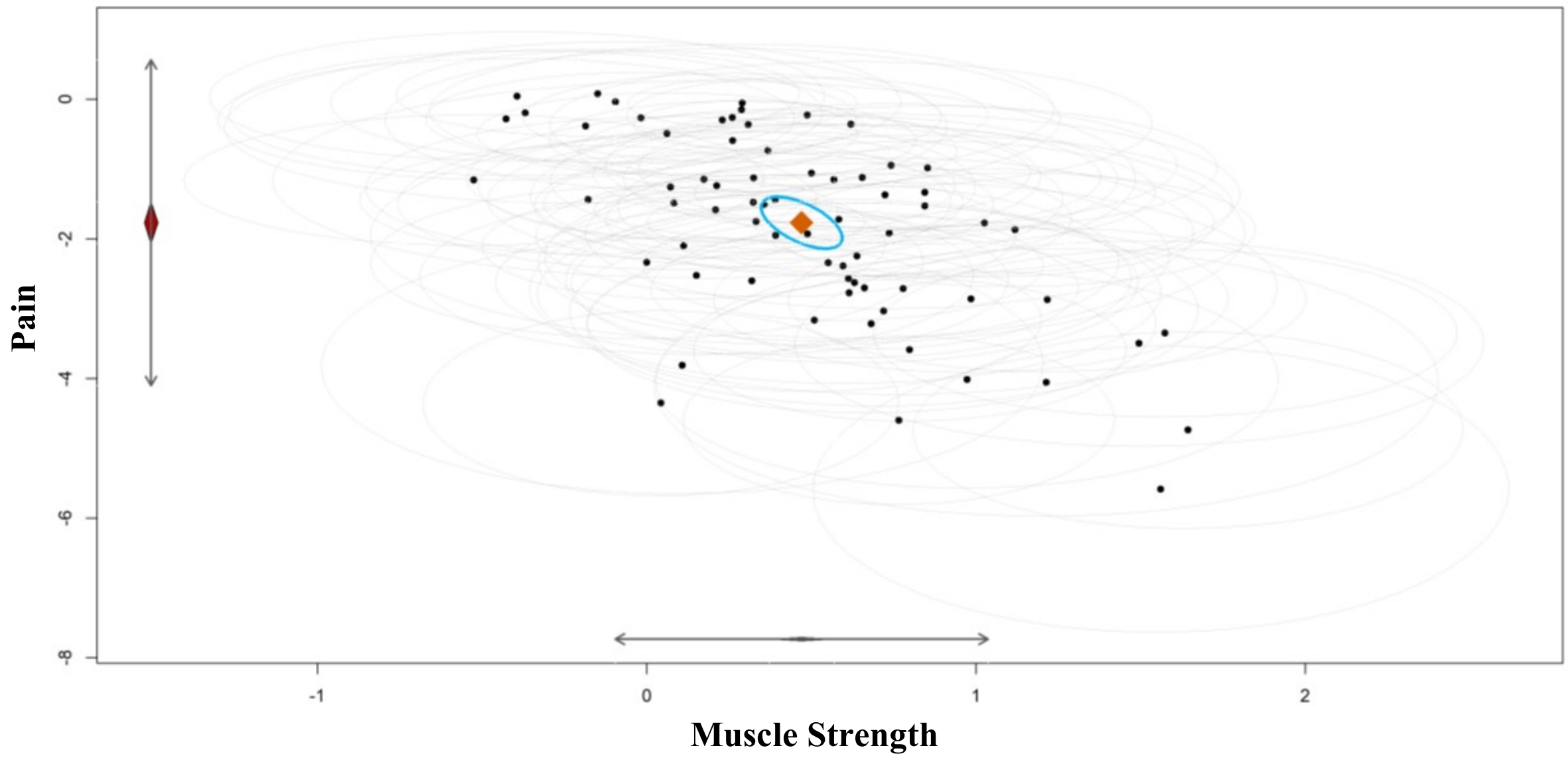

Outliers (clusters excluded): Kamel (hip and knee exercises), Kumar (both clusters), Mahmoud (both clusters), Saad (stretching group), Steinberg (control), Yalfani (control group).

Changes in Pain versus Changes in Hip Adduction Strength

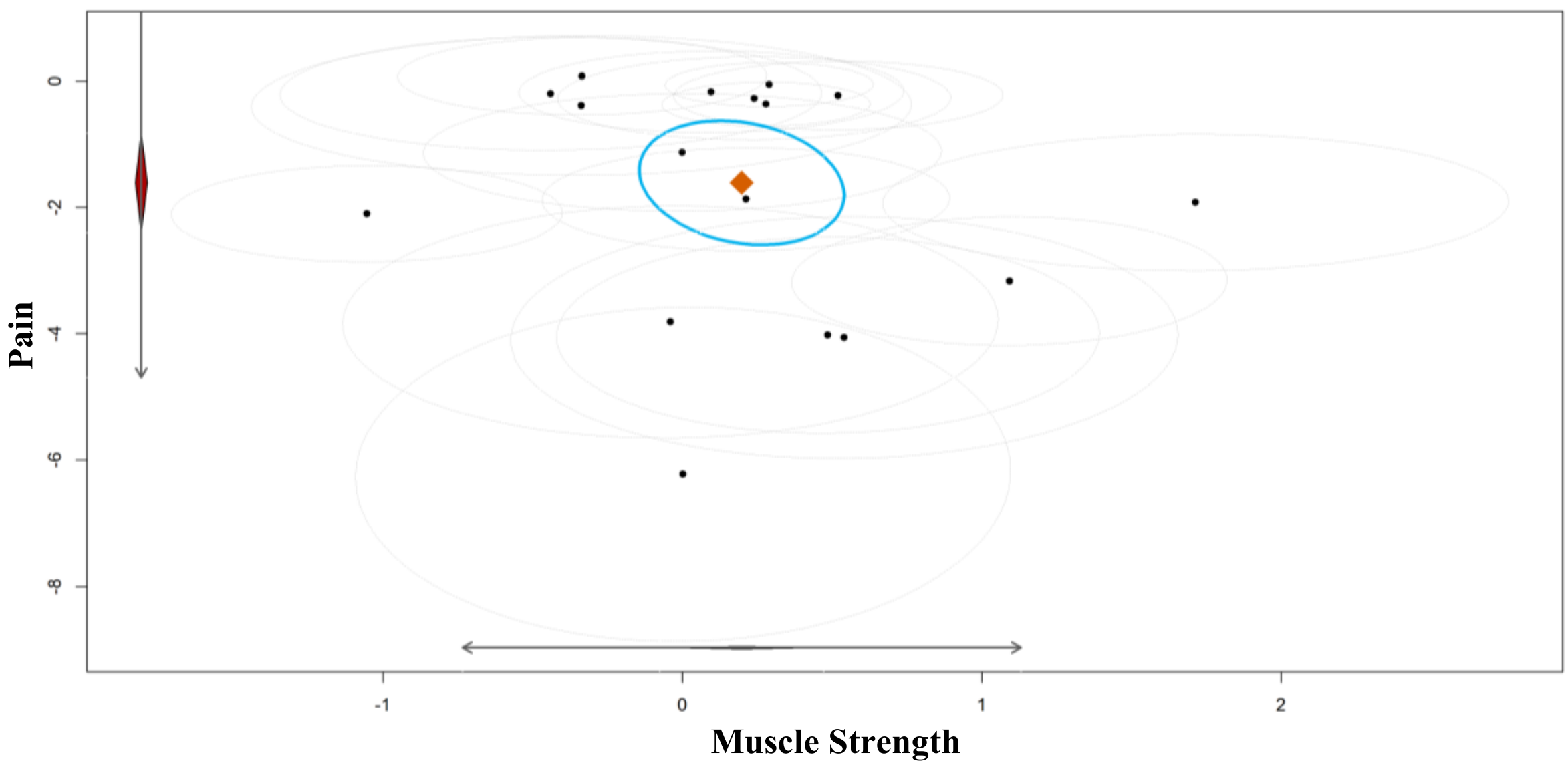

Outliers (clusters excluded): no cluster was excluded to allow the model building.

Changes in Pain versus Changes in Hip External Rotation Strength

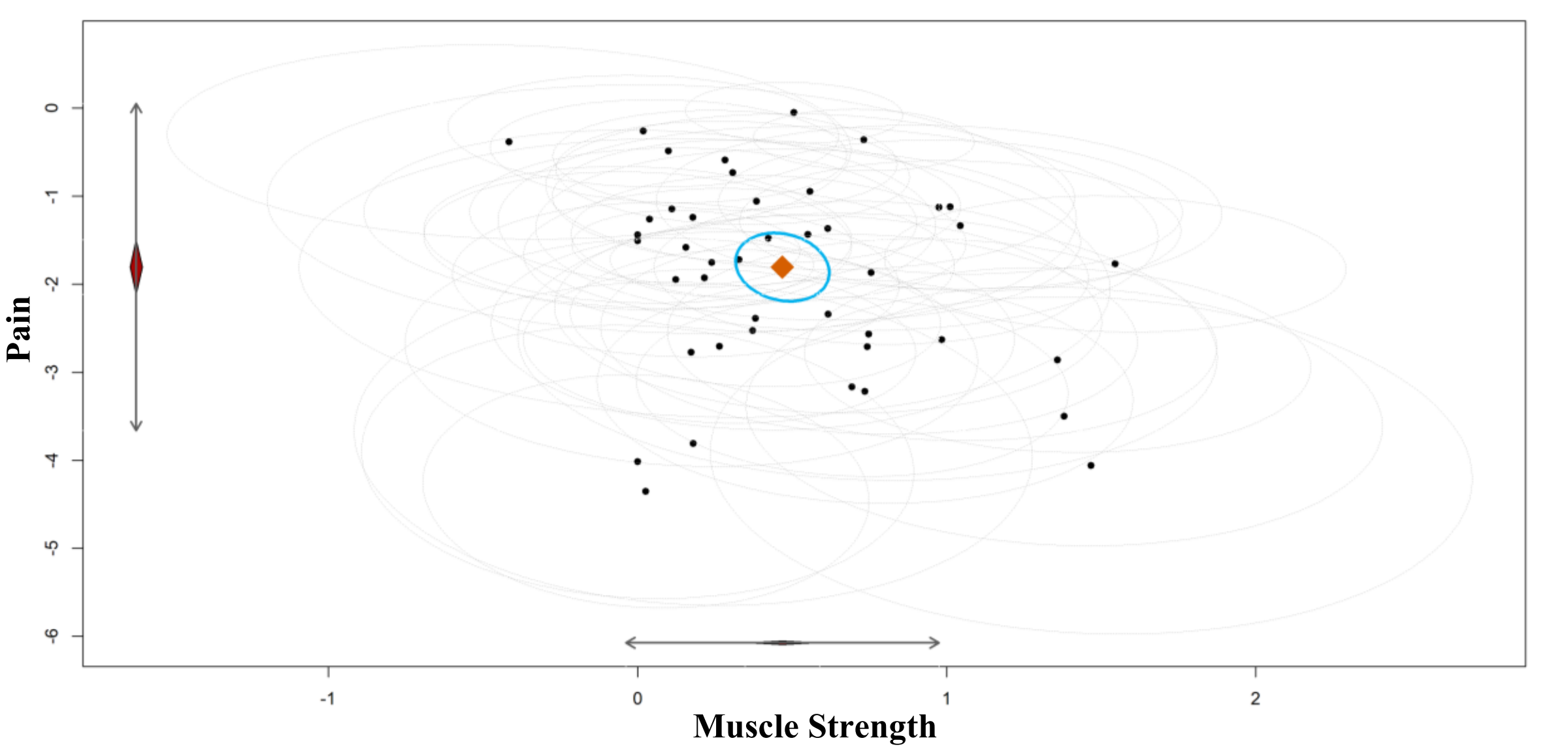

Outliers (clusters excluded): Aghakeshizadeh (internal focus group and external focus groups), Dolak (quadriceps group), Emamvirdi (both clusters), Khayambashi (control group), Saad (stretching group), Yang (exercise only group).

Changes in Pain versus Changes in Hip Internal Rotation Strength

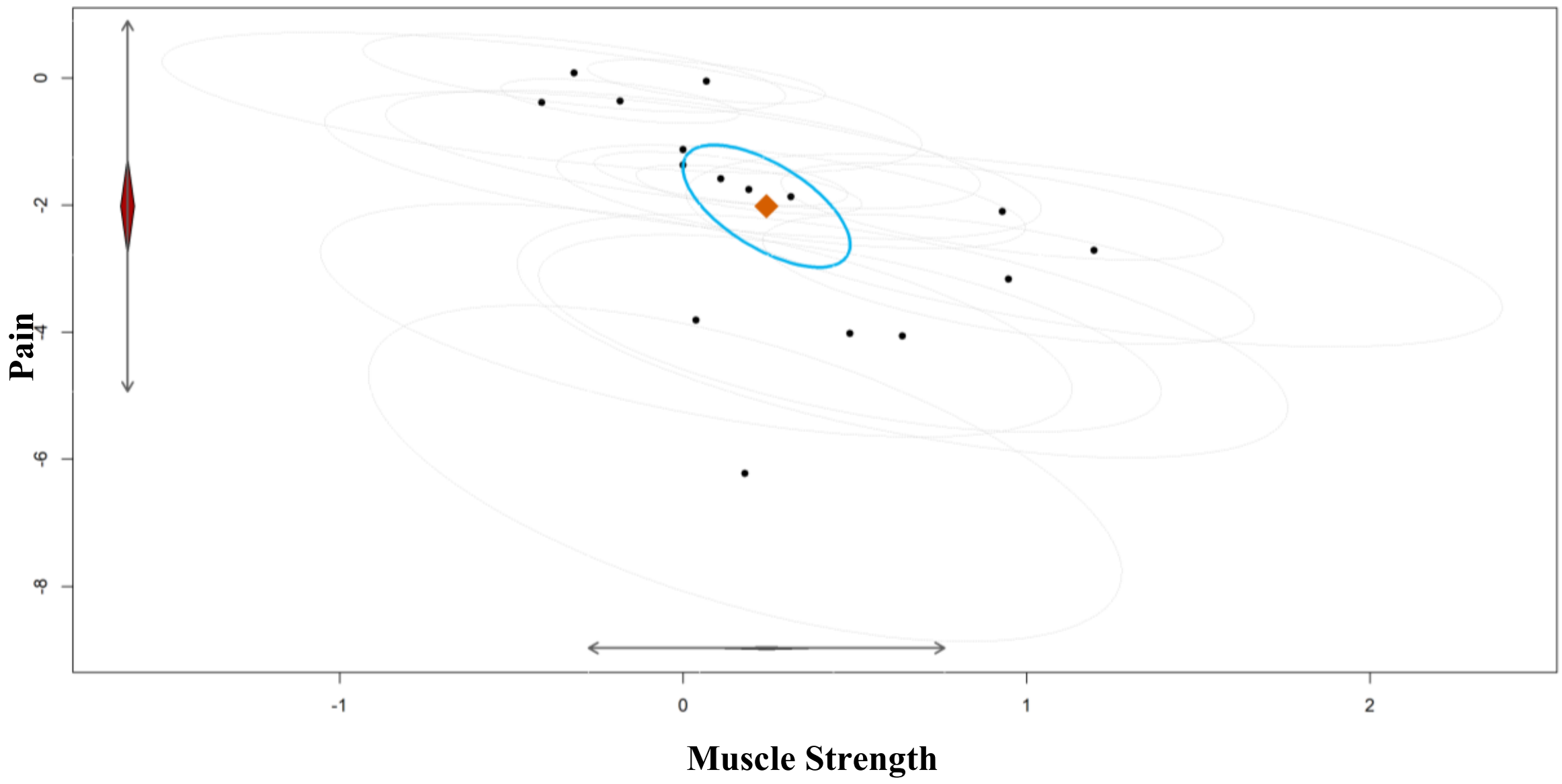

Outliers (clusters excluded): none

**Changes in <u>Pain</u> versus Changes in <u>Hip Extension</u> Strength**   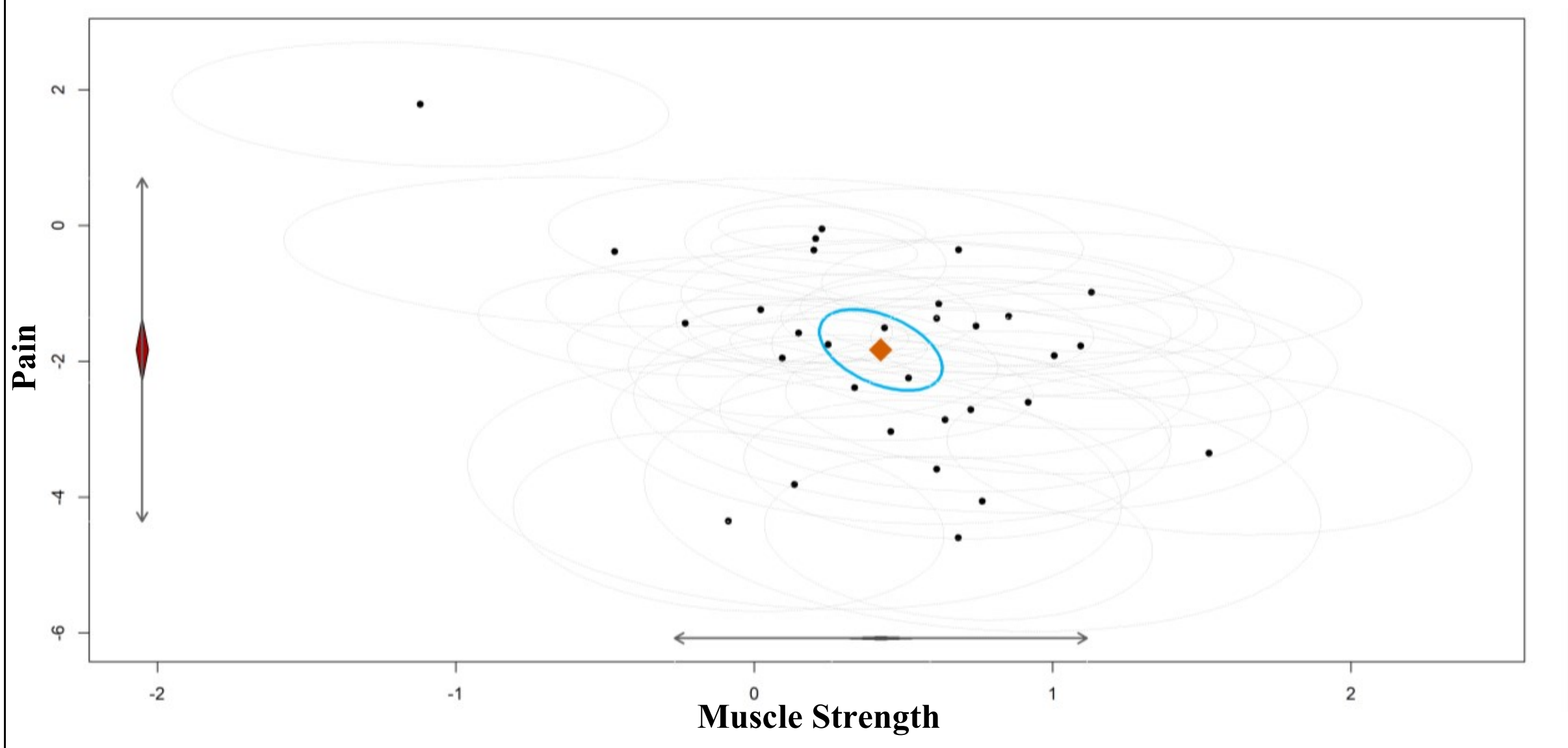   Outliers (clusters excluded): Klsaclk (short foot exercises), Saad (stretching group).
**Change in <u>Pain</u> versus <u>Change in General Strength</u>** (graph in the main text) <br>Outliers (clusters excluded): Alonazi (EMG-BF), Aytar (sham kinesiotape), Hammami (both clusters), Hasan (quadriceps group), Hasan 2025 (taping group), Herrington (control group), Kamel (short foot exercises plus hip and knee exercises), Kumar (both clusters), Liu & Wang (both clusters), Mahmoud (balance group), Melo (kinesiotape with tension), Rodrigues (sham stimulus plus resistance training), Saad (stretching group), Shahnaz (EMG-BF), Yalfani (control group).

Changes in Function versus Changes in Knee Extension Strength

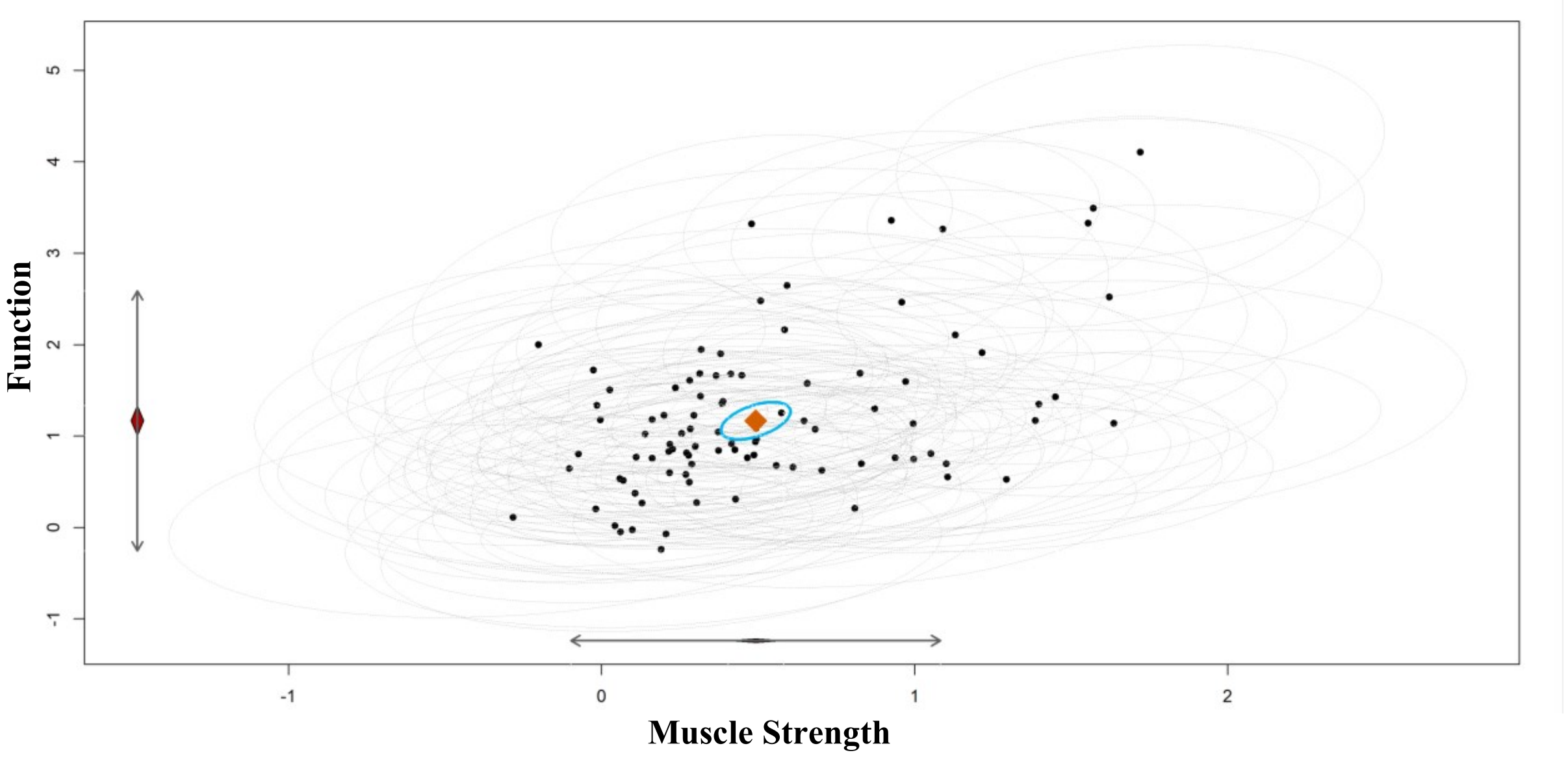

Outliers (clusters excluded): Abdelmohsen (exercise group), Alonazi (EMG-BF), Celik (standardized rehabilitation program), Hasan (EMG-BF), Herrington (weight-bearing group and control group), Kamel (short foot exercises plus hip and knee exercises), Lee 2021 (dynamic stretching group), Saad (control group).

Changes in Function versus Changes in Knee Flexion Strength

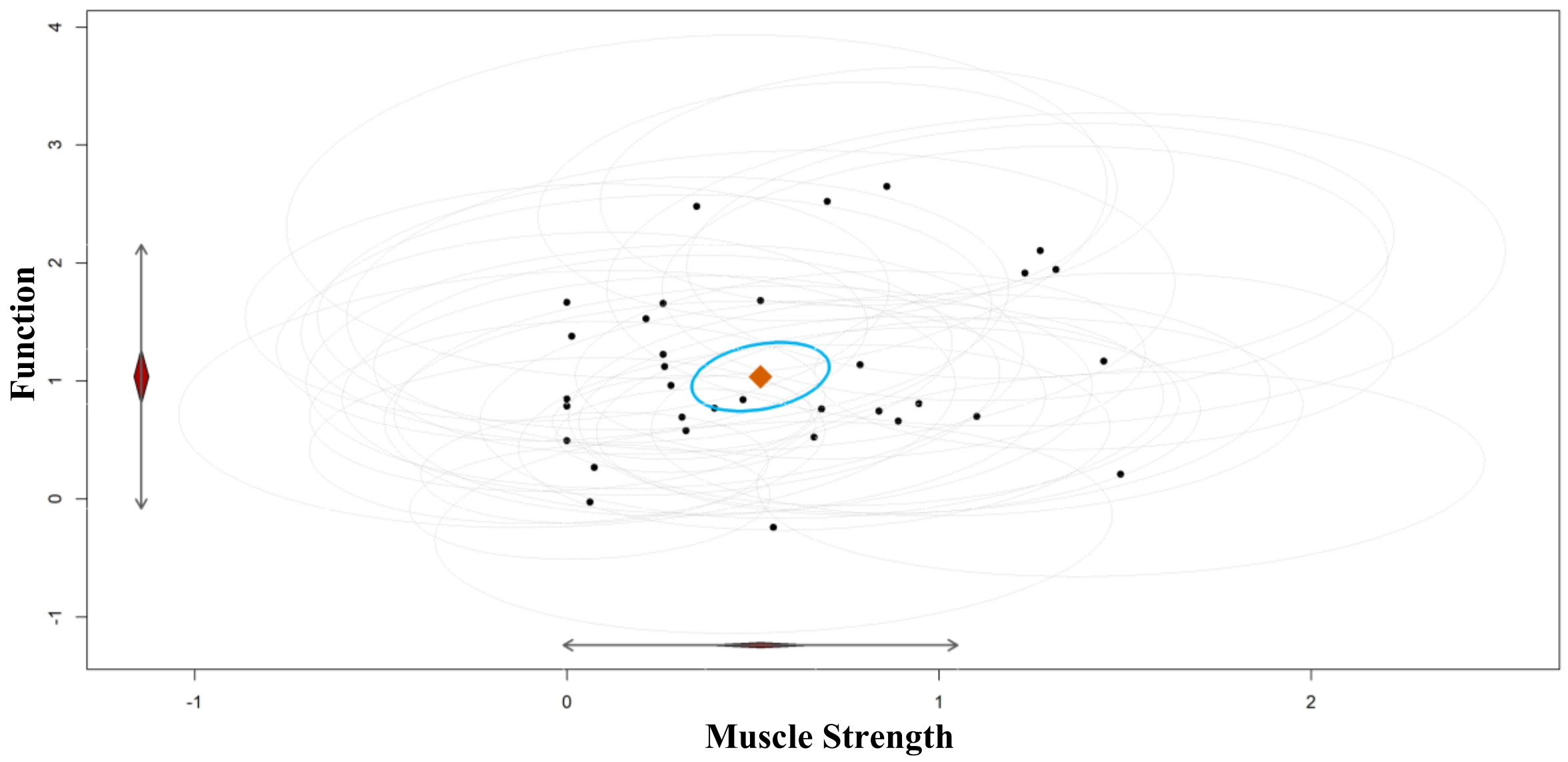

Outliers (clusters excluded): Abdelmohsen (exercise group), Arslan (control group), Hong (health education), Saad (stretching group and control group).

Changes in Function versus Changes in Hip Abduction Strength

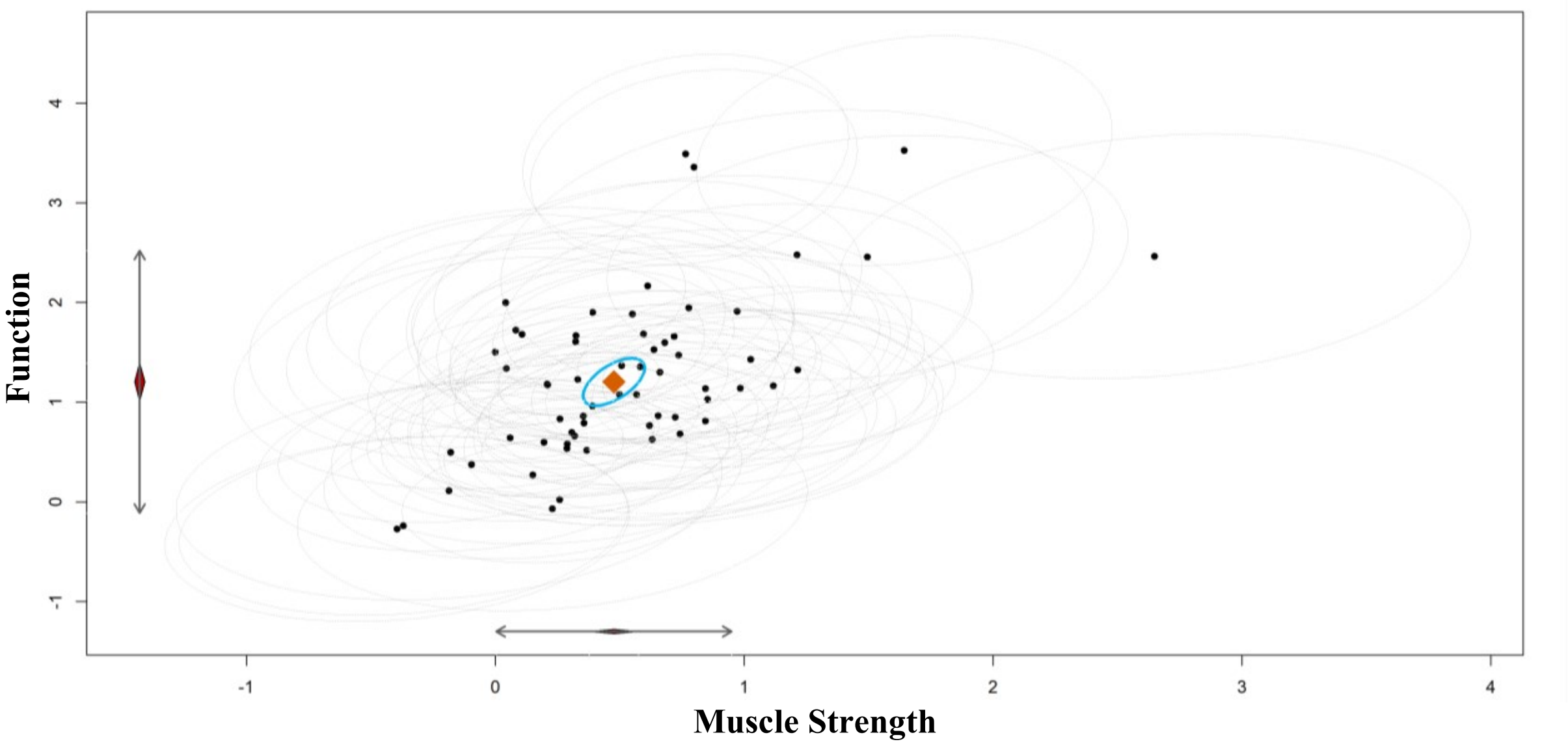

Outliers (clusters excluded): Kamel (short foot exercises plus hip and knee exercises), Kumar (both clusters).

Changes in Function versus Changes in Hip Adduction Strength

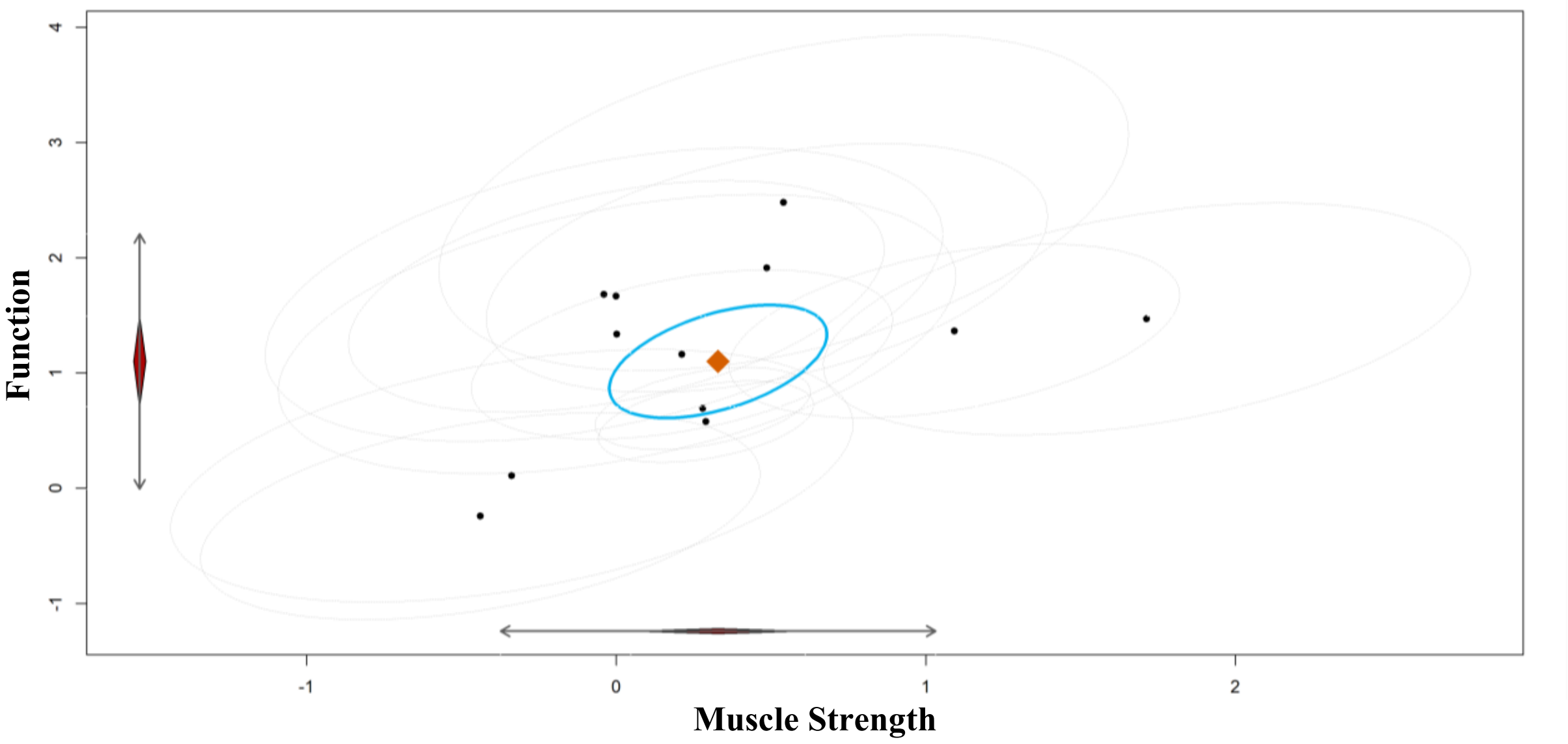

Outliers (clusters excluded): none

Changes in Function versus Changes in Hip External Rotation Strength

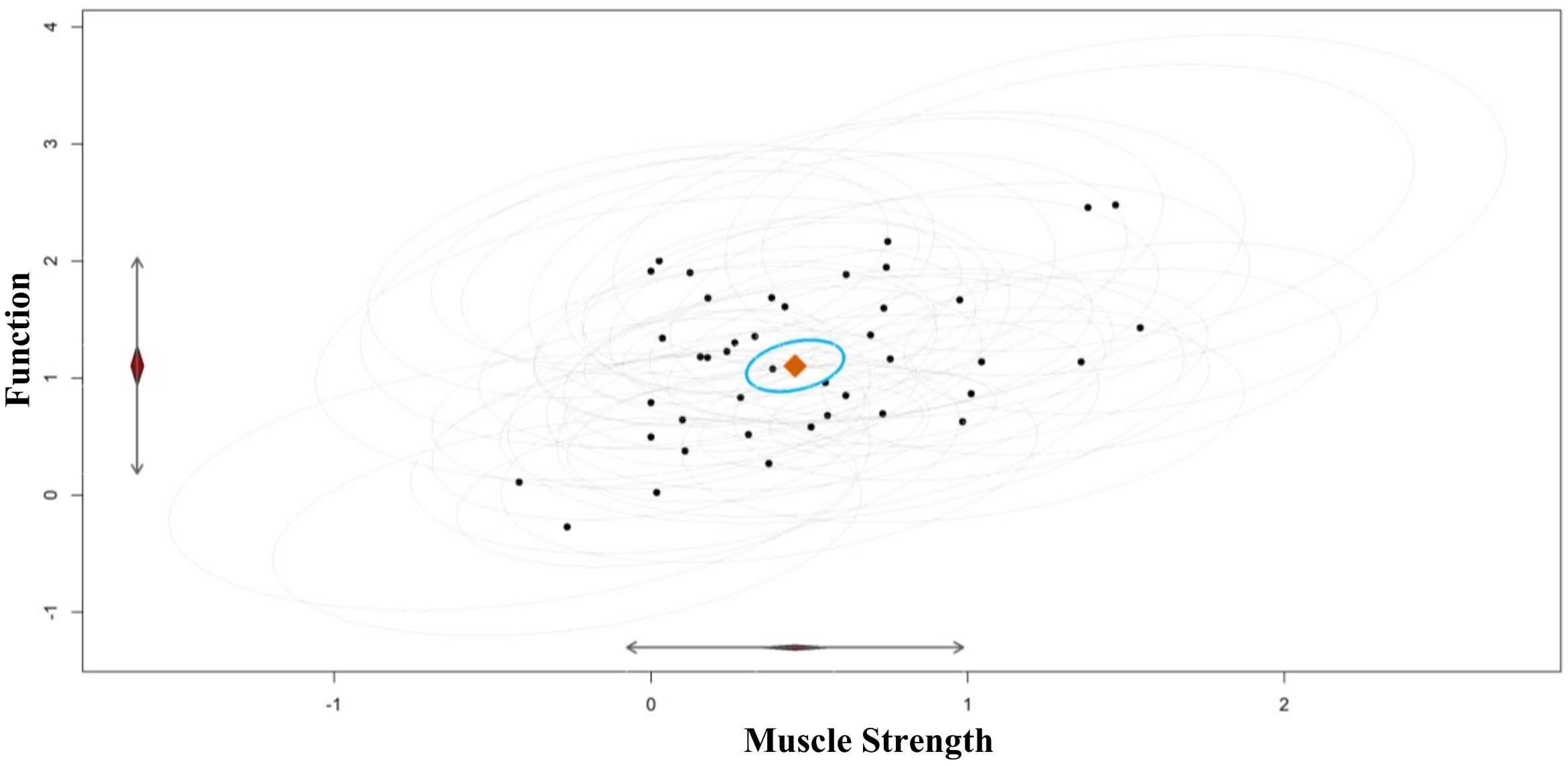

Outliers (clusters excluded): Aghakeshizadeh (internal focus and external focus), Yang (exercise only group).

Changes in Function versus Changes in Hip Internal Rotation - Isometric Strength

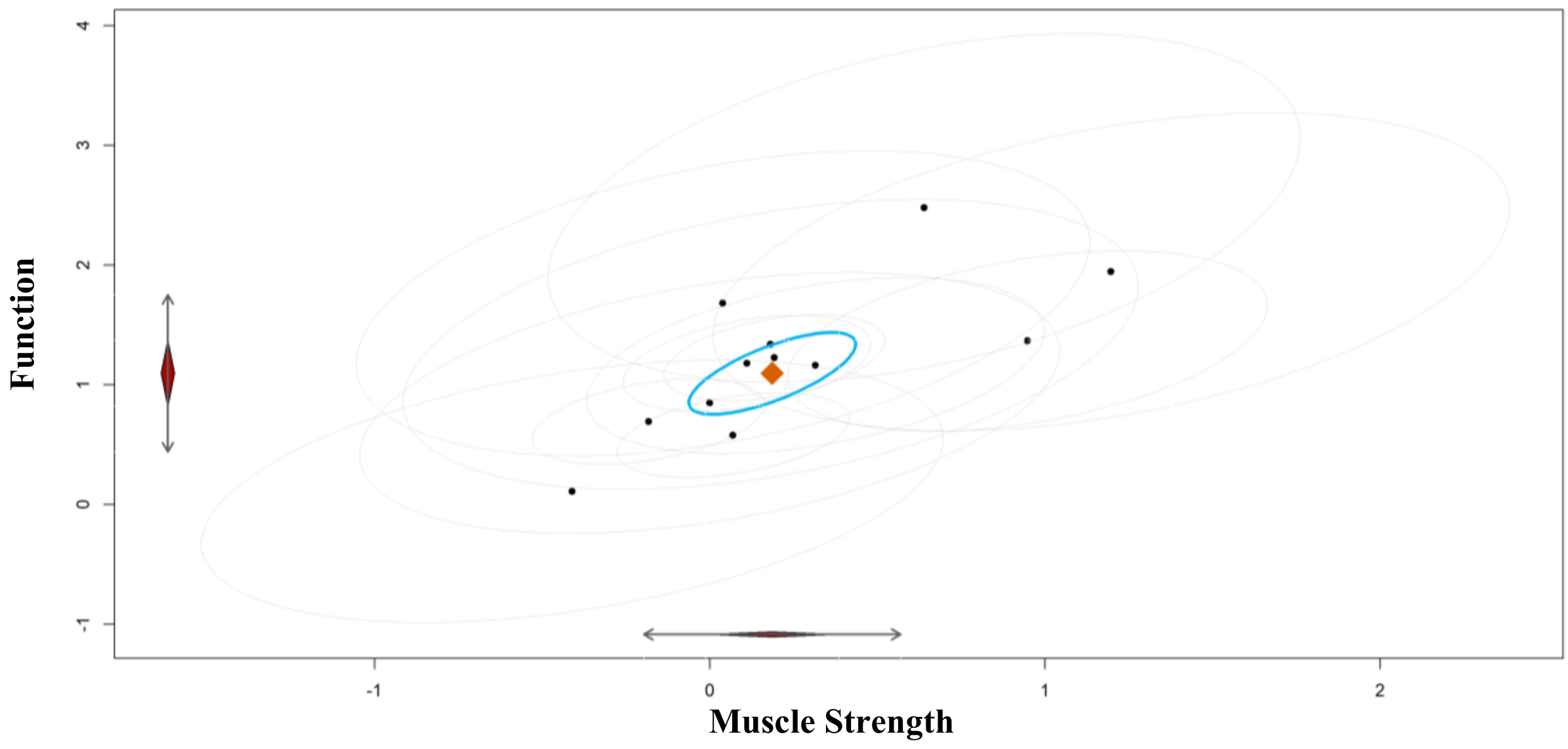

Outliers (clusters excluded): none

Changes in <u>Function</u> versus Changes in <u>Hip Extension</u> Strength

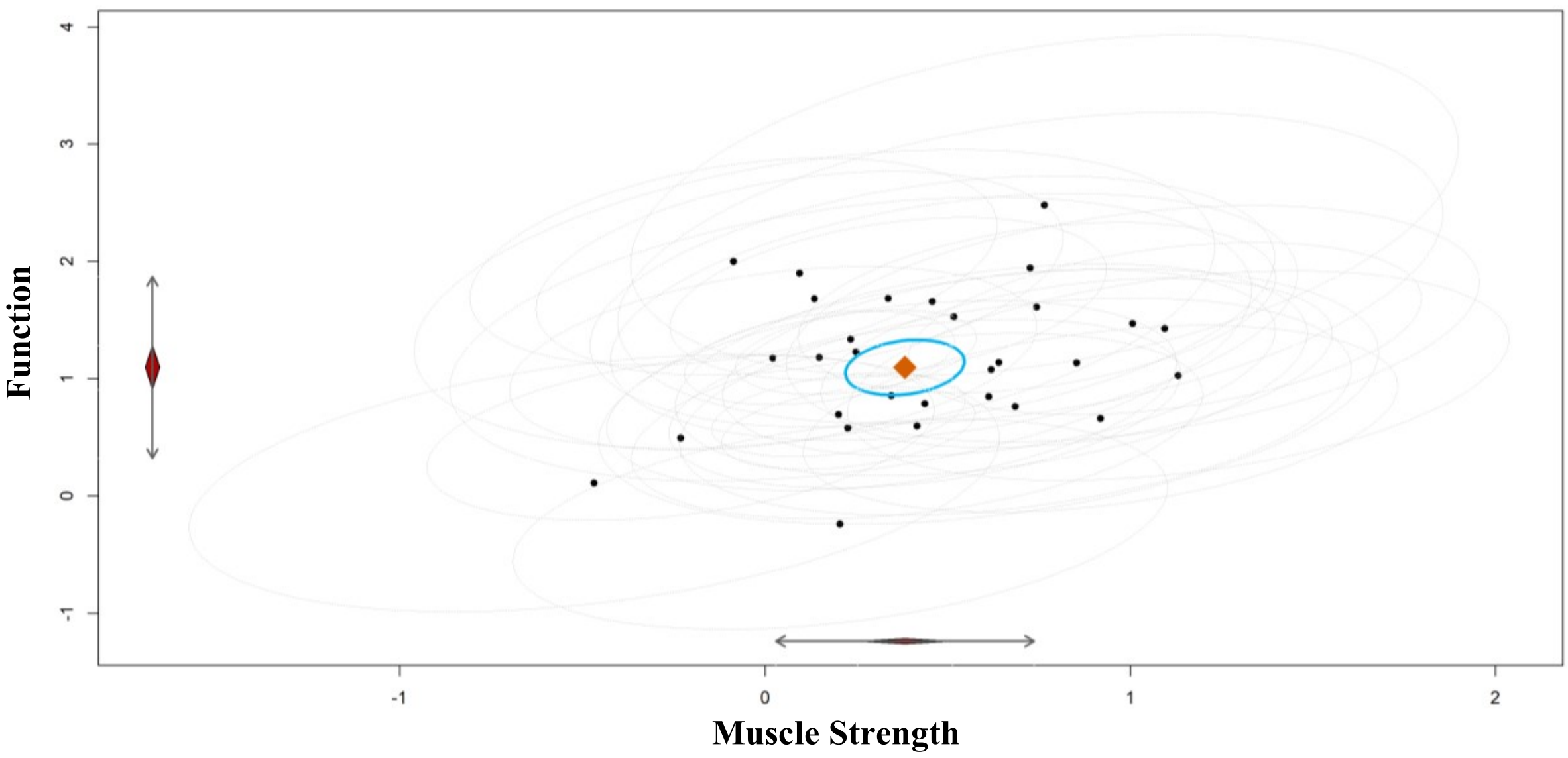

Outliers (clusters excluded): Constantinou (both clusters), Klsaclk (short foot exercise group).

Change in <u>Function</u> versus Change in <u>General Strength</u> (graph in the main text)

Outliers (clusters excluded): Alonazi (EMG-BF), Celik (standardized rehabilitation program), Hasan (EMG-BF), Hasan 2025 (taping group), Herrington (weight bearing group and control group), Kumar (both clusters), Lee 2021 (dynamic stretching group), Liu & Wang (both clusters).

Supplementary Material J. GRADE approach for evidence quality assessment.

Meta-Analysis	Risk of Bias	Inconsistency	Indirectness	Imprecision	Publication Bias	Quality of Evidence
Pain versus Muscle Strength						
Knee Extension						⊕⊕⊕⊖
Knee Flexion						⊕⊕⊖⊖
Hip Abduction						⊕⊕⊕⊖
Hip Adduction						⊕⊕⊖⊖
Hip External Rotation						⊕⊕⊕⊖
Hip Internal Rotation						⊕⊕⊕⊖
Hip Extension						⊕⊕⊖⊖
General						⊕⊕⊖⊖
Function versus Muscle Strength						
Knee Extension						⊕⊕⊕⊖
Knee Flexion						⊕⊕⊕⊖
Hip Abduction						⊕⊕⊕⊖
Hip Adduction						⊕⊕⊕⊖
Hip External Rotation						⊕⊕⊕⊕
Hip Internal Rotation						⊕⊕⊕⊕
Hip Extension						⊕⊕⊕⊖
General						⊕⊕⊖⊖

Legends

No serious concern

Serious concern (–1 level)

⊕⊕⊕⊕	High
⊕⊕⊕⊖	Moderate
⊕⊕⊖⊖	Low
⊕⊖⊖⊖	Very Low

Supplementary Material K. Subgroup analyses.

- A: Subgroup analyses for the associations between changes in Pain versus Muscle Strength.

Muscle group	k Clusters (n)	Pain Level [ES (CI 95%, I²)]	Muscle Strength [ES (CI 95%, I²)]	Correlational coefficient [r (CI 95%)]	Regression coefficient [β (CI 95%)]	Quality of Evidence
Knee Extension						
Physical Exercise interventions	63 (n=1,557)	-1.73 (-1.99, -1.47) I² = 89%	0.54 (0.43, 0.64) I² = 48%	-0.76 (-0.78, -0.74)	-0.20 (-0.22, -0.18)	Moderate
Endurance training	37 (n=793)	-1.42 (-1.71, -1.13) I² = 85%	0.59 (0.44, 0.74) I² = 51%	-0.69 (-0.72, -0.65)	-0.18 (-0.22, -0.15)	Low
Strength training	27 (n=798)	-1.97 (-2.33, -1.62) I² = 88%	0.51 (0.36, 0.65) I² = 42%	-0.93 (-0.94, -0.92)	-0.18 (-0.19, -0.17)	Moderate
Power training	1 (n=18)	Data did not permit the analysis				
Multimodal programmes	34 (n=734)	-1.57 (-1.91, -1.20) I² = 89%	0.54 (0.39, 0.70) I² = 49%	-0.48 (-0.53, -0.42)	-0.14 (-0.20, -0.09)	Moderate
Non-Exercise-Based interventions	15 (n=303)	Data did not permit the analysis				
Knee Flexion						
Physical Exercise interventions	24 (n=551)	-1.70 (-2.19, -1.22) I² = 92%	0.55 (0.38, 0.72) I² = 41%	-0.25 (-0.33, -0.17)	-0.06 (-0.15, 0.01)*	Moderate
Endurance training	15 (n=334)	Data did not permit the analysis				
Strength training	10 (n=263)	-1.88 (-2.49, -1.27) I² = 87%	0.44 (0.16, 0.672) I² = 51%	-0.82 (-0.86, -0.78)	-0.22 (-0.26, -0.18)	Low
Multimodal programmes	13 (n=314)	-1.34 (-1.86, -0.82) I² = 88%	0.49 (0.23, 0.76) I² = 60%	-0.68 (-0.74, -0.62)	-0.21 (-0.27, -0.15)	Low
Non-Exercise-Based interventions	4 (n=99)	Data did not permit the analysis				
Hip Abduction						
Physical Exercise interventions	47 (n=1,309)	-1.96 (-2.32, -1.59) I² = 93%	0.54 (0.44, 0.65) I² = 40%	-0.98 (-0.96, -0.98)	-0.27 (-0.26, -0.28)	Moderate

Endurance training	19 (n=387)	-1.63 (-2.16, -1.11) I² = 90%	0.57 (0.34, 0.81) I² = 60%	-0.91 (-0.93, -0.89)	-0.40 (-0.41, -0.39)	Low
Strength training	26 (n=886)	-2.15 (-2.63, -1.68) I² = 94%	0.56 (0.42, 0.70) I² = 43%	-0.99 (-0.98, -0.99)	-0.26 (-0.26, -0.25)	Moderate
Power training	1 (n=18)	Data did not permit the analysis				
Multimodal programmes	12 (n=255)	-2.08 (-2.70, -1.46) I² = 87%	0.52 (0.26, 0.78) I² = 51%	-0.34 (-0.45, -0.22)	-0.11 (-0.22, -0.01)	Low
Non-Exercise-Based interventions	11 (n=196)	Data did not permit the analysis				
Hip Adduction						
Physical Exercise interventions	14 (n=458)	-1.94 (-2.83, -1.05) I² = 97%	0.30 (-0.00, 0.60) I² = 77%	-0.06 (-0.15, 0.03)	-0.05 (-0.14, 0.04)*	Very Low
Endurance training	2 (n=60)	Data did not permit the analysis				
Strength training	6 (n=283)	Data did not permit the analysis				
Non-Exercise-Based interventions	3 (n=57)	Data did not permit the analysis				
Hip External Rotation						
Physical Exercise interventions	29 (n=925)	-1.83 (-2.27, -1.39) I² = 91%	0.51 (0.36, 0.66) I² = 29%	-0.03 (-0.09, -0.04)	-0.00 (-0.07, 0.06)*	Moderate
Endurance training	6 (n=167)	Data did not permit the analysis				
Strength training	19 (n=727)	-1.88 (-2.32, -1.44) I² = 92%	0.49 (0.33, 0.65) I² = 42%	0.11 (0.04, 0.18)	0.02 (-0.05, 0.09)*	High
Multimodal programmes	9 (n=204)	-2.13 (-2.75, -1.47) I² = 86%	0.58 (0.22, 0.94) I² = 67%	-0.07 (-0.21, 0.07)	-0.03 (-0.16, 0.11)*	Low
Non-Exercise-Based interventions	5 (n=91)	Data did not permit the analysis				
Hip Internal Rotation						
Physical Exercise interventions	13 (n=554)	-2.35 (-3.22, -1.49) I² = 97%	0.32 (0.10, 0.53) I² = 55%	-0.64 (-0.69, -0.59)	-0.24 (-0.29, -0.19)	Moderate
Endurance training	1 (n=32)	Data did not permit the analysis				
Strength training	10 (n=503)	Data did not permit the analysis				
Multimodal programmes	1 (n=11)	Data did not permit the analysis				
Non-Exercise-Based interventions	2 (n=42)	Data did not permit the analysis				

Hip Extension						
Physical Exercise interventions	21 (n=748)	-1.95 (-2.46, -1.44) I^2 = 94%	0.44 (0.28, 0.59) I^2 = 24%	-0.68 (-0.72, -0.64)	-0.11 (-0.14, -0.07)	Moderate
Endurance training	4 (n=95)	Data did not permit the analysis				
Strength training	15 (n=655)	Data did not permit the analysis				
Power training	1 (n=18)	Data did not permit the analysis				
Multimodal programmes	6 (n=129)	-2.56 (-3.35, -1.77) I^2 = 81%	0.63 (0.19, 1.08) I^2 = 66%	0.36 (0.20, 0.50)	0.14 (-0.01, 0.29)*	Low
Non-Exercise-Based interventions	4 (n=68)	Data did not permit the analysis				
General						
Physical Exercise interventions	77 (n=1,957)	-1.80 (-2.05, -1.55) I^2 = 91%	0.47 (0.35, 0.59) I^2 = 71%	-0.55 (-0.58, -0.52)	-0.25 (-0.28, -0.22)	Low
Endurance training	43 (n=917)	-1.46 (-1.73, -1.18) I^2 = 86%	0.51 (0.35, 0.68) I^2 = 67%	-0.49 (-0.54, -0.44)	-0.18 (-0.23, -0.14)	Low
Strength training	34 (n=1,048)	-2.07 (-2.44, -1.70) I^2 = 92%	0.41 (0.28, 0.55) I^2 = 53%	-0.95 (-0.96, -0.94)	-0.27 (-0.27, -0.26)	Low
Power training	1 (n=18)	Data did not permit the analysis				
Multimodal programmes	35 (n=747)	-1.58 (-1.93, -1.23) I^2 = 88%	0.44 (0.29, 0.59) I^2 = 50%	-0.52 (-0.57, -0.47)	-0.15 (-0.21, -0.11)	Moderate
Non-Exercise-Based interventions	29 (n=586)	Data did not permit the analysis				

* Non-significant association. Abbreviations: k = number of clusters; n = number of participants; ES = effect size; CI = confidence interval.

- B: Subgroup analyses for the associations between changes in Function versus Muscle Strength.

Muscle group	k Clusters (n)	Function [ES (CI 95%, I²)]	Muscle Strength [ES (CI 95%, I²)]	Correlational coefficient [r (CI 95%)]	Regression coefficient [β (CI 95%)]	Quality of Evidence
Knee Extension						
Physical Exercise interventions	58 (n=1,534)	1.39 (1.17, 1.61) $I^2 = 87\%$	0.57 (0.46, 0.68) $I^2 = 53\%$	0.73 (0.71, 0.75)	0.17 (0.15, 0.20)	Low
Endurance training	26 (n=558)	1.31 (0.94, 1.67) $I^2 = 86\%$	0.61 (0.43, 0.80) $I^2 = 54\%$	0.43 (0.36, 0.50)	0.13 (0.06, 0.20)	Low
Strength training	30 (n=953)	1.29 (1.04, 1.53) $I^2 = 82\%$	0.49 (0.35, 0.62) $I^2 = 47\%$	0.89 (0.88, 0.90)	0.13 (0.12, 0.14)	Moderate
Power training	1 (n=18)	Data did not permit the analysis				
Multimodal programmes	25 (n=491)	1.06 (0.87, 1.25) $I^2 = 48\%$	0.50 (0.32, 0.68) $I^2 = 47\%$	-0.04 (-0.16, 0.08)	-0.00 (-0.09, 0.08)*	Moderate
Non-Exercise-Based interventions	10 (n=266)	Data did not permit the analysis				
Knee Flexion						
Physical Exercise interventions	22 (n=546)	1.18 (0.90, 1.47) $I^2 = 76\%$	0.57 (0.38, 0.76) $I^2 = 48\%$	0.14 (0.06, 0.22)	0.02 (-0.06, 0.11)*	Moderate
Endurance training	8 (n=146)	Data did not permit the analysis				
Strength training	11 (n=363)	Data did not permit the analysis				
Multimodal programmes	8 (n=133)	Data did not permit the analysis				
Non-Exercise-Based interventions	3 (n=121)	Data did not permit the analysis				
Hip Abduction						
Physical Exercise interventions	45 (n=1,244)	1.37 (1.15, 1.58) $I^2 = 82\%$	0.55 (0.43, 0.66) $I^2 = 39\%$	0.97 (0.97, 0.97)	0.14 (0.13, 0.14)	Moderate
Endurance training	14 (n=293)	1.18 (0.86, 1.49) $I^2 = 66\%$	0.65 (0.35, 0.94) $I^2 = 65\%$	0.88 (0.85, 0.90)	0.19 (0.16, 0.21)	Low
Strength training	28 (n=926)	Data did not permit the analysis				
Power training	1 (n=18)	Data did not permit the analysis				

Multimodal programmes	11 (n=235)	1.11 (0.79, 1.43) I^2 = 61%	0.43 (0.20, 0.66) I^2 = 31%	0.03 (-0.10, 0.16)	0.00 (-0.13, 0.13)*	Moderate
Non-Exercise-Based interventions	6 (n=104)	Data did not permit the analysis				
Hip Adduction						
Physical Exercise interventions	10 (n=328)	1.28 (0.91, 1.65) I^2 = 69%	0.40 (0.12, 0.69) I^2 = 55%	0.06 (-0.05, 0.17)	0.01 (-0.10, 0.12)*	Moderate
Strength training	6 (n=283)	Data did not permit the analysis				
Non-Exercise-Based interventions	2 (n=25)	Data did not permit the analysis				
Hip External Rotation						
Physical Exercise interventions	29 (n=937)	1.18 (0.99, 1.37) I^2 = 65%	0.47 (0.34, 0.59) I^2 = 27%	0.15 (0.09, 0.21)	0.01 (-0.05, 0.07)*	High
Endurance training	9 (n=215)	Data did not permit the analysis				
Strength training	19 (n=727)	1.17 (0.96, 1.37) I^2 = 61%	0.47 (0.31, 0.63) I^2 = 39%	-0.36 (-0.42, -0.30)	-0.02 (-0.09, 0.04)*	High
Multimodal programmes	9 (n=204)	1.12 (0.76, 1.49) I^2 = 65%	0.59 (0.23, 0.95) I^2 = 68%	-0.27 (-0.39, -0.14)	-0.06 (-0.18, 0.07)*	Low
Non-Exercise-Based interventions	4 (n=77)	Data did not permit the analysis				
Hip Internal Rotation						
Physical Exercise interventions	10 (n=491)	1.17 (0.87, 1.48) I^2 = 68%	0.24 (0.01, 0.46) I^2 = 47%	0.96 (0.95, 0.97)	0.06 (0.05, 0.07)	High
Strength training	8 (n=472)	1.05 (0.78, 1.31) I^2 = 64%	0.20 (-0.04, 0.44) I^2 = 54%	0.90 (0.88, 0.92)	0.05 (0.03, 0.07)	Moderate
Multimodal programmes	1 (n=11)	Data did not permit the analysis				
Non-Exercise-Based interventions	1 (n=10)	Data did not permit the analysis				
Hip Extension						
Physical Exercise interventions	22 (n=738)	1.08 (0.89, 1.27) I^2 = 55%	0.39 (0.24, 0.55) I^2 = 20%	0.42 (0.36, 0.48)	0.02 (-0.04, 0.08)*	Moderate
Endurance training	3 (n=75)	Data did not permit the analysis				
Strength training	15 (n=635)	Data did not permit the analysis				
Power training	1 (n=18)	Data did not permit the analysis				
Multimodal programmes	5 (n=109)	Data did not permit the analysis				

Non-Exercise-Based interventions	3 (n=48)	Data did not permit the analysis				
General						
Physical Exercise interventions	68 (n=1,731)	1.41 (1.19, 1.64) I^2 = 89%	0.46 (0.35, 0.56) I^2 = 57%	0.82 (0.80, 0.83)	0.24 (0.22, 0.25)	Low
Endurance training	30 (n=618)	1.33 (0.93, 1.73) I^2 = 90%	0.51 (0.34, 0.69) I^2 = 56%	0.90 (0.88, 0.91)	0.35 (0.33, 0.36)	Moderate
Strength training	36 (n=1,088)	1.26 (0.97, 154) I^2 = 88%	0.36 (0.22, 0.50) I^2 = 52%	0.74 (0.71, 0.77)	0.16 (0.13, 0.19)	Low
Power training	1 (n=18)	Data did not permit the analysis				
Multimodal programmes	26 (n=504)	0.98 (0.72, 1.24) I^2 = 73%	0.39 (0.23, 0.56) I^2 = 39%	0.10 (0.01, 0.19)	0.01 (-0.07, 0.10)*	Moderate
Non-Exercise-Based interventions	17 (n=391)	Data did not permit the analysis				
